# Upstream Interventions to Promote Oral Health and Reduce Oral Health Inequalities: A Scoping Review

**DOI:** 10.1111/cdoe.70049

**Published:** 2025-12-29

**Authors:** Michelle Stennett, Eleanor Dawson, Marisza Hijryana, Paul Cannon, Blanaid Daly, Lorna Macpherson, Richard G. Watt

**Affiliations:** ^1^ University College London (UCL) London UK; ^2^ University of Birmingham Birmingham UK; ^3^ University of Glasgow Scotland UK; ^4^ Trinity College Dublin Dublin Republic of Ireland

**Keywords:** inequalities, intervention, oral health, policy, public health, upstream

## Abstract

**Objectives:**

This scoping review aimed to map global evidence on upstream interventions which promote oral health and reduce socioeconomic inequalities in oral health.

**Methods:**

A review was undertaken in accordance with the Joanna Briggs Institute (JBI) methodology for scoping reviews and the Preferred Reporting Items for Systematic Reviews and Meta‐Analyses Extension for Scoping Reviews (PRISMA‐ScR) checklist. A multistranded comprehensive search strategy was employed to identify relevant studies. Article texts were retrieved and assessed for eligibility against the inclusion criteria. Key findings were extracted and summarised.

**Results:**

A total of 99 articles (74 empirical studies and 25 systematic, scoping and umbrella reviews) were included in the scoping review. The review findings revealed a limited number of upstream interventions specifically focused on promoting oral health and reducing oral health inequalities. Legislative and regulatory measures (e.g., advertising controls), fiscal measures (e.g., sugar‐sweetened beverage taxation) and specific oral health interventions (e.g., water fluoridation) have shown a positive impact on promoting oral health. In addition, fiscal measures, food subsidies targeted at low‐income groups and improvements to housing/work environments have proven effective in reducing socioeconomic inequalities in general health outcomes.

**Conclusions:**

Despite a very detailed and thorough search of the global literature, this scoping review identified a limited number of upstream interventions that specifically focused on improving oral health, and an even smaller number of upstream interventions that tackled oral health inequalities. However, the review did identify three levels of upstream intervention including: policies tackling the broader socio‐political determinants of health; policies combating non‐communicable diseases (NCDs) linked to oral health; and some specific interventions (e.g., water fluoridation) focusing on oral health. The upstream approach to prevention remains highly relevant to public health policy and provides a guiding principle for future strategic action to promote oral health and tackle oral health inequalities.

## 1 Introduction

Oral diseases are a significant global public health problem [1]. Despite being largely preventable, oral diseases remain highly prevalent, with almost half of the world's population (3.69 billion people) affected [2]. While the prevalence of dental caries decreased in many high‐income nations between 1990 and 2017, it has increased in many low and middle‐income countries, which is largely linked to economic development and increasing availability and consumption of free sugars [3].

Poor oral health can have a significant negative impact on individuals and society, including impacts on educational performance and employment prospects [4]. There are also significant economic costs associated with dental treatment and lost productivity, amounting to approximately $710 billion globally in 2019 [5]. Oral diseases disproportionately affect poorer, socially disadvantaged and marginalised groups [3], and there are stark socioeconomic gradients in the prevalence of oral conditions [6, 7]. These inequalities are avoidable, unfair and unjust in society [3].

For many decades, the global dental profession has primarily adopted individual, clinical (*downstream*) approaches to prevention. This traditional approach involves giving oral health advice, providing clinical preventive measures, such as applying topical fluorides, and delivering oral health education in schools and community settings. However, this individualistic clinical and behavioural preventive approach has failed to significantly reduce oral health inequalities or achieve sustainable improvements in oral health outcomes [8, 9, 10]. Moreover, evidence has demonstrated that downstream interventions may adversely result in increasing health inequalities between social groups (intervention generated inequalities), as they may provide more benefit for groups already advantaged and able to change their behaviours [11].

To address this issue, it is widely acknowledged that a combination of both downstream and upstream interventions is needed to effectively prevent oral diseases and promote oral health equity [3]. However, there is still uncertainty regarding what is meant by upstream intervention. Although the term *upstream* dates back to the 1970's when McKinlay first used the expression [12], there is a surprisingly limited literature on how to precisely define an upstream intervention. Differing definitions of upstream intervention are largely dependent on the different perspectives on the underlying determinants of health and health inequalities [13]. In general, upstream interventions are seen as population level policies such as fiscal measures, regulation and legislation (healthy public policies) that aim to create and maintain healthier and more equitable environments [13]. To address power imbalances and economic inequalities, upstream interventions can also include social and economic policies such as welfare benefits, employment rights and housing reforms [14, 15, 16, 17].

This scoping review was conducted to systematically map the global research evidence on upstream interventions to promote oral health and reduce socioeconomic inequalities in oral health. The review aimed to answer two questions:

1. What upstream interventions have been implemented to promote oral health and reduce socio‐economic inequalities in oral health?
2. What relevant and related public health upstream interventions have been implemented to promote health and reduce socio‐economic health inequalities that could have an impact on promoting oral health and reducing oral health inequalities?

## 2 Methods

A scoping review was conducted in accordance with the Joanna Briggs Institute (JBI) methodology for scoping reviews [18] and reported in alignment with the Preferred Reporting Items for Systematic Reviews and Meta‐Analyses Extension for Scoping Reviews (PRISMA‐ScR) checklist [19]. The scoping review protocol was registered prospectively with the Open Science Framework on 17th August 2021 (https://osf.io/5zy9g/).

A preliminary search was conducted in SCOPUS, Google Scholar, Cochrane Database of Systematic Reviews and *JBI Evidence Synthesis*, but no ongoing or previously published systematic reviews or scoping reviews were found on the topic of upstream interventions.

A multistranded comprehensive search strategy was used for this review [20]. The dental and oral health elements were adapted from Waldron et al. (2017) [21] and Arora et al. (2019) [22], the sugar tax strand element of the search was adapted from Pfinder et al. (2020) [23], and the equity‐focused element from Prady et al. (2018) [24]. All authors reviewed and approved the combined search strategies. The search strategy was utilised across a range of relevant subject‐specific and multi‐disciplinary electronic databases. These were: ASSIA (ProQuest), CINAHL and PsycINFO (both EBSCOhost), Medline and Embase (both OvidSP), the Cochrane Database of Systematic Reviews (via the Cochrane Library) and Scopus. The following grey literature and thesis sources were searched: OpenGrey, WorldCat, NICE Evidence search and British Library e‐theses online service (EThOS), Trip and National Technical Information Services: Technical Reports. The websites of the following relevant organisations were also searched and browsed for relevant publications and reports: The Health Foundation, World Health Organization, The World Bank and UK Government Research and Statistics. A full copy of the Embase search strategy and search terms is available at: https://osf.io/5zy9g/.

All information sources were initially searched between September and October 2021, with updated searches conducted in November 2023 and June 2025. The inclusion criteria were: (a) Populations—interventions that include any population (children, adults and older adults) in any context/country; (b) Interventions—published studies that have implemented and evaluated population‐level upstream health policies such as fiscal measures, regulation and legislation operating at local, regional, or national levels. Wider social and economic policies tackling socioeconomic inequalities such as welfare benefits and employment rights were also included; (c) Outcomes—literature reporting on a range of health and oral health outcomes (clinical and subjective), oral health related behaviours and socio‐economic inequality measures; (d) Study Design—experimental, quasi‐experimental, before and after studies, observational studies (cohort, case control and cross‐sectional), scoping and systematic reviews of relevant upstream public health interventions; (e) Literature type—published peer reviewed and grey literature; (f) Language—title and abstracts published in English. A full paper not published in English but suitable for inclusion was translated; (g) Date—no date limit on the included literature. Furthermore, the exclusion criteria were: (a) Interventions—studies reporting on clinical interventions delivered in clinical settings and restricted to specific patient populations; (b) Outcomes—studies only reporting oral health knowledge and attitudes; (c) Study design—reviews of websites or industry documents.

All identified citations were compiled into *EndNote* and imported into the screening software *Rayyan* (http://rayyan.qcri.org). Duplicate records were removed using *EndNote*'s de‐duplication tool, followed by a manual screen. Articles were screened by two independent reviewers against the inclusion criteria. Disagreements on the eligibility of texts between reviewers were discussed by the research team to determine a consensus. Full texts of eligible articles were included in the final review. Tabular data extraction sheets were developed by the research team to include the main characteristics for both the empirical studies and systematic reviews. Articles were grouped within the tables in subsections according to the nature of the upstream intervention evaluated.

## 3 Results

### 3.1 Selection of Sources of Evidence

**FIGURE 1:**
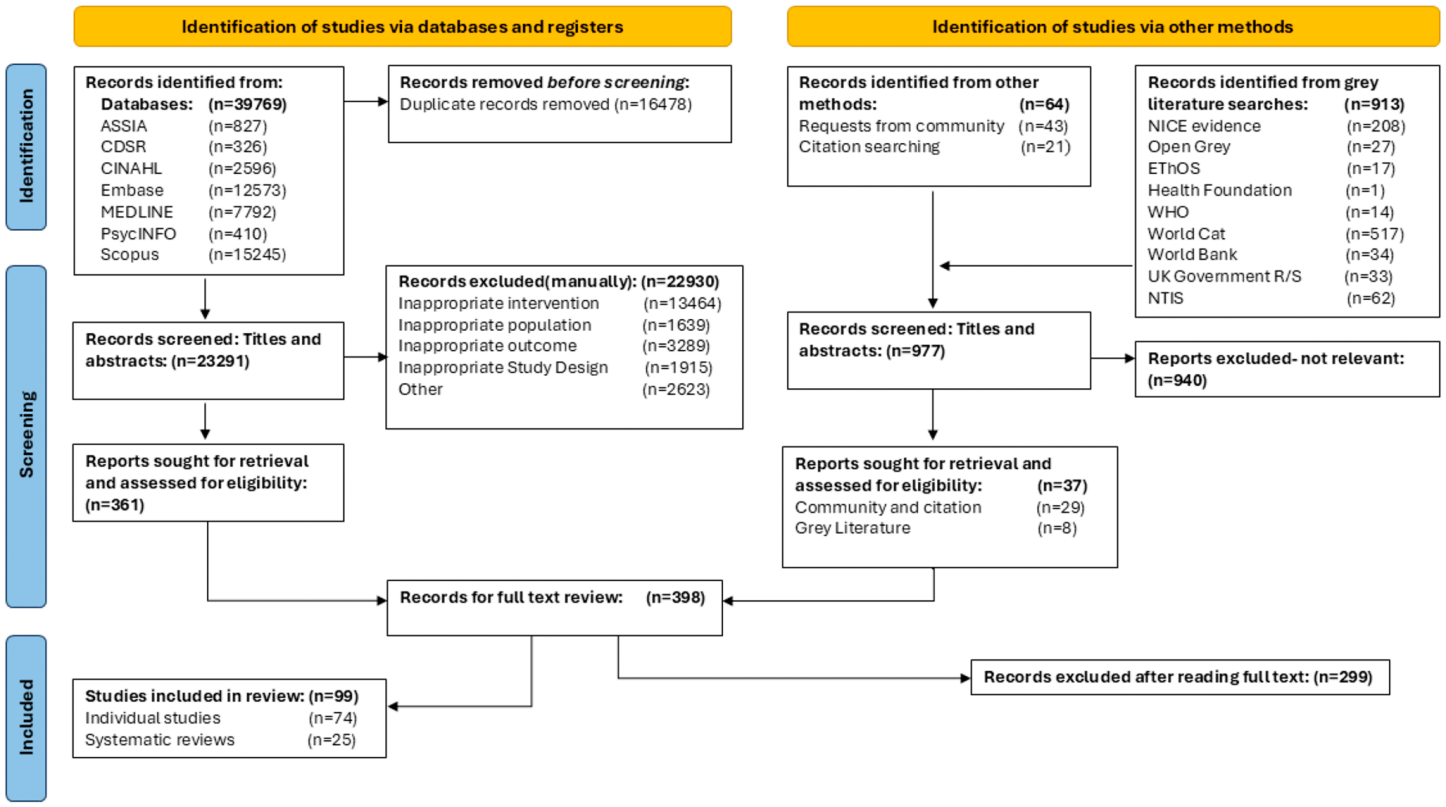
Prisma flow chart.

In relation to the scoping review, the database searches identified a total of 23 291 articles after de‐duplication. After reviewing titles and abstracts, 22 930 articles were excluded, and 361 articles were included for full‐text review. The grey literature searches, citation searches and requests from academic peers identified 977 articles. Following screening of titles and abstracts, 940 grey literature articles were excluded, and 37 articles were included for the full‐text review. After full text reading of the 398 citations identified through all search methods, a further 299 articles were excluded, leaving a total of 99 articles that were included in this scoping review. Figure 1 displays the PRISMA flowchart which outlines the selection process in detail.

This review included 74 individual empirical studies, and their main characteristics are provided in Figure S1 and Figure S2. These studies were published between 1980 and 2025, with most being published between 2010 and 2025. The studies were conducted in various countries in Asia, Australasia, Europe, Central America and South America. Surprisingly, no studies were conducted in Africa, although this may illustrate bias in our information sources. Most of the studies were published in English, except for one Spanish article and one German article, which were assessed by Spanish and German speakers to determine their inclusion in this review.

What upstream interventions (including related public health interventions that could have an impact on oral health) have been implemented to promote oral health and reduce socioeconomic inequalities in oral health?

### 3.2 Empirical Studies

**FIGURE 2:**
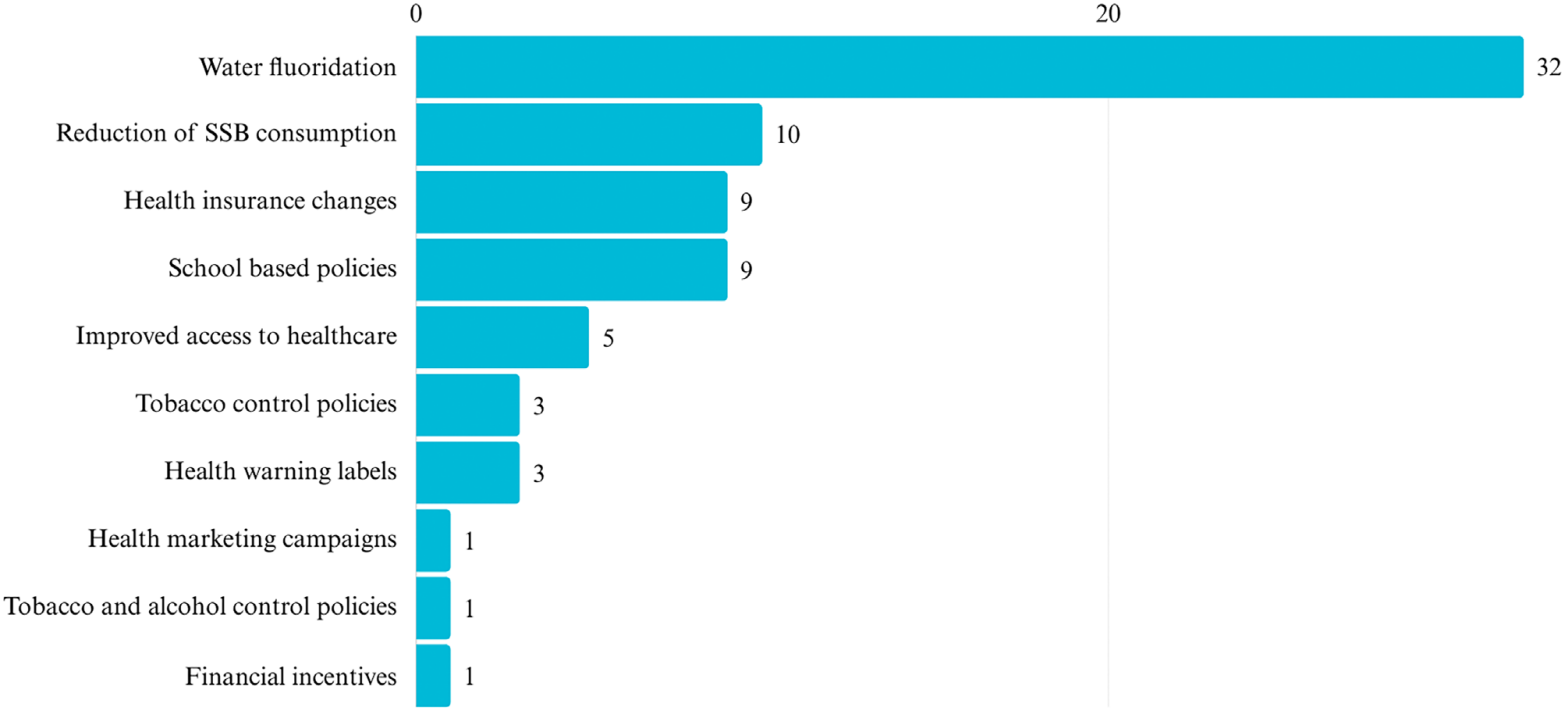
Nature of upstream interventions (empirical studies) *N* = 74.

Based upon the nature of the intervention evaluated, the 74 empirical studies were grouped under 10 sub‐headings: water fluoridation (*n* = 32), improving access to health care (*n* = 5), tobacco control policies (*n* = 3), tobacco and alcohol control policies (*n* = 1), school‐based policies (*n* = 9), changes to health insurance (*n* = 9), health marketing campaigns (*n* = 1), health warnings on labels (*n* = 3), interventions to reduce sugar‐sweetened beverage consumption (*n* = 10) and financial incentives (*n* = 1) (Figure 2). Full details of each study are presented in Table S1 and are summarised in Table 1.

**TABLE 1:** Summary of key findings: Empirical studies.

Intervention type	Number of studies	Outcomes examined	Summary of key findings
Water fluoridation (WF)	32	*Clinical*: Caries prevalence, disease experience (dmft, dmfs/DMFT, DMFS, ICDAS II code 5, visible non cavitated dentine caries), dental fluorosis (Dean's index) *Non‐clinical*: Caries related dental visits, receipt of caries related general anaesthesia, years of life with caries as a disability (DALYS), quality adjusted life years, reduction in dental treatment cost, cost effectiveness, lifetime treatment cost of caries, potential treatment savings, dental caries prevented attributable to WF, minimum population needed for benefits of WF to exceed WF treatment costs	Reductions in caries experience attributed to water fluoridation across all socioeconomic strata. Although some evidence of reduction in social inequalities in caries prevalence, inequalities persist WF: Reduced dental care visits Was cost effective (may be less effective in smaller communities < 1000 persons) May provide better caries preventative effect than fluoridated salt (0.1–1.2 ppm optimal range)
Improved access to healthcare	5	Mortality rate (oral cancer), uptake of dental check‐ups, dental service utilisation, perceived need and availability of dental services, SE inequalities in use of dental services	Some evidence to show interventions to improve dental care access (provision of free or subsidised dental coverage) led to modest increases in service utilisation, but no significant sustained reductions in inequalities of service use across income groups
Tobacco control policies	3	Knowledge of legislation prohibiting tobacco sales to minors, Sale of tobacco products to minors, estimates of proportions of avoidable cancer cases, implementation of WHO MPOWER tobacco control policy measures	Implementation of policies to promote knowledge of legislation prohibiting tobacco sales to minors led to significant reductions in sales of tobacco products to and by minors and reduced absence of warning boards at points of sale. Tobacco control policies (e.g., plain packaging, price increases, marketing bans) are estimated to reduce smoking related cancer cases (13% reduction over 30 years) Countries with more implementation of MPOWER policies also have decreasing lip and oral cavity burden
Tobacco and alcohol control policies	1	*Tobacco*: Monitoring of use, protection from smoke, warning of dangers, quitting tobacco, enforcing advertising bans, raising taxes (WHO MPOWER) *Alcohol*: policies restricting time/day of sales, regulation of advertising, indirect taxes	Limited evidence of a correlation between oral cancer mortality and weak implementation of tobacco and alcohol control policies
School based policies	9	*Clinical*: Dental sealants, DMFT, presence of new carious lesion, Oral hygiene status *Non‐clinical*: Exposure to sugar products, sale of sugary products, Total daily sugar intake/frequency, 6‐day weighted food intake, provision of oral health protection items/healthy snacks/drinking water, change in oral health promoting policies, attitudes and access to intoxicants, school health services, eating habits in school, school food standards/school food plan implementation, unhealthy snacking in school	Some school based oral health programs (fissure sealant programs, restriction of sugary food availability, provision of drinking water/healthy snacks) associated with: Increased fissure sealant placement among low‐income children Significant decrease in exposure of pupils to and consumption of sugary products Significant increase in oral health promotion policies Significant increase in mean number of sound teeth (over 2 years) in schools in low socioeconomic areas No evidence that school food standards legislation positively influences nutritional intake However, only limited evidence of reduction in SEP based inequalities of sugary product sales in schools or DMFT
Health insurance changes	9	*Clinical*: BPE scores, Presence of FPM either with sealant, single crown, or DMF score, % receiving scaling, presence of caries or sealants *Non‐clinical*: self‐rated poor oral health, SE oral health inequalities (income), Unmet dental need, dental expenditure, dental service utilisation, Life expectancy/expected years of life lost (oral cancer)	The expansion of health insurance to include more items (e.g., dental scaling, dental sealants, dentures, implants) or universal coverage may have led to: Increases in the rate of healthy periodontal status Increased fissure sealants and lower caries rates Increased provision of dental scaling Improvements in self‐rated oral health for younger adults Increased diagnoses and improved life expectancy for oral cancer Income gradients largely persisted for most outcomes but were somewhat alleviated for untreated caries in children and unmet dental need
Health marketing campaigns on sugar reduction	1	Total sugars, NME sugars, % contribution of sugars to energy intake, change in energy/fat intake	Modest reductions in mean sugar intake following media marketing campaign (TV, billboard, digital advertising) were not sustained at 12 months post intervention
Health warning labels (HWL) on tobacco and food products	3	Perceived risk of smoking, reduction in desire to smoke, ‘believability’ of HWL, desirability/attractiveness of packaging with HWL, perception of harm associated with HWL, Caries prevented, caries treatment cost avoided (DALYS), productivity loss averted.	Some limited evidence that HWL's (larger size, front of package labelling, plausible messaging) may lead to improved behavioural outcomes (may reduce the appeal of cigarettes, increase the intention to abstain from smoking, hypothetically reduce caries increment)
Sugar sweetened beverage consumption interventions	10	*Clinical*: Caries incidence (DMFT/DMFS), change in BMI *Non‐clinical*: Potential cost savings from avoided treatment, (QALYS), indirect costs from lost earnings due to care, change in dental visits related to caries, prevention of tooth loss, caries free tooth years, life expectancy, slope index of inequality in life expectancy	Data mainly from modelling studies estimate that restricting the size of SSB, increasing the cost of SSB, implementing SSB taxes may lead to reductions in sugar intake, BMI, caries incidence, hospital admissions for carious tooth extractions, and long‐term improvements in life expectancy in all children (0–18 years). Modelling and data analysis evidence suggests greater benefit for those living in more deprived areas with the potential to reduce caries inequalities. However, these interventions may be limited unless they are utilised in combination with other strategies to reduce sugar intake from non SSB sources
Financial incentives	1	*Clinical*: Maternal 6‐week post‐partum check, paediatric dental visits, well‐check visits *Non‐clinical*: financial coaching sessions	No evidence of financial incentives increasing the likelihood of clinical checks but may increase the likelihood of financial coaching session uptake

Thirty‐two papers [25, 26, 27, 28, 29, 30, 31, 32, 33, 34, 35, 36, 37, 38, 39, 40, 41, 42, 43, 44, 45, 46, 47, 48, 49, 50, 51, 52, 53, 54, 55, 56] from eight countries (Australia, UK, South Korea, Israel, USA, Brazil, Uruguay and New Zealand) showed significant reductions in caries experience across all socioeconomic groups exposed to fluoridated water supplies. The studies also demonstrated positive effects of water fluoridation on reducing dental visits, with impacts lasting into adulthood. There was evidence to support the cost effectiveness of water fluoridation and although some studies reported reductions in socioeconomic inequalities in caries experience [31, 40, 51], other studies reported limited or no effect of water fluoridation on inequalities in caries experience [27, 43, 56].

Five studies [57, 58, 59, 60, 61] conducted in Brazil, UK, USA and Finland provided some limited evidence that policies/reforms designed to improve dental care access (provision of free or subsidised dental coverage) led to modest increases in service use but had no significant sustained impact on reducing inequalities in service use across income groups.

Three studies [62, 63, 64] (one study was based on a simulation modelling exercise in Germany) [63] identified the potential of various tobacco restriction policies to improve health. The simulation model estimated a reduction in smoking‐related cancer cases (including oral cancers) of 13% over 30 years following implementation of policies to increase cigarette prices, ban marketing and introduce plain cigarette packaging. Implementation of WHO MPOWER tobacco control measures was associated with decreased lip and oral cancer burden in several countries across South America [64]. The remaining study [62] reported reductions in tobacco sales correlated with knowledge of the law prohibiting tobacco sales to minors in India but did not provide sufficient methodological details.

One ecological study [65] involving several Latin American countries assessed the implementation of both tobacco and alcohol control policies and noted a correlation of higher oral cancer mortality rates in countries with less progress in executing tobacco and alcohol control policies.

The evaluation of school‐based policies was reported in nine studies [66, 67, 68, 69, 70, 71, 72, 73, 74] conducted in the USA, Finland, UK, Brazil and France. The preschool and school‐based policies (national recommendations for restriction of sugary food availability, provision of drinking water/healthy snacks and fissure sealant programmes) were associated in some studies with significant improvements in the provision of oral health promotion policies in schools and significant decreases in exposure and consumption of sugary products by children in preschool and school settings. However, there was limited evidence of a reduction in socioeconomic inequalities in oral health.

Nine studies [75, 76, 77, 78, 79, 80, 81, 82, 83], all conducted in South Korea and Taiwan evaluated the impact of expanding national health insurance programmes to cover a wider range of oral health services (such as dental scaling, dental sealants, dentures and implants) on oral health outcomes. The findings indicated improvements in periodontal status, self‐rated oral health for younger adults, diagnoses of oral cancer and lower caries rates. While modifications to health insurance had minimal impact on oral health inequalities across most outcomes assessed, they partially attenuated income gradients for untreated caries in children and levels of unmet dental needs.

One UK study [84] assessed the impact of health marketing campaigns over 12 months on dietary intake (including free sugars) and found modest reductions in sugar consumption up to 10 months post campaign, but no evidence beyond this of long‐term sustainability of reduced sugar consumption.

Three studies [85, 86, 87] from Turkey, Mexico and Germany evaluated health warning labels applied to tobacco and food items and found some limited evidence to suggest these may potentially reduce the appeal of smoking and caries increment.

Ten recent studies [88, 89, 90, 91, 92, 93, 94, 95, 96, 97] published between 2019 and 2025 analysed sugar sweetened beverage (SSB) consumption interventions in Thailand, Netherlands, Mexico, India, UK, USA and New Zealand. These studies assessed the impact of taxation, restrictions on serving sizes and price increase policies on a range of clinical (DMF and BMI) and economic outcomes mainly through simulation modelling. Increasing the cost of SSBs, implementing taxes and restricting the size of SSBs led to reductions in free sugar intake, BMI and caries incidence, particularly for disadvantaged populations, thereby narrowing socioeconomic inequalities [92, 96, 97].

One small study [98] assessed the impact of providing financial incentives to low‐ income mothers to increase child attendance for medical and dental visits but found no significant impact from this intervention on attendance rates.

### 3.3 Systematic Reviews

**FIGURE 3:**
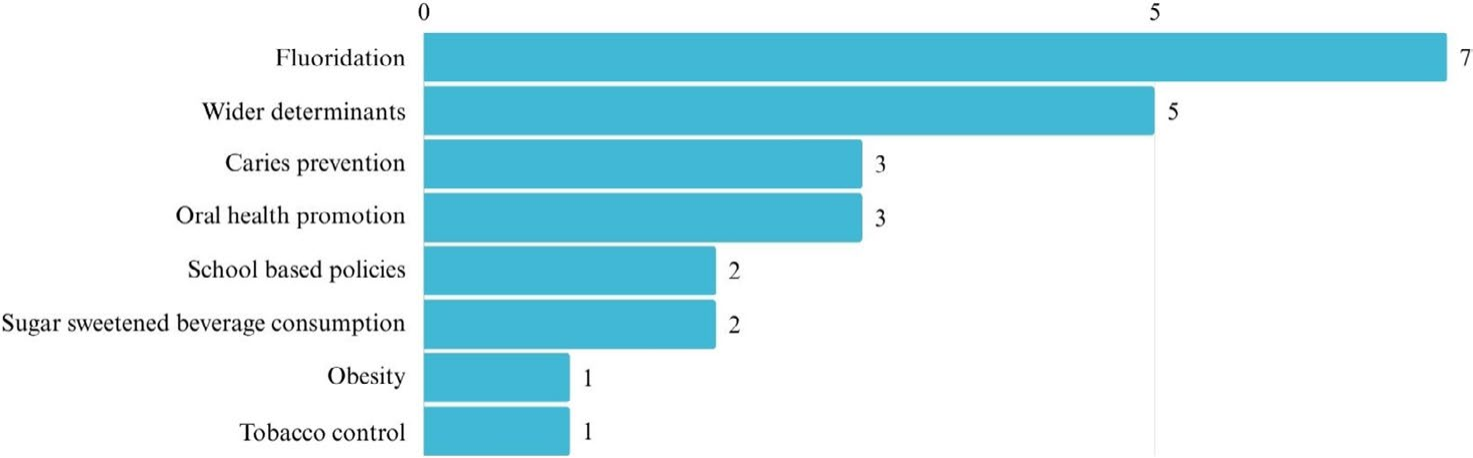
Nature of upstream interventions (systematic reviews and scoping review) *N* = 24.

Twenty‐four systematic and scoping reviews, and one umbrella review were identified and included. The topic areas covered by the systematic reviews and scoping review included interventions focusing on the broader determinants of health (*n* = 5), caries interventions (*n* = 3), interventions to reduce obesity (*n* = 1), fluoride interventions (*n* = 7), school‐based policies (*n* = 2), interventions to reduce sugar sweetened beverage consumption (*n* = 2), oral health promotion interventions (*n* = 3) and tobacco control policies (*n* = 1) (Figure 3). Full details of each systematic review are presented in Table S2 and are summarised in Table 2.

**TABLE 2:** Summary of key findings: Systematic reviews.

Intervention type	Number of reviews	Nature of interventions	Summary of key findings
Wider determinants of health/mixed interventions	5	Housing/environment (e.g., rent assistance), Transport (e.g., traffic calming measures), Improving access to health and social care, early years interventions for children, Welfare interventions, Agricultural interventions, Water fluoridation, Health education/communication, school or community‐based interventions, Fiscal interventions, clinical prevention (e.g., fissure sealants or fluoride varnish)	Generally unclear impact of mixed interventions on health inequalities. Heterogeneity of papers included in many reviews Some tentative evidence to support interventions in housing work/environment sector to reduce inequalities in disadvantaged groups Limited evidence supports early years interventions for improving child health outcomes for the most disadvantaged Fiscal interventions (e.g., tobacco pricing) may reduce the risk of socioeconomic inequalities Multi component and tailored interventions around health financing have proved effective at reducing oral health inequalities in vulnerable populations Interventions such as mass media campaigns or smoking bans at work may increase socioeconomic inequalities Some limited evidence of reduction in socioeconomic inequalities from area‐based interventions such as changes to the physical environment
Caries prevention interventions	3	Dental sealants, water fluoridation, fluoride varnish/gel, oral health education	Whole population interventions (e.g., water fluoridation), early approaches, and interventions including broader organisation policies are more likely to reduce caries inequalities than target population or individual interventions
Obesity	1	Behavioural interventions in the workplace, environmental interventions	Limited low‐quality evidence to suggest that physical activity interventions targeted towards low‐income groups may reduce socioeconomic inequalities in obesity rates
Fluoridation	7	Water fluoridation Milk fluoridation Salt fluoridation Sugar fluoridation Topical fluorides (toothpaste, gel, varnish, mouthwash)	Water fluoridation is the most socially equitable means of providing fluoride to communities for caries prevention Economic benefit of WF exceeds the cost of the implementation Implementation of water fluoridation may lead to slight reduction in caries and proportion of caries free children Insufficient evidence to determine the effect of water fluoridation cessation on caries Not enough evidence to show an impact of water fluoridation on reducing socioeconomic disparities in caries Low quality evidence‐ fluoridated milk effective in reducing caries Unable to assess quality of evidence regarding fluoridated sugar
School based policies	2	Nutrition (healthy eating), increasing physical activity, supporting good mental health, smoking cessation, cognitive behavioural techniques, nurse delivered counselling, parenting skills/role modelling, decreasing screen media use	Some limited evidence of reduction in socioeconomic oral health inequalities (2 studies), with greater effects demonstrated in schools within areas of higher disadvantage Some evidence to suggest reductions in sugar sweetened beverage consumption through educational and behavioural interventions
Sugar sweetened beverage (SSB) consumption interventions	2	Reformulation to reduce sugar levels, labelling interventions, economic interventions (e.g., pricing), whole food supply, nutritional standards, home based interventions.	Low quality evidence to suggest that sugar reformulation can reduce sugar intake. Labelling (e.g., traffic light), retail price increases, promotion of healthier alternative drinks, exclusion of SSBs of government food benefits, and community campaigns may be effective in reducing SSB consumption
Oral health promotion	3	Preventative interventions including fluoride, smoking cessation, oral health education, health promoting school policies, Restricting sugar consumption, sealant, or fluoride programs	Evidence of reduction in oral health inequalities with water fluoridation, fluoride toothpaste Models of health promotion which used indigenous community participation in policy changes may reduce oral health inequalities
Tobacco control policies	1	Bans on tobacco advertising, promotion, and sponsorship	Evidence of reduction in smoking prevalence and initiation but no evidence of an effect on smoking cessation

Five systematic reviews [11, 99, 100, 101, 102] evaluated interventions that focused on addressing the broader social determinants of health (water and sanitation; agriculture and food; access to health and social care services; early years interventions; unemployment and welfare; working conditions; housing and living environment; education; and transport). Overall, the evidence quality was poor, especially in studies focused on health inequalities reduction. However, some tentative evidence suggested that interventions in housing and work environment reduced health inequalities [99], and early years interventions for children may improve health outcomes for the most disadvantaged [102]. Evidence also indicated that fiscal measures including reducing price barriers and taxation on tobacco products had a positive effect on reducing socioeconomic inequalities in health [11]. Area‐based interventions that aimed to improve social and structural living conditions also demonstrated some limited evidence on reduction in socioeconomic health inequalities [101]. In contrast, mass media campaigns and smoking bans in the workplace increased health inequalities [11].

Two systematic reviews and one scoping review [103, 104, 105] found that population‐wide preventive interventions such as water fluoridation and interventions targeted at children such as supervised toothbrushing could lead to reductions in oral health inequalities, particularly if implemented at an early age [105]. Seven systematic reviews [106, 107, 108, 109, 110, 111, 112] assessed the effectiveness and cost effectiveness of various fluoride interventions. The quality of the evidence assessed was low to moderate and many studies were conducted in the 1970s and 1980s in high‐income countries when caries levels were much higher than at the present time. The results showed moderate evidence to support water fluoridation as an effective measure to reduce caries [107] but insufficient evidence to demonstrate reductions in socioeconomic inequalities in caries experience [112].

One review [113] examined workplace interventions to reduce inequalities in obesity prevalence. Although some physical activity interventions directed at those in lower occupational groups showed promise, the majority of studies had no impact on inequalities.

Two reviews [114, 115] evaluated the effect of school‐based health promotion on health inequalities among children and young people in several countries. The studies found limited evidence of a reduction in sugar consumption in schools and in socio‐economic inequalities in oral health in schools within disadvantaged areas. Two reviews [116, 117] explored the effectiveness of various interventions on SSB consumption. The results highlighted that population‐level interventions, such as labelling, retail price increases, promotion of healthier alternatives, exclusion of SSBs from government food benefits and community campaigns, could be effective in reducing SSB consumption.

There were three systematic reviews [118, 119, 120] that examined the effectiveness of oral health promotion interventions. One review focused on general population oral health promotion studies, including fluorides and other preventive interventions [118]. Water fluoridation showed evidence of caries reduction and fluoride toothpaste was associated with better oral health in the absence of fluoridated water. Health promotion interventions that involved community participation reduced oral health inequalities and oral health education programmes were shown to improve oral health knowledge but there was less evidence on the impact on clinical outcomes [118]. A second review examined studies conducted in Latin American and Caribbean countries and mapped out the delivery of different interventions across these countries but provided limited information on the effectiveness of the interventions [120]. The third reviewed community‐based oral health programmes aimed at indigenous adolescents and identified a small number of good quality studies, concluding that there was a limited evidence base for culturally competent and effective community health programmes for this population group [119].

One review [121] assessed the effectiveness of tobacco advertising, promotion and sponsorship (TAPS) bans on reducing smoking prevalence, initiation and cessation across 70 countries. TAPS bans were associated with lower smoking initiation and prevalence but not associated with smoking cessation.

### 3.4 Umbrella Review of the Effects of Public Health Policies on Health Inequalities in High‐Income Countries

Thomson et al. [122] published an umbrella review of systematic reviews on the effects of public health policies on health inequalities in high‐income countries. The review summarised evidence of all types of primary and secondary prevention policies across seven public health domains (tobacco, alcohol, food and nutrition, reproductive health services, control of infectious diseases, environmental health and workplace regulations). Table 3 provides a summary of the review's findings.

**TABLE 3:** Matrix of population‐level preventative public health interventions (Adapted from Thomson et al., 2018).

Prevention type	Primary prevention			Secondary prevention	
Type of intervention	Fiscal measures	Regulation	Education, communication, and information	Preventive treatment	Screening
Description	Using market forces to change demand for products deemed healthy/unhealthy.	Making and enforcing regulation to encourage/discourage products and services deemed healthy/unhealthy.	Using mass media campaigns to encourage/discourage products and services deemed healthy/unhealthy.	Offering population‐wide measures to eradicate infectious diseases.	Offering age‐appropriate population level screening for certain diseases.
Domains					
Tobacco	Tobacco/cigarette tax ^b^ Combined fiscal, regulation, and educational approach^b^	Smoke free legislation in workplaces and/or enclosed public space^b^ Control on advertising and promotion of tobacco^a^ Combined fiscal, regulation, and educational approach^b^	Mass media smoking cessation campaigns^b^ Health warnings on cigarettes^b^ Combined fiscal, regulation, and educational approach^b^		
Alcohol	Lowering tax on alcohol^c^				
Food and nutrition	Tax on unhealthy food/soft drinks^a^ Food subsidy programmes for low‐income women^a^ Free school fruit subsidy^b^	Mandatory fortification to increase folate intake^b^ National salt reduction strategy^b^ Trans‐fatty acid ban in all food establishments and mandatory calorie labelling^b^ Water fluoridation^a^ Combined education campaign and nutritional labelling regarding salt^b^	General nutrition and/or physical activity information campaign^b^ Folic acid mass media campaign^c^ Health information campaigns (e.g., 5 a day)^b^ Sodium reduction information campaigns^b^ Combined education and nutritional labelling for sodium reduction^b^ National supervised tooth brushing programme^a^ Nutrition education programme^a^		
Reproductive health services			Reproductive cancer screening campaign^a^		Population cancer screening for female cancers^a^
Control of infectious diseases	Parent incentive scheme linked payment of childcare benefits and maternity allowance to immunisation status^a^	Schools required to request immunisation certificates^a^	Combined education and reminder/recall for vaccinations^b^	Targeted ‘reminder and recall’ systems. Universal/targeted vaccinations to indigenous adults/children^a^ Combined education and reminder/recall for vaccinations^b^	
Built environment		Traffic calming measures^a^ 20 mph zones^c^ Low emission zones in cities^c^			
Workplace regulations		Privatisation of utility industries^b^			

*Note:* Impact on inequalities: a– positive intervention effect, b – no intervention effect/inconclusive and c – negative intervention effect.

Twenty‐nine systematic reviews were identified reporting on 150 relevant primary studies. Overall, the findings across public health domains and the quality of the included reviews were mixed. The primary studies were generally poorly designed, with a high reliance on observational studies. Health inequalities were reduced through fiscal measures such as taxes on unhealthy food/soft drinks, food subsidies for low‐income women and financial incentives for parents to immunise their children. Regulatory measures on tobacco advertising, water fluoridation, immunisation certificates for children entering schools and traffic calming measures showed beneficial impacts on reducing health inequalities.

Specific education and screening programmes also demonstrated reductions in health inequalities. These included national campaigns that promote tooth brushing, nutrition education and reproductive cancer screening campaigns. Preventive interventions that showed positive findings in reducing health inequalities included targeted ‘reminder and recall’ systems for controlling infectious diseases, universal and targeted vaccination programs for Indigenous adults and children. However, some interventions were found to increase health inequalities; these included fiscal measures that lowered tax on alcohol and low emission zones in cities. Mass media campaigns that aimed to increase folic acid intake were also shown to increase health inequalities.

## 4 Discussion

As far as the authors are aware, this is the first scoping review of upstream interventions linked to oral health. Therefore, there are no directly comparable reviews to compare our findings with. A Cochrane review of community‐based population‐level oral health interventions only focused on downstream fluoride and educational programmes for children and did not include any upstream interventions [123].

Our extensive and detailed search and review of the relevant primary studies, systematic reviews, a scoping review and an umbrella review of the broader health inequalities literature has identified a dearth of scientific evidence for upstream interventions to promote oral health. Specifically, there were very few specific upstream interventions identified to reduce oral health inequalities. Our comprehensive literature search identified a total of 23 291 published articles and 977 grey literature. However, most of these papers were excluded, and only 74 empirical studies and 25 systematic, scoping and umbrella reviews were finally included in this scoping review.

The review found some promising upstream interventions, including regulatory and legislative policy measures to reduce tobacco consumption and promote better diets. Upstream fiscal measures and, in particular, increases in taxes on tobacco, alcohol and sugar sweetened beverages also showed encouraging findings on a range of outcomes. The scoping review also identified consistent evidence that water fluoridation had a positive impact on reducing population levels of dental caries.

Policies focusing on food and health at the local level within preschools and schools were highlighted to have an impact on reducing sugar consumption and dental caries. Some might question whether this healthy settings approach is a true upstream intervention, but the approach certainly uses regulatory measures to establish a supportive health promoting environment. This approach can be implemented in various settings such as preschools, schools, workplaces, hospitals and elderly care homes [124].

This scoping review found encouraging evidence from the systematic, scoping and umbrella reviews that assessed international evidence on upstream interventions to tackle health inequalities, although there were only a limited number of good quality empirical studies. Upstream interventions focused on improving quality of housing and the work environment demonstrated positive impacts in reducing health inequalities. Fiscal measures, food subsidy programmes targeting low‐income groups and regulatory measures to control tobacco advertising and promotion also showed encouraging findings in reducing health inequalities. However, the evidence for the impact of water fluoridation in reducing inequalities in dental caries levels is mixed, although some studies showed some evidence of its effectiveness among children. Our review findings are in accordance with recent Australian [125] and Canadian [126] technical reports that assessed the evidence for water fluoridation and its impact in reducing dental caries. These reviews found that water fluoridation reduced levels of dental caries in both child and adult populations but did not identify consistent evidence of the effect on reducing inequalities.

### 4.1 Strengths and Limitations

It is important to recognise the respective strengths and weaknesses of this scoping review. With expert guidance, the team undertook a very extensive and detailed systematic search of both the published and grey literature for this review. The search identified a range of relevant empirical studies, systematic reviews, a scoping review and an umbrella review that all provided valuable information. The team also assessed publications published in German and Spanish to widen the scope of the review beyond English‐language publications. The review, however, had some clear limitations. The exclusion criteria on what was deemed an upstream intervention could have been applied more stringently, as some of the empirical papers reviewed did not evaluate truly upstream interventions. Some of the grey literature may have been missed as, with limited resources available, the team were not able to contact all relevant stakeholders. In addition, the methodological strength and quality of the included articles was not assessed as part of the scope of this review.

The findings of this scoping review have important implications for the future training of the oral health workforce, policy development and future research. It is crucial that the future training of oral health professionals and specifically dental public health practitioners covers the upstream concept and highlights the limitations of only adopting a downstream approach. For oral health policy makers, although the upstream approach is not new, it remains a highly relevant concept to guide policy development and to implement population‐wide measures to tackle the underlying determinants of oral diseases and oral health inequalities. Policy makers need to be bolder and more radical in their policy choices, particularly in relation to tackling oral health inequalities. Greater policy emphasis is also needed in working with communities, civil society organisations and social movements to co‐produce upstream policies to ensure that they meet the needs of local people. Community empowerment is a powerful force for social, political and policy change especially for marginalised and disadvantaged groups in society who are all too often ignored and have no voice in policy decision making. The essence of an upstream approach is to focus on the broader population agenda and to integrate policy measures across the wider NCD, health equity and sustainable development agendas. The dearth of high‐quality studies evaluating upstream interventions has highlighted the need to further develop the research base for the upstream approach in relation to oral health improvement strategies and actions to promote oral health equity. There is growing recognition for the need to move beyond the narrow confines of the traditional randomised controlled trial which is not appropriate for the evaluation of complex upstream interventions [127]. A broader range of evaluation methodologies needs to be employed to capture and evaluate the context and complexity of upstream policies. Cluster trials that randomise groups or localities, natural experiments, difference‐in‐difference studies, regression discontinuity analysis and simulation modelling analyses are all potentially valuable methods to use to assess the impact, effectiveness and cost effectiveness of upstream interventions.

### 4.2 Conclusions

In conclusion, despite a very detailed and thorough search of the global literature, this scoping review identified a limited number of specific upstream interventions that focused on improving oral health and an even smaller number of upstream interventions that tackled oral health inequalities. However, the review did identify three levels of upstream intervention including: policies tackling the broader socio‐political determinants of health; policies combating NCDs linked to oral health; and some specific interventions (e.g., water fluoridation) focusing on oral health. The upstream approach to prevention remains highly relevant to public health policy and provides a guiding principle for future strategic action to promote oral health and tackle oral health inequalities. There is, however, a pressing need for more research to further develop the evidence base for upstream interventions using appropriate evaluation methods.

## Funding

The authors would like to thank the Borrow Foundation for their financial support to undertake this review.

## Conflicts of Interest

The authors declare no conflicts of interest.

## Supporting information


**Figure S1:** Empirical studies by year of publication (*N* = 74).

**Figure S2:** Empirical studies by region of origin (*N* = 74).

**Table S1:** Data extraction table for empirical studies.

**Table S2:** Data extraction table for systematic reviews.

## Data Availability

Data sharing not applicable to this article as no datasets were generated or analysed during the current study.
