# Supplementary figures and images for: Upstream Interventions to Promote Oral Health and Reduce Oral Health Inequalities: A Scoping Review
Source: Community Dent Oral Epidemiol. 2025 Dec 29;54(2):146–62. doi: 10.1111/cdoe.70049 (PMC13000981; doi:10.1111/cdoe.70049)

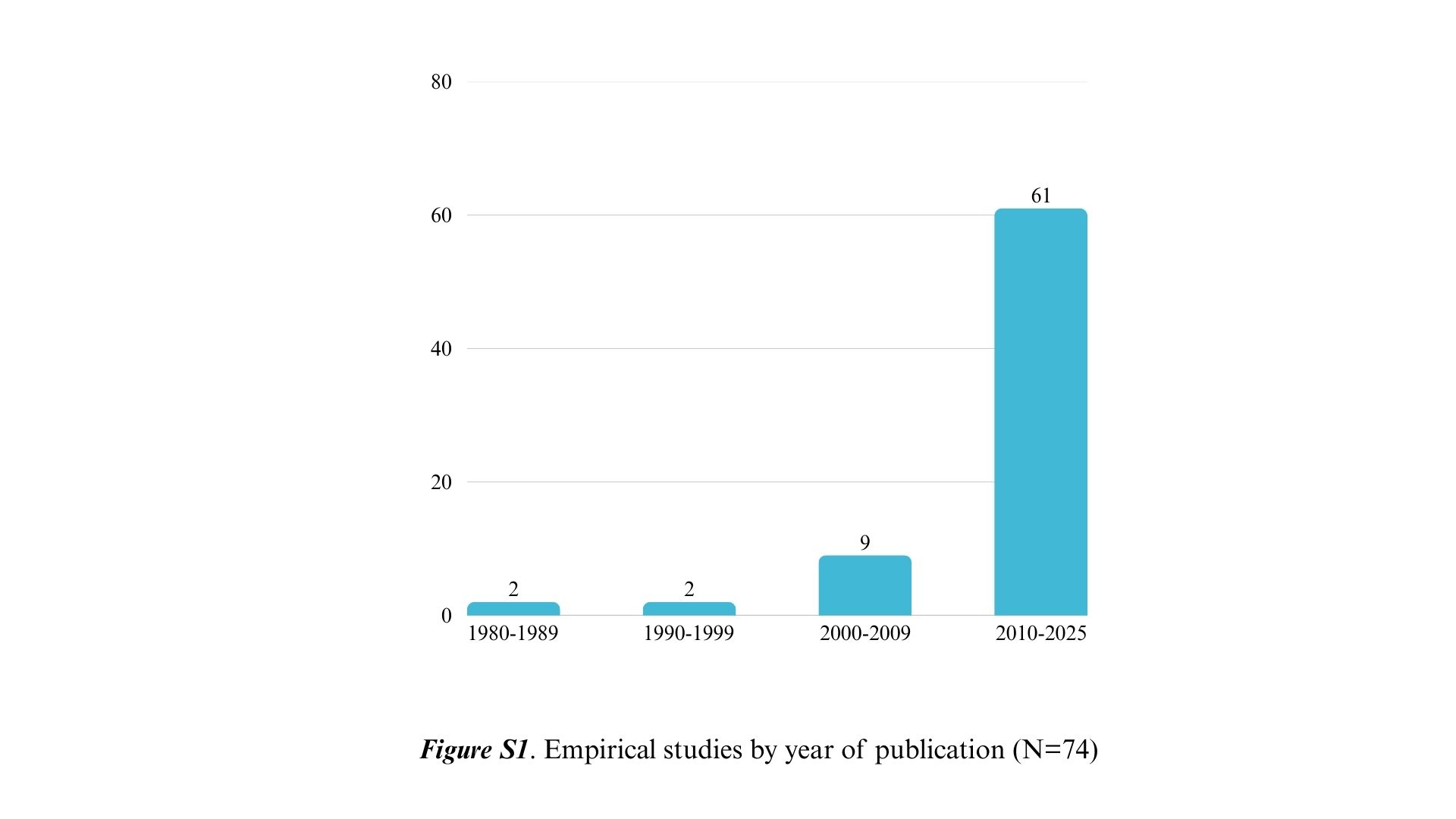

Supplement: Supplementary file 1 — Figure S1: Empirical studies by year of publication (N = 74). [file CDOE-54-146-s003.jpg]

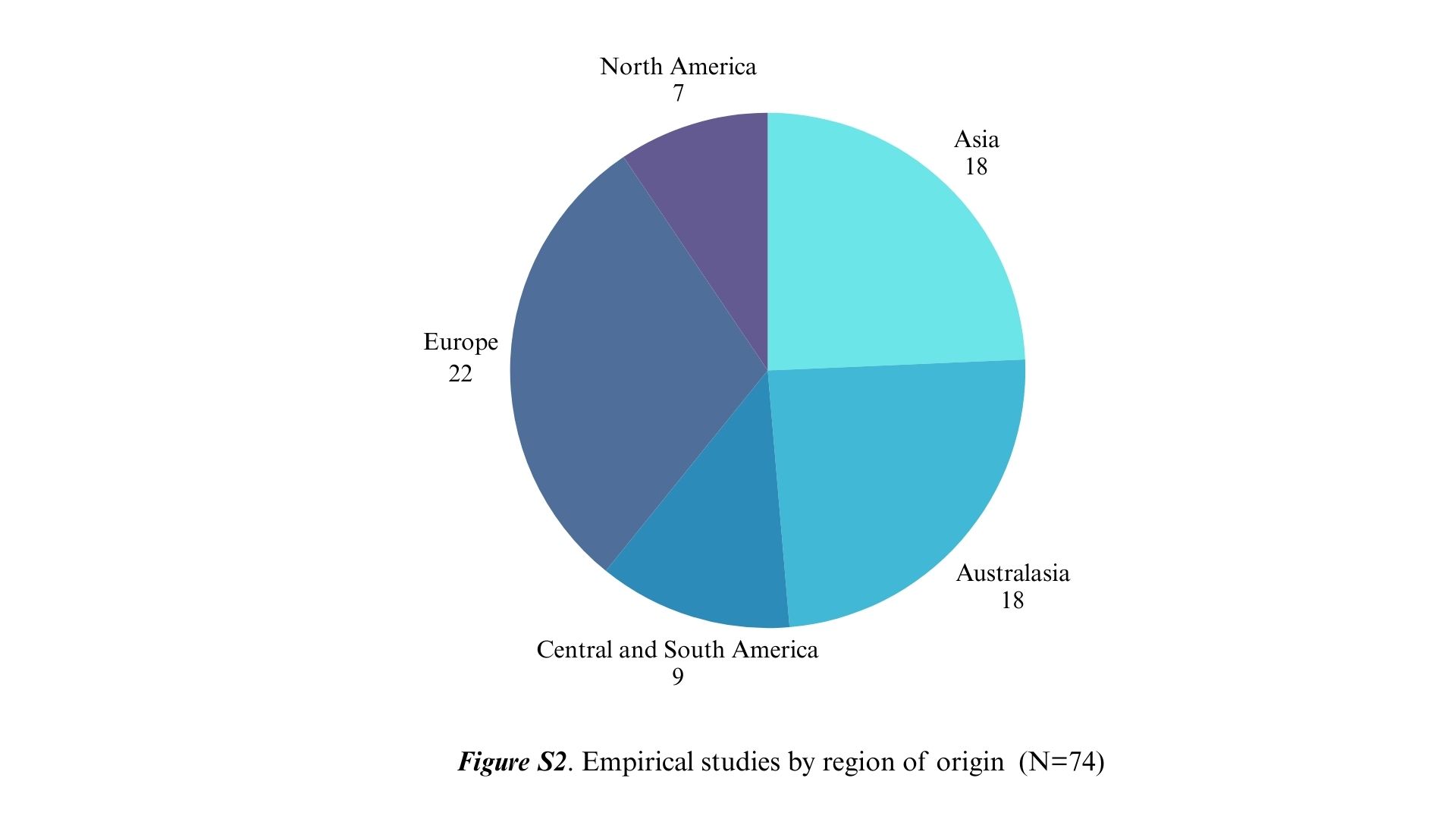

Supplement: Supplementary file 2 — Figure S2: Empirical studies by region of origin (N = 74). [file CDOE-54-146-s002.jpg]
