# Supplementary material for: Upstream Interventions to Promote Oral Health and Reduce Oral Health Inequalities: A Scoping Review
Source: Community Dent Oral Epidemiol. 2025 Dec 29;54(2):146–62. doi: 10.1111/cdoe.70049 (PMC13000981; doi:10.1111/cdoe.70049)
Supplement: Supplementary file 3 — Table S1: Data extraction table for empirical studies. [file CDOE-54-146-s001.docx]

# **Table S1. Data extraction table for empirical studies**

**Improved access to healthcare (N=5)**

| **Authors (year)**  **Title** | **Country of origin (published language)** | **Methods:**  **Type of study, study population, and sample size** | **Nature of upstream intervention** | **Outcome measures** | **Main findings** | **Conclusion** | **Strengths (S) and Weaknesses (W)** |
| --- | --- | --- | --- | --- | --- | --- | --- |
| Da Cunha et al., (2019)  Mortality from oral and oropharyngeal cancer in Brazil: impact of the National Oral Health Policy | Brazil  (published in English and Portuguese) | Ecological and analytical study    Mortality data from oral and oropharyngeal cancer obtained from the Mortality Information System, managed by Brazilian Ministry of Health (n = 61,190)    Deaths of individuals aged 15 years or older caused by malignant neoplasms of the oral  cavity and oropharynx. | Implementation of National Oral Health Policy (2004)    Key elements of the policy included:   - Restructuring of public dental services - Increasing access to dental services using oral health teams (OHT) - Increasing access to dental services with the restructuring of specialized dental care centers (SDC).   Between 2000-2013 | Mortality rate: Number of deaths from oral and oropharyngeal cancer per 100 000 inhabitants  Standardised by sex and age group, using world population data in 2010 | Mortality rate due to oral and oropharyngeal cancer was 3.59 times higher in men than in women.  Overall mortality rates by oral / oropharyngeal cancer were significantly associated with:   - The “average number of years of study” in both sexes (men: β = - 0.042; p=0.00; women: β = - 0.037; p= 0.01) - The “proportion of unemployed people with 16 years or more” in both sexes (men: β = 0.029; p=0.00; women: β = 0.016; p=0.01)   No significant association between mortality rates and “Gini index of the household per capita” and proportion of smokers aged 18 or more irrespective of sex.    Coverage of primary dental care (b= -0.096; p=0.05) and number of SDC’s (b=-0.025; p=0.01) was significantly associated with a reduction in mortality rates in males only. | This study found that the expansion of the coverage of primary dental care and the number of specialized dental care centers were associated with the reduction of mortality rates due to oral and oropharyngeal cancer in Brazil. | **S:**  Data considered reliable as it was obtained from official sources, validated by the Brazilian Ministry of Health    **W:**  Secondary data analysis, therefore “no possibility of ensuring the quality control of variables”.    The study recognizes a multitude of complex factors associated with cancer.  Multicollinearity among independent variables makes unreliable to do multivariate analysis.  Ecological study, therefore unable to assess causality relationship. |
| Ikenwilo (2013)  A difference-in-differences analysis of the effect of free dental check-ups in Scotland | Scotland  (English) | Difference-in-differences analysis (DID)    British Household Panel Survey (BHPS), N = 117,761 individuals (20,816 reside in Scotland and 96,945 reside in rest of the UK) | Free dental checkup on NHS    Started in April 2006 | Uptake of dental checkup | Prior to 2006 (when the free checkup was introduced), only a small proportion of Scottish population had dental check compared to the rest of the UK.    But this trend was reversed after 2006. 0.37 percentage point increase in NHS dental check-ups in Scotland over the study period (2001-2008), and a corresponding 6.27  percentage points reduction in dental check-ups in the rest of the  UK.   The increase in dental check-ups after the policy was larger in Scotland, an increase of 1.75 percentage points compared to the rest of the UK with an increase of 0.78 percentage points only.    Statistics suggested very modest advantages in switching from private and/or no dental check-up to NHS dental check-up in Scotland, compared to the rest of the UK.    Significant variations in NHS dental check-ups were related to variations in factors such as exemption status, age,  household income, employment status, health status, as well as initial conditions. | Results showed that there was a 3 to 4 percent increase in NHS dental check-up in Scotland, compared to the rest of the UK. Results suggested that a removal of financial barrier to dental check-ups lead to a modest increase in utilisation. | **S:**  DID analysis, this type of analysis has been widely used in the analysis of introduction of policy on specific outcomes.    Results weighted for attrition.    **W:**  Self-reported data subject to recall bias among respondents    Limited data after policy change 2005-2008 |
| Lyu et al., (2020)  Effects of the Recent Medicaid Expansions on Dental Preventive Services and Treatments | USA  (English) | Difference in difference (DID) study design    Restricted data from the 2011 -2016 Medical Expenditure Panel Survey (MEPS), N= 21000 newly eligible for Medicaid | Medicaid dental care expansion - increasing income eligibility and providing a given set of dental benefits to the new eligible benefactors.  Started in 2014 | Utilization of all dental services (preventive visits and treatment visits) | *Effects of Expansion with Extensive Dental Coverage:*   - Increased the likelihood of preventive dental visits by nearly 5 percentage points each year after the expansion.      - Likelihood of visits for major dental treatments increased by 4–5 percentage points in 2014–2015 but the effect was smaller by half and statistically insignificant in 2016, possibly reflecting greater demand for previously untreated dental problems shortly after gaining coverage.   *Effects of Expansion with Limited Dental Coverage*     - The likelihood of preventive visits increased in 2014 and 2015 by about 7 and 8 percentage-points, respectively.      - The likelihood of visits for major treatments increased by 3–5 percentage points across all three years after expansion which represented nearly 47%increase relative to pre-expansion rate. | Medicaid expansions with dental coverage beyond emergency-only services  increased access of the newly eligible low-income adults to dental treatments and preventive services | **S:**  Dental services were separated into preventive care and  treatments, using national data.    Difference-in-difference analysis to address confounding factors.      **W:**  Measure of dental coverage captured both the number of covered procedures and caps on spending, which could make a difference on the service use assessments. |
| Raittio et al., (2014)    Dental attendance among adult Finns after a major oral health care reform | Finland  (English) | Cross sectional postal surveys (repeated)    3 consecutive surveys on the use of oral health care services and perceived oral health.    The study population was Finnish adults born in 1970 or earlier, total N=7553  2001 (n=2837) *pre-reform*  2004 (n=2420) *between reforms*  2007 (n=2296) *after reforms* | Oral health care reform (OHCR) between 2001-2002:  Access to subsidised or free public dental services for the entire population and state reimbursements for private dental costs    Further legislative reform 2005:  Guaranteed access to care within 6/12 and standardised assessment for care need | - Used of oral healthcare services (during the past 12 months/number of visits)      - Perceived need for oral healthcare      - Perceived availability of public dental health services | Unadjusted:  Statistically significant increase in the use of oral health care services among people with low or medium level education since implementation of health reforms 2001-2007  low education= 45% to 51% (p:<0.05), medium education= 58% to 64% (p:<0.01)    Adjusted:  There was no change in the association of household income with the utilisation of oral health care services after the reformation of the oral health care.  Level of education was not associated with the use of oral care services. | The use of oral health care services rose, and age did not seem to be a barrier to the use of oral health care services after the reform.  No change in the association of household income with the use of oral health care services was seen after the OHCR. | **S:**  Representative sample    Good response rates- ranged from 60-70%. Response rates for the survey were 70% in 2001, 65% in 2004, and 60% in 2007.      **W:**  Unable to infer causality due to study design.    Unable to distinguish between general time trend and impact of OHCR |
| Raittio et al., (2015)  Income-related inequality and inequity in the use of dental services in Finland after a major subsidization reform | Finland  (English) | Cross-sectional study    Data collected from National postal surveys concerning the use of dental services and perceived oral health. The participants were individuals born in 1970 or earlier.    Before Oral Health Care Reforms (OHCR) in 2001- (n= 1907)  After OHCR in 2004 -(n=1629) and in 2007- (n=1509) | Oral Health Care Reforms (OHCR) in 2001-2002.  Introduction of Public Dental Service (PDS) for everyone and reimbursement for private dental treatment from NHI | Income related inequalities and inequities in the use of dental service after OHCR | Pro-rich inequality and inequity in the use of dental service narrowed shortly after OHCR in 2001. But this widened again from 2004 to 2007 to a similar level pre-OHCR phase.   There was a pro-poor inequality in the use of PDS and a pro-rich inequality in the use of private dental service.    There are several reasons for the temporary pro-poor change post-OHCR. There was a backlog of unmet needs in low-income groups and service use was increased after the introduction of PDS. But the increase was not permanent and mostly associated with higher level of education in 2007 as compared to 2001.    There was a pro-rich inequality in perceived oral health, need for care and reported toothache.    The used of PDS became more need-based and hence pro-poor after OHCR. | Income-related inequality and inequity in the used of dental services were narrowed only temporarily after the reform. | **S:**   Representative sample    High response rate, ranged from 60 – 70 %    **W:**  Short follow-up time  Causal effect of OHCR not determined.  Differences in quantities of service use were measured and the study was not able to control for differences in the quality of visits.  Bias due to self-reported nature of data. |

**Health Insurance Changes N = 9**

| **Authors (year)**  **Title** | **Country of origin (published language)** | **Methods:**  **Type of study, study population, and sample size** | **Nature of upstream intervention** | **Outcome measures** | **Main findings** | **Conclusion** | **Strengths (S) and Weaknesses (W)** |
| --- | --- | --- | --- | --- | --- | --- | --- |
| Choi, (2020)  Has the Health Insurance Coverage of Scaling Contributed Positively to Periodontal Health in Korea? | South Korea  (English) | Cross sectional    Korea National Health and Nutrition  Examination Survey (KNHANES)    Pre post coverage data from 2010 and 2012 (excluding 2011)    Post coverage period data from 2016 to 2018 (three years after  the implementation of the policy)    Only participants aged 20 years or older were eligible for the scaling coverage.  Only the  participants who responded to all variables utilized in the analysis were included  n= 10,802 before the implementation of scaling coverage; and n = 12,245 people after scaling coverage | Korean scaling insurance introduced in 2013 for adults aged 20 years or older – covering 1 scaling per year | Clinical data collected by dentist at mobile examination center.    Periodontal status determined using CPITN code.    Periodontal status categorized as:   - Healthy periodontal tissue: score 0 for all sextants.      - Need for scaling: At least one sextant with a score of 2,3, or 4.      - Prescence of periodontal disease: At least one sextant with a score of 3 or 4. | There was an increase of the rate of healthy periodontal tissue from 34 % pre-coverage of the policy to 39.1 % after the coverage, showing an increase of 4.9 % (p<0.001)  After scaling intervention % of healthy periodontal tissue was higher amongst women (vs men), those aged 20-64 (vs those aged 65 or more), urban region (vs rural region), increasing household income, ex/ non-smokers (vs smokers), had oral examination in the past year (vs no oral examination in the past year) and those who brushed 2 or more times/day (vs <2 times/day) (p < 0.001)  Increased prevalence of periodontal disease: 23.4% vs 30.6% - post policy vs pre policy.    Scaling policy associated with an increase in periodontal disease (0R=1.50; p <0.001) - post-policy vs pre-policy. Higher increase in periodontal disease significantly associated with low SES, those living in rural communities, males, smokers, and older adults (p<0.001) | The implementation of dental scaling coverage led to an increase in percentage of adults with healthy periodontal tissues. There was a decrease of adults in need for scaling and an increase in the percentage of adults with periodontal disease. | **S:**  Periodontal disease measured using a clinical index.    **W:**  Observational study, thus this study was not able to establish a causal relationship.    Limitations in practical policy implementation |
| Choi & Ma, (2020)  Changes in oral health indicators due to implementation of the National Health Insurance Services coverage for first molar dental sealant for children and adolescents in South Korea | South Korea  (English) | Cross-sectional    Korea National Health and Nutrition  Examination Survey (KNHANES). The participants involved in this study were individuals of age 11-20 years.    2 period:  Fourth period (2007-2009) - pre policy data, n = 3,092    Sixth period (2013-2015)- post policy data, n = 2,591 | Policy changes to Korean National Health Insurance Services (NHIS)    2009: Dental sealant included in NHIS for first molars for children aged 6-14 years   2012: Fissure sealant of second molars included in NHIS    2013: Age of insurance coverage extended to under 18 | Outcomes:     - Proportion of individuals with first molar sealants - DMF first molar permanent teeth - Single crowns | Post policy vs pre policy:    Number of sealant restorations increased by 7.7 % (p<0.001)    Number of permanent teeth with sealant per capita increased from approx. 0.4 to 0.8 (p<0.001)    Proportion of participants with DMF permanent teeth decreased by 9.1% (p<0.001)    The rate of single-crown holders decreased by 2.7% (p>0.05)    Number of sealants increased with age and household income (p<0.001)    After policy implementation: Low SES (Mother education level less than high school vs more than college) was significantly associated with a lower sealant rate (OR=0.393; P<0.05) | Sealant treatment for the first molar in children and adolescents aged 11– 20 years was significantly higher in reimbursement policy beneficiaries compared to non-beneficiaries, and caries was significantly lower. | **W:**  Observational study - unable to establish a causal relationship.    Second molar not included in analysis – resulting in under estimation of point estimates. |
| Jang et al., (2017)  Utilization of Preventive Dental Services Before and After Health Insurance Covered Dental Scaling in Korea: 2009 to 2014 Community Health Survey | South Korea  (English) | Time series analysis of secondary data    Participants from 253 survey areas nationwide from the Community Health Survey in Korea (2009-2014), n = 3, 175, 584    Conducted annually by the Korean Center for Disease Control and Prevention for 3 months (Aug-October) | Health Insurance modified to include preventative dentistry.    In 2013, annual dental scaling for those aged 20 years and over was included in the policy.    Patient cost of dental scaling was reduced from $50 to $10. | Weighted proportion of participants who underwent dental scaling (scaling rate)  Dental scaling defined as removal of dental plaque, dental calculus, nicotine and staining from the tooth surface. | Annual scaling rates increased for all age groups (p<0.001)   Annual scaling rates increased for all occupations (p<0.001). However, scaling rates higher in those with specialized jobs/ office work compared to those who work in agriculture/technical workers and laborer's/ unemployed.    Annual scaling rates increased for all education levels (p<0.001).  Clear social gradient observed. Those with college/ higher education reported higher scaling rates compared to those with lower education levels.    Annual scaling rates increased for all income groups (p<0.001). Positive relationship between annual income and scaling rates – clear social gradient evident | Since 2013, when coverage for dental scaling was introduced, the proportion of adults in Korea who underwent dental scaling drastically increased, up to 4.1%p (men, 3.7%p; women, 4.5%p).  The national health insurance had substantially improved the accessibility of preventive dentistry services, but the use rates of preventive care among low socioeconomic classes remained low. | **S:**   National survey data used.   Analyses carried out using weighted data.    **W:**  Different participants were used annually to collect data.  Recall bias as respondents were expected to remember their dental scaling in the previous year.    Bias associated with “survey environment and situation” as the data were collected through one-on-one interviews. |
| (Kim & Kim, 2021)  Trends in Self-Rated Poor Oral Health Among all Age  Populations in Korea from 2007 to 2015: Monitoring Expansion of Dental Insurance | South Korea  (English) | Cross sectional secondary data analysis    Korean National Health and Nutrition Examination Survey (KNHANES) (2007-2015) waves IV, V and VI. This survey categorised their respondents by age: 0-19 years (children/ adolescents), 20−44 years (young adults), 45−64 years (middle-aged adults), and 65 years or older (older adults).    n= 20,199 | Expansion of health insurance coverage to include dental care:     - Pit and fissure sealing since 2009. - Denture treatment since 2012. - Dental scaling since 2013. | Questionnaire data    Self-rated poor oral health (SRPOH) - defined as a rating of bad/ very bad vs very good, good and average. | Age-sex standardized prevalence rates showed a decrease of SRPOH amongst all age groups over time. Marked change between KHANES-V (2010-2012) and KHANES-VI (2013-2015) found.    Prevalence of SRPOH in the entire sample decreased by 11% from 2007-2009 (25%) to 2013-2015 (14%).    The prevalence of SRPOH decreases were significant amongst those aged 0-19 years (p=0.003) and 20-44 years (p=0.038). No significant findings in those aged 45 and over.    Results of logistic regression:    Significant association between SRPOH and sex; income; and education between (2007-2015).    In the overall sample after adjusting for covariates:    In 2007-2009: OR=1.94 (worse self–rated oral health vs good/ moderate) (p<0.05; 95CI: 1.65-2.27)    In 2010-2012:  OR=2.03 (worse self–rated oral health vs good/ moderate) (p<0.05; 95CI: 1.67-2.47)    In 2013-2015:  OR=1.96 (worse self–rated oral health vs good/ moderate) (p<0.05; 95CI: 1.96-2.42) | Self-rated poor oral health improved among younger people in Korea. First, younger generations (aged 0−19 and 20− 44 years) significantly improved their SRPOH. However, no statistically  significant improvement was found in middle-aged or older adults.  The gender gap in the prevalence increased with age and persisted over time. | **S:**  Nationally representative data.    Age-sex standardised prevalence data.  Interactions terms were considered for comparison.    Different age groups were analysed.  **W:**  Information bias according to “age and education level”.  Potential bias from unobserved socioeconomic status (SES) variables as covariates. |
| Kim & Kawachi, (2020)    Did the Expansion of Insurance Coverage for Oral Health Reduce Self-Reported Oral Health Inequalities in Korea? Results of Repeated Cross-Sectional Analysis, 2007–2015 | South Korea  (English) | Cross sectional    Data from Korea National Health and Nutrition Examination Survey (KNHANES) (2007-2015).  This survey categorised their respondents by age: 0-19 years (children/ adolescents), 20−44 years (young adults), 45−64 years (middle-aged adults), and 65 years or older (older adults).  N = 68,431 | 2012 National health insurance expansion of dental care coverage | Income based oral health inequalities.    Unmet dental needs due to cost | Income based inequalities in unmet dental needs persisted in older women but after the 2012 expansion the inequality in unmet dental needs decreased for young and middle aged (P for trend ≤ .001) | Findings suggest that, although health insurance expansion improved access to dental care and may have contributed to an absolute improvement in self-reported oral health for most groups, the relative income gradient in oral health remained resistant to change. | **S:**  Nationally representative data, so the results can be generalized to the entire population in South Korea  Representative sample across all age groups from children to older adults.  Identify population level trends from the long-term repeated cross-sectional data.  **W:**  Did not consider income issues in major cities where purchasing power were different to rural places.    Health insurance expansion was for scaling and denture services, but outcomes did not distinguish types of dental treatment. |
| Kim & Kawachi, (2021)  Insurance coverage expansion and inequalities in unmet oral healthcare needs in Korea: Repeated cross-sectional analysis, 2007-2015 | South Korea  (English) | Cross sectional data analysis    KNHANES survey data between 2007-2015    N = 21343  Aged:20 - 65+ years | Expansion of Korean health insurance policies (2012) to include:    A. Dental scaling for those aged 19 years and above    Provision of dentures and implants for those aged 65 years and above | Reduction of income-based inequalities in unmet dental needs due to cost | Prevalence of unmet dental need was higher in low-income groups across all age groups.    After the 2012 insurance expansion, the absolute and relative inequality in unmet dental need decreased for all age groups except older women (p= <0.001). | Income-based inequalities in unmet dental needs persisted among older women in Korea despite the insurance expansion. By contrast, after the 2012 insurance expansion, the absolute and relative inequality in unmet dental needs decreased for young and middle-aged as well as older adults. | **S:**  Used nationally representative data, KNHANES, thus the results are generalised to the entire population in South Korea.  Ensured comparability and generalizability through internal validity and external validity, respectively.  Prevalence  rate was standardized based on four income strata by age group and gender in each wave.  The interaction term was considered for the outcome trends among the survey waves.  **W:**  Unable to establish a causal relationship.    Only income-based health inequalities considered resulting in possible under/ over estimation of results.    Insurance expansion was for scaling/ denture services however, the outcome variable did not distinguish type of dental treatment. |
| Shin et al., (2021)  Dental Expenditure by Household Income in Korea  over the Period 2008–2017: A Review of the National Dental  Insurance Reform | South Korea  (English) | Cross sectional study  Korea Health Panel (KHP) survey, data from 10 years period (2008-2017).  The study used national representative households and individuals’ data. Participants divided into two groups: 65 years and less (non-elderly) vs those aged 65+ (elderly)    n = 7681 households; n = 16,493 household members, and n = 173,863 cases of dental care | Dental reform to Korean National Health Insurance.    Reformed between 2008-2017 | Dental expenditure analyzed by total dental expenditure and out-of-pocket (OOP) expenditure.    Dental service utilization – 13 categories of dental treatment | Total health care expenses and OOP dental expenditure (2011-2017) and OOP expenses for dental treatment (2008-2017) tended to increase over time.    Elderly group:  Total dental expenditure increased but OOP did not change significantly.    After adjustment for confounding factors OOP proportion to total expenditures decreased after dental insurance reform (2013-2017) compared to the previous period (2008-2012).    Implant services showed the largest difference in data during 2013-2017 vs 2008-2012 in elderly and non-elderly groups.    Income-related dental inequalities were seen in both elderly and non-elderly groups. In both 2008-2012 and 2013-2017 high income groups showed higher OCP expenditure however “pro-rich” inequalities of dental service utilization decreased in 2013-2017 compared to 2008-2012    Elderly group: In 2008-2012, conservative, periodontal and endodontic services were more likely to be carried out in high income groups, but after implementation of the dental reform period (2013-2017), this “tendency disappeared” except from endodontic services. | The dental health insurance reform in Korea contributed to lower the ratio of OOP and total dental expenditure per episode in the elderly. This dental reform also improved the inequality of dental expenses. | **S:**  KHP data used – considered “crucial data” for national healthcare policy.    **W:**  KHP data collected via one-to-one interview – recall bias on actual dental treatment received by participant.    Some possible problems with sample representativeness  due to panel attrition. |
| Shin et al., (2020)  Did the extended coverage policy contribute to alleviating socioeconomic inequality in untreated dental caries of both children and adolescents in South Korea? | South Korea  (English) | Cross-sectional survey    Korean National Health and Nutrition Examination Survey (KNHANES)    Comparative data: Fourth wave (2007-2009) vs sixth wave (2013-2015).  The study population was children aged 6 -18 years. Children was divided into 2 groups: 6-11 years and 12-18 years.    n = 7410 participants (4353 in the fourth  and 2915 in the sixth wave) | Change to public health policy.    Dental sealants covered by the National Health Insurance Service (NHIS) since 2009. Only the first molars of children aged 6 –14-year-old covered. In 2013 coverage extended to second molars for children up to 18 years old. | Oral health examination by calibrated dentists    Outcome variable: caries and sealant experience based on WHO criteria. | Untreated decay:   At the fourth wave there was significant SES inequalities in the prevalence of untreated decay across all 4 income groups for the 6-11 age group (p=0.001) and 12-18 age group (p< 0.001)    However, policy change resulted in the reduction of untreated decay across all income levels (in the 6-11 age group) except in the highest income level (prevalence rate of untreated caries in highest income group: 5.2 (2007-09) vs 6.8 (2013-15). But this result was insignificant (p=0.152)    12-18 years: Policy change resulted in the reduction of untreated decay across all income levels, but there were significant differences amongst income groups (p=0.006).  Dental sealants:  At the fourth wave there were significant SES inequalities in the prevalence of dental sealants across all 4 income groups for the 6-11 age group (p=0.008) and 12-18 age group (p< 0.001)    However, policy changes resulted in the increase of dental sealants across all income levels, but significant differences remained in the 12-18 age group across all income levels.   Those aged 12-18:    Adjusted analysis showed that those in the lowest income group vs the highest income group, were significantly more likely to have a higher prevalence ratio of untreated decay (2013-2015: PR=1.67; 95%CI: 1.28-2.18; P<0.001; 2007-2009: PR=1.44; 95%CI 1.22-1.71; P<0.001).    Adjusted analysis showed that those in the lowest income group vs the highest income group, were significantly less likely to have a lower prevalence ratio of dental sealants (2013-2015: PR=0.70; 95%CI: 0.58-0.85 P<0.001; 2007-2009: PR=0.57; 95%CI 0.47-0.70; P<0.001).    Similar results were also seen in the 6-11 age group. | This study found that socioeconomic inequality in un-treated dental caries and sealant treatment was alleviated for children by an expansion of NHIS coverage in Korea. | **S:**  Nationally representative data  **W:**   The NHIS changed their policy too often in relation to dental sealant treatment, therefore policy was undergoing a transitional stage – which might impact the study results. |
| Wang, Wang & Hung, (2018)  Universal health insurance, health inequality and oral cancer in Taiwan | Taiwan  (English) | Interventional study    N = 5736  Patients with oral SCC Age: 20-79 years    Data collected from Taiwan Cancer Registry:  Before NHI 1990-1994  After NHI 1998-2007 | National Health Insurance (NHI) – universal coverage for everyone | Life expectancy    Expected Years of Life Lost (EYLL) | The introduction of NHI increased the number of diagnosed cases in different income groups.    Life expectancy of all oral cancer patients improved after NHI was introduced.    For female patients, those with high-income had the smallest loss of life expectancy. EYLL for low-income female patient was greater than those for high-income females.    For male patients, life expectancy was almost same irrespective of income group, but it improved for all income groups after the introduction of NHI.    Low-income male patients had the highest EYLL post NHI. | Universal coverage alone did not reduce health inequality across different income groups for oral cancer. | **S:**  Nationally representative data derived from cancer registry.    Survival analysis extrapolation |

**Health Marketing Campaign N=1**

| **Authors (year)**  **Title** | **Country of origin (published language)** | **Methods:**  **Type of study, study population, and sample size** | **Nature of upstream intervention** | **Outcome measures** | **Main findings** | **Conclusion** | **Strengths (S) and Weaknesses (W)** |
| --- | --- | --- | --- | --- | --- | --- | --- |
| Bradley et al., (2020)  Impact of a health marketing campaign on sugars intake by children aged 5–11 years and parental views on reducing children’s consumption. | England  (English) | Longitudinal survey with 5 time points    Families recruited from PHE *Change4Life* database.    n=873  Age=5-11 years    Information on dietary intake collected at:    Baseline: 2/3 Jan 2016  Peak campaign: 30/31 Jan 2016  Post campaign: 27/28 Feb 2016  10 months post campaign: 20/21 Nov 2016  12months post campaign: 29/30Dec 2016 | Health marketing campaign: *Change4Life* Sugar Smart Campaign 2016.    6-week campaign was running on TV, billboard, and digital advertising campaign. Smart App also available to download. | -Total sugars (g/day)   -NME sugars (g/day) (as a measure of free sugar intake) assessed using the NDNS method.  -Percent contribution of sugar and free sugar to energy intake  -Change in total intake of energy/ fat.  -Dietary information collected by online self-completion 24-hour dietary recall system “Intake 24”.    Qualitative outcomes also considered. Information collected via semi-structured interviews. Topics explored included:   -Understanding the campaign messages  -Understanding sugar and its impact on health  -Individual, family and social barriers to reducing sugar intake | -Significant decrease in % of energy from total sugars across time points, compared to baseline. Long term sustainability of intervention questioned.  -Significant decrease in amount of total sugars consumed at peak of campaign (reduction of 6.2 g/day), immediately post campaign (reduction of 5.5g/day) and 10 months post campaign (reduction 3.5g/day)    -% of energy from NMES significantly decreased across all time points except at 12 months post campaign.   -% of energy from fats increased significantly across all time points.    Barriers to healthy eating included: “pudding culture” at schools; misleading food marketing; peer pressure; confusion over good/ bad sugar; treat culture; busy parental lifestyles. | The study showed that the health marketing campaign was successful in reducing the mean intake of total sugars by approximately 2% of total energy intake in a group of children whose families had shown an interest in previous *Change4Life* campaigns, however reductions were not sustained at the 12-month follow up. | **S:**  First study to measure impact of health marketing aimed at reducing sugar consumption.    **W:**  Selection bias    Dietary information recorded at the weekend only – more likely to consume sugar at this time.    Recall bias in relation to dietary intake.    Campaign was run in the New Year, therefore seasonal attitudes to dietary changes could not be ruled out. |

**Water Fluoridation N = 32**

| **Authors (year)**  **Title** | **Country of origin (published language)** | **Methods:**  **Type of study, study population, and sample size** | **Nature of upstream intervention** | **Outcome measures** | **Main findings** | **Conclusion** | **Strengths (S) and Weaknesses (W)** |
| --- | --- | --- | --- | --- | --- | --- | --- |
| Armfield, (2010)  Community Effectiveness of Public Water Fluoridation in Reducing Children’s Dental Disease | Australia  (English) | Cross sectional community level survey    Children aged 5 – 15 years who attended a regular dental visit at an Australian state or territory School Dental Service in 2002    N = 111 576 | Water fluoridation    Fluoride levels categorized as:   Fluoridated water= ≥ 0.7 ppm    Non-fluoridated water= <0.3 ppm    (0.3-0.69 ppm excluded from analyses due to small numbers) | Caries prevalence defined as: One or more teeth with decay into dentine, teeth filled because of decay or teeth extracted because of decay.   Disease experience defined as: count of the total number of decayed, missing and filled teeth (dmft/ DMFT) | Across all age groups (5-15 years), in both the permanent and deciduous dentition, those lived in a high fluoride area (≥0.7 ppm vs <0.3 ppm) had significantly less caries prevalence and disease experience compared to those in non-fluoridated areas.    In the deciduous dentition, caries prevalence was between 7.4-31.1% higher in non-fluoridated areas vs fluoridated areas    In the permanent dentition caries prevalence (non-fluoridated vs fluoridated) was 4.7- 29.4% higher    Adjusted analysis showed that those living in non-fluoridated area vs fluoridated area had 1.38 higher odds of having at least 1 dmft tooth in the deciduous dentition (p<0.001; 95%CI 1.29-1.39)    Adjusted analysis showed that those living in non-fluoridated area vs fluoridated area had 1.24 higher odds of having at least 1 DMFT tooth in the permanent dentition (p<0.001; 95%CI 1.21-1.28) | Water fluoridation was found to be related to significantly reduced caries experience in majority of area health service regions. | **S:**  Large population sample.    Comprehensive study.    **W:**  Community level study therefore lack of individual data on fluoride exposure e.g., no information on residential mobility (moving from fluoride area to non-fluoride area and vice versa) |
| Armfield, (2005)  Public water fluoridation and dental health  in New South Wales | Australia  (English) | Cross sectional study    Children aged 5 – 14 years who attending the School Dental Service in NSW, Australia in 2000    N= 248,955 children | Water fluoridation with the categorization as below:    Negligible or 0 ppm (less than 0.3 ppm F)    Suboptimal or 0.5 ppm (0.3-0.7 ppm F)    Optimal or 1 ppm (> 0.7ppm) | DMFT/ dmft index | Community effectiveness of water fluoridation: Strong and consistent pattern of results indicating that children residing in areas of >0.7ppm fluoride had lower caries prevalence and experience.    Amongst 5–6-year-olds dmft was significantly higher in non-fluoridated areas vs fluoridated areas in 6/8 health regions of Australia (p<0.001)    Amongst 11–12-year-olds DMFT was significantly higher in non-fluoridated areas vs fluoridated areas in 6/10 health regions of Australia (p<0.05)    Amongst 5–6-year-olds clear social gradient evident across those living in non-fluoridated and fluoridated areas in relation to dmft    Across all SES categories significant reduction in dmft in fluoridated water vs non fluoridated water (p<0.001).    Percentage of the difference in dmft between non-fluoridated and fluoridated areas ranged from 47.6%-75%    In permanent dentition (those aged 11-12 years): significant association between disadvantage and caries experience in non-fluoridated and fluoridated areas.    However, significantly higher DMFT found in 11-12 years old from the most and least disadvantaged backgrounds (non-fluoridated areas vs fluoridated areas)    Indigenous children:  Within each age group, Indigenous children (vs non-Indigenous children), had greater caries experience.    Both Indigenous and non-Indigenous children had reduced caries experience in fluoridated vs non-fluoridated areas. | Water fluoridation was found to be related to significantly reduced caries experience in the majority of Area Health Service regions (AHSs) regions and gave benefit to all socio-economic strata of the community. | **S**:  Large sample size of data, almost one-quarter of a million children in New South Wales  **W:**  No measure of individual exposure to water fluoridation    Participants residing in non-fluoridated areas might potentially exposed to fluoride via food/ beverages - “halo effect”.    Unadjusted analysis |
| Carmichael, Rugg-Gunn & Ferrell, (1989)  The relationship between fluoridation, social class and caries experience in 5-year-old children in Newcastle and Northumberland in 1987 | UK  (English) | Case-Control study    Children aged 5-year-old who lived in a fluoridated area of Newcastle, n = 457    Children aged 5-year-old lived in non-fluoridated area of Newcastle South Northumberland, n = 370 | Water fluoridation    Fluoridated area (1mg F/ litre) vs non-fluoridated area (<0.1 mg F/litre) | Children clinically examined by one examiner under standardised conditions.    Caries experience (dmft)    Caries at cavitation stage recorded | There was less caries prevalence in fluoridated areas vs non fluoridated areas across all SES groups.    Clear caries social gradient evident across all SES groups in fluoridated and non-fluoridated areas    Difference in mean dmft between social class 4 and 5 (low fluoride vs fluoride) = 2.6    Difference in mean dmft between social class 1 and 2 (low fluoride vs fluoride) =1.1  Comparing SES groups 4 and 5:  Children living in a non-fluoridated area compared to a fluoridated area more likely to: have toothache, (42% vs 27%); have GA extraction (32% vs 24%); have one or more extracted teeth (33% vs 19%); have 3 or more decayed teeth (53% vs 21%); dmft 5+ (21% vs 45%) and one or more filled teeth (17% vs 22%). | Fluoridation reduced caries prevalence for this age group, but did not eliminate social inequalities, social disadvantage/ social class background as the major factors which influence caries prevalence. | **S:**  Case-control study to compare caries prevalence between 5-year-ols children who lived in a fluoridated area and non-fluoridated area.  **W:**  Occupation used as measure of social class – not considered a representative measure of SES. |
| Cho et al., (2016)    The differences in healthcare utilization for dental caries based on the implementation of water fluoridation in South Korea | South Korea  (English) | Case control study    National Health  Insurance Service National Sample Cohort (2002-2013).  n = 472,250 patients aged 19 and above. | Community water fluoridation of 164 non-metropolitan areas | Utilization of dental healthcare services for caries    Frequency and cost of dental care visits as a measure dental expenditure | Average percentage of patients that visited outpatient dental services: 46.98% in water fluoridated areas vs 48.66% in non-fluoridated areas (p<0.0001)    Those more likely to attend dental services were female, younger, wealthier and had employee insurance (p<0.05).    Individuals in water fluoridated areas were less likely to utilize dental services compared to those living in non-fluoridated areas (p <0.001).    Average number of dental care visits and medical costs were significantly lower amongst those residing in water fluoridation areas (p<0.001).    Individuals living in water fluoridated areas had a lower risk of dental care visits (Higher risk of dental visits=0.949, 95% CI=0.928-0.971)    Period of water fluoridation had an inverse association with dental care expenditure. | Water fluoridation programs were associated with reducing dental health care visits. | **S:**  Nationally representative sample    First study to investigate relationship between water fluoridation and the whole nation of South Korea- most previous studies conducted at community level.    Dental visit / expenditure as outcome variables to assess dental utilization.    **W:**  No clinical outcomes considered.    Actual water fluoride concentration unknown – limited data.    No data on actual water consumption.    Non-payment items related to dental care not considered – results may be underestimated.    Regions that quit the water fluoridation programme not considered in the analysis. |
| Chondur et al. (2024)  Effects of community water fluoridation on child dental caries in remote Northern Territory, Australia: a difference-in-difference analysis | Australia  (English) | Difference in difference analysis  24 546 children (observations from 2008-2020) | Community water fluoridation (CWF) in remote areas measured pre and post 2014  Water fluoride concentrations in 2014:  ***Treatment group:***  (0.54mg/L)  ***Control group 1:***  Naturally occurring fluoride >0.5mg/L  (0.86mg/L)  ***Control group 2:***  Naturally occurring fluoride <0.5mg/L  (0.16mg/L) | Caries experience (dmft/DMFT) in children aged 1-17 years | Greater magnitude of caries decrease observed in children from the treatment group compared to both control group 1 and control group 2 post 2014.  ***Treatment group:*** Decline in the number of teeth affected by caries by an average of 0.28 (p=0.001)  Children in the treatment group experienced  significantly greater post-intervention declines in average dmft/DMFT in comparison to control group 2  7–10 years: 0.32 (p=0.051)  11–17 years: 0.40 (p=0.012) | Clear benefits of implementing CWF in remote areas where fluoride concentrations are below optimal levels. | **S:**  Large dataset  **W:**  Limitations to difference in difference analyses- unable to assess background trends affecting control and treatment groups  Unable to account for sociodemographic, dietary and behavioural factors or differential rates in water consumption across groups  Unable to assess dose response differences in caries rates in areas with optimal water fluoridation |
| Ciketic, Hayatbakhsh & Doran, (2010)  Drinking water fluoridation in South-East Queensland: a cost-effectiveness evaluation | Australia  (English) | Cost effectiveness analysis    South-East Queensland newborns population, n= 36,322 newborns | Cost effective analysis of fluoridation of drinking water supplies in Brisbane and South-East Queensland | Reduced cost of dental treatment    Years of life with dental caries as a disability (DALYS) | Fluoridation remained a “very cost-effective measure for reducing dental decay”.    Implementation of water fluoridation would result in:     - A mean cost saving of $665, 686,529 Australian dollars (95% CI: $973,573,625- $381,322,176)      - A total saving of $10, 437.43 (95% CI: 6.406.50-14,035.35) DALYS | Fluoridation was a cost-effective measure for reducing dental decay in Brisbane and South-East Queensland population. | **S:**   Analysis of costs and outcomes of fluoridation “well beyond” a previous study conducted in 2002. This study considered both DALYS and monetary costs  .  **W:**  The cost-effectiveness ratio was underestimated as data related to dmfs and not DMFS.    Cost of water fluoridation plant used in analyses likely to be overestimated.    Cost of one dental visit and a two-surface restoration considered in analysis – extensive visits/ dental costs not considered.    Disability weight used in analysis – most likely to be higher than other published weights resulting in over estimation of results. |
| Cronin et al., (2021)  A cost‑effectiveness analysis of community water fluoridation for schoolchildren | Ireland  (English) | Cost effectiveness analysis    Epidemiological data for children aged 5, 8, and 12-year-old school children from data collected in Fluoridation and Caring for Children’s Teeth (FACCT) study. | Community Water Fluoridation (CWF) | Dental caries prevented attributable to CWF.    Potential treatment savings associated with CWF.    Lifetime treatment cost of decay.  The incremental cost per decayed, missing, or filled tooth (d3vcmft/D3vcMFT)    Dental Fluorosis | Average caries levels with CWF:   - d3vcft for 5 and 8 years old 0.96 and1.69 respectively - D3vcFT and MT for 12 years old: 0.71 and 0.04 respectively    Average caries levels without CWF:   - d3vcft for 5 and 8 years old 1.72 and 3.20 respectively - D3vcFT and MT for 12 years old: 1.32and 0.07 respectively     **Annual treatment savings:** A total saving of €2.95 million to the health-payer, of which 71% related to direct treatment savings. The net cost of CWF (cost of CWF provision minus the expected treatment savings associated with dental caries  prevented) was estimated at €2.63 million in 2017.    The probabilistic sensitivity analysis reported the mean net cost of CWF per  D3vcft/ D3vcMFT  Prevented was- €72.35 (SD: €19.31, UI: -€ 42.57 to -€ 105.98) per 5-year-old child,  -€104.19 (SD: €20.54, U:-€ 71.75 to  -€ 139.12) per 8-year-old child, and  -€ 205.03 (SD: €43.84, UI:-€ 133.51 to-€277.16) per 12-year-old child.    Estimates of 8- and 12-year-old school children with lifetime exposure to CWF, an annual fluorosis treatment cost of €414 per affected child was required to negate the treatment savings attributable to CWF. | The benefits returned by CWF programme exceed the total cost of providing the intervention efforts for Irish schoolchildren. | **S:**  Representative and good number of sample size.    Sensitivity analysis confirmed the study results were sensitive to assumptions around CWF efficacy and lifetime cost of dental treatment.    **W:**  Sample included certain age of school children only.    The estimates around expected treatment savings associated with CWF considered conservative due to various reasons:     - The study did not include private dental services and was only confined to primary care. - Assumptions around equal cost for both single and multi-surface restorations, did not allow the possibility of treatments between designated treatment cycles. - Treatment cost did not incorporate the financial burden arising from absence from school and work due to dental disease. |
| Do et al., (2018)  Race- and Income-Related Inequalities in Oral Health in Australian Children by  Fluoridation Status | Australia  (English) | Cross sectional survey    National population-based survey of Australian children aged 5 to 14 years (2012-2014)    N = 21328 | Water fluoridation    Participants stratified by fluoridated water area (F level greater than 0.5mg/L) and non-fluoridated area (F level less than 0.5mg/ L) | DMFS/dmfs in both primary (age 5-10) and permanent dentition (age 9-14) | Caries experience higher in non-fluoridated areas compared to fluoridated areas.    Race and income-related gradients in caries experience observed in fluoridated and non-fluoridated areas.    Absolute inequalities were consistently lower in fluoridated areas compared to non-fluoridated areas.    Income related inequalities in caries was lower in fluoridated areas compared to non-fluoridated areas for Indigenous and non-Indigenous children.    Water fluoridation was associated with lower caries experience and reduced inequality amongst children. | Child caries experience were far greater among Indigenous and lower income households than their counterparts.  The levels of caries experience were far lower among children who were residents in fluoridated areas. | **S:**   Representative sample    Data collected using “internationally accepted approaches” for oral epidemiological examination and self-reported information.    Data weighted and standardised.    **W:**  Cross sectional study, therefore, causality cannot be established.    Missing data low – however those with missing data more likely to have caries experience and from low SES groups. |
| Do & Spencer, (2015)  Contemporary multilevel analysis of the effectiveness of water fluoridation in Australia | Australia  (English) | Cross sectional survey    Queensland Child Oral Health Survey 2010 –2012    Schoolchildren from 207 schools in 16 areas of Queensland, Australia      5–8-year-olds, n=2214  9–14-year-olds, n=3186 | Water fluoridation    Water fluoridation in Queensland   implemented between 2009-2011.      Townsville was the only fluoridated area.    All other areas defined as non-fluoridated areas in 2008-however,  scheduled for water fluoridation by 2011. | DMFS/ dmfs    Clinical data collected by 21 calibrated dental examiners at fixed/mobile dental clinics under standardised conditions | Queensland average dmfs was 4.23 and DMFS 1.47   Children of Indigenous origin, low-income households, with parental education at school level only, were found to have higher caries experience.    Lowest level of dental caries observed in the long-term fluoridated Townsville area.    In adjusted analysis Townsville children had significantly lower caries experience (RR for dmfs 0.61; 95% CI:0.44-0.82); RR for DMFS 0.60; 95%CI: 0.42-0.88), vs children in non-fluoridated areas | Lowest levels of dental caries were observed in long term fluoridation towns. | **S:**  Representative sample    Calibration of dental examiners    Multi-level analysis    Application of “strict criteria” during field work to improve consistently/ reliability of data and limit observer bias.    **W:**    Recall bias.    Self-reported data on SES factors and health behaviors    Cross-sectional study design – unable to establish a causal relationship. |
| Do et al., (2017)  Effectiveness of water fluoridation in the prevention of dental caries across  adult age groups | Australia  (English) | Secondary data analysis    Data extracted from the Australian National Survey of Adult Oral Health 2004-2006. Participants aged 15 to 91 years.    N= 4090 | Water fluoridation    Residential history used to calculate % lifetime access to water fluoridation (LAWF). Data collected by self-completion questionnaire | Clinical examination conducted by calibrated dentists.    Examination protocol based on NHANES protocol 2002.    DMFS calculated | Caries experience was strongly associated with age.    % LAWF was significantly associated with age in the lower age groups (15-34 and 35-44 year) only  Multivariable analysis showed that the highest % LAFW quartile had significantly lower DMFS count compared to the lower quartile:    15-34 age group: Those in the highest % LAWF quartile had 0.67 times lower mean DMFS compared to lower quartile.    35-44 age group: Those in the highest % LAWF quartile had 0.78 times lower mean DMFS compared to lower quartile. | Water Fluoridation was associated with lower caries experience in Australian adults who were young and middle-aged shows long term benefits. | **S:**   Sample considered representative.  **W:**   Recall bias.    No individual data on water consumption    Missing classification of DMFS may have misclassification error.    Low response rate (34%) |
| Eklund & Striffler, (1980)  Anticaries Effect of Various Concentrations of Fluoride in Drinking Water: Evaluation of Empirical Evidence | USA  (English) | Secondary data analysis    Individuals aged 12–14 years.    Data derived from Dean and associates’ data on 21 cities and data on other cities compiled by Striffler – In total data from 41 US cities were examined.    N = not mentioned | Water fluoridation | DMFT    Statistical modelling used to estimate the functional relationship between fluoride concentration greater than 0.1 ppm and reduction of DMFT. | DMFT reduction related to increased fluoride level.  Predictions modelled:    Increasing water fluoridation from less than 0.2 ppm to 1.0 ppm may result in a reduction of approx. 65% (5.6 teeth per capita) in DMFT. | Fluoride level was significantly related to DMFT within the range from approx. 0.1 ppm to 1.2 ppm. | **S:**  Compilation data from 41 US cities  **W:**  No single “best fit” line to predict the effects of fluoride adjustment to ideal levels from various endemic levels.    Unrecorded data may also impact the line of best fit such as diet and dental treatment. |
| Evans et al., (2009)  Water fluoridation in the Blue Mountains reduces risk of tooth decay. | Australia  (English) | Cross sectional survey at two time points    1993:  Children aged 5-11 years (baseline data), n = 1270    2003:  Children aged 5-13 years (follow up data), n = 1963 | Water fluoridation    Blue Mountain region (water fluoridation since 1992). Fluoride level adjusted to 1mg/L    vs    Hawkesbury region fluoridated since 1968 | Data collected via clinical examination by calibrated dentists.    Outcome variable: DMFT/ dmft    Caries defined as lesions that extend into dentine as per WHO/ICDAS 2 Code 5 criteria | Deciduous dentition:    Baseline 1993: Significant differences in dmft at a peak age of 8 years 2.92 vs 0.69 (Blue Mountain vs Hawkesbury).    In 2003 dmft 0.95 vs 0.74 (Blue Mountain vs Hawkesbury) but the result was statistically insignificant.     In 1993 the mean dmft for Blue Mountain children was 4.22 vs 2.99 for Hawkesbury children.    The percentage of children aged 5-8 years with caries experience reduced significantly from 56% to 27% in Blue Mountain (1993 vs 2003). Dmft reduced from 4.22 to 2.48 (1993 vs 2003)    The percentage of children aged 5-8 years with caries experience reduced from 35% to 29% in Hawkesbury (1993 vs 2003). Dmft reduction from 2.99 to 2.79 (1993 vs 2003)    Permanent dentition 8-11 years:    Baseline 1993: Significant difference in DMFT at 10 years 1.00 vs 0.56 (Blue Mountain vs Hawkesbury). For other age groups, no significant differences seen between areas.    The percentage of children aged 8-11 years with caries experience reduced from 35% to 12% in Blue Mountain (1993 vs 2003). DMFT reduced from 2.21 to 1.73 (1993 vs 2003) in Blue Mountain    The percentage of children aged 8-11 years with caries experience reduced from 24% to 16% in Hawkesbury (1993 vs 2003). DMFT reduction from 1.96 to 1.92 (1993 vs 2003) in Hawkesbury | Tooth decay reduction in fluoridation areas demonstrated the benefits of water fluoridation programme. | **W:**  Concerns expressed regarding the attenuation of results due to fluoride use and halo effect of fluoridated Hawkesbury region – however no attenuation found. |
| Fabruccini et al., (2016)  Comparative effectiveness of water and salt community-based fluoridation methods in preventing dental caries among schoolchildren | Brazil and Uruguay  (English) | Cross sectional study    Two population samples based on oral health surveys of 12-year-old schoolchildren:    2009-2010: Porto Alegre, Brazil: n = 1528    2011-2012: Montevideo, Uruguay: n = 1154    Schools a mixture of public/private | Porto Alegre, Brazil: Artificial water fluoridation    2007-2012: Mean F concentration 0.72 +/- 0.05mg F/L    Montevideo, Uruguay:    Fluoridated household salt  Mean concentration of fluoridated salt: 242 +/- 14mg F/L | Data collection at school by calibrated examiners    Outcomes: DMFT/ caries prevalence    Caries prevalence defined as % of children with at least 1 DMF tooth.    DMFT / caries prevalence based on two criteria: WHO and WHO modified criteria.    WHO criteria: only cavitated carious lesions recorded.    WHO modified criteria: Includes cavitated and active non-cavitated carious lesions. | Using modified WHO criteria: Crude estimates/ adjusted analysis showed a significantly mean higher DMFT amongst school children exposed to fluoridated salt vs water fluoridation:   Mean DMFT - Crude estimate: 2.79 vs 1.96; p=0.002 (salt fluoridation vs water fluoridation)    Mean DMFT - Adjusted estimate: 2.89 vs 1.82; p <0.001 (salt fluoridation vs water fluoridation)    After adjusted analysis, children exposed to salt fluoridation compared to water fluoridation had a significantly higher risk of caries (WHO criteria):    OR for caries prevalence: 1.61; 95%CI:1.26-2.07; RR for DMFT=1.32; 95% CI=1.16-1.51    Similar differences were also observed using modified WHO criteria. | In developing countries, fluoridated water provided a better protective effect against dental caries than household fluoridated salt among school children. | **S:**  This study used WHO modified criteria.    Representative samples with similar demographic characteristics    Both cities had no: caries prevention programme e.g., fluoride varnish application/ sealant restorations/ mouth rinsing or oral hygiene programme    Calibrated oral examiners.    **W:**  Lack of individual data – study used city-based fluoridation data. |
| Fyfe et al., (2015)  A cost effectiveness analysis of community water fluoridation in New Zealand | New Zealand    (English) | Cost effectiveness analysis    12 fluoridated communities in New Zealand (NZ) (population 420,616)    Communities with populations of:   <5,000 (small)  5,000–10,000(medium)  10,001–50,000 (large 1) and > 50,000   (large 2) | Community Water Fluoridation (CWF)    CWF  at a level of 0.7–1.0mg/l with treatment evaluated against a treatment only option for the prevention of dental caries. | Mean DMFT: fluoridated vs non-fluoridated communities.    Costs averted from CWF | The total annual equivalized  per capita cost of community water fluoridation for:  Small communities: $4.38  Medium communities: $1.23  Large communities: $0.66  Large 2 communities: $0.53    The difference in mean dmft/DMFT  between communities with and without  CWF was 1.0 dmft/DMFT (p<0.05) for children (<18 years) and 0.8 dmft/DMFT  (p<0.05), for total population    The annual equivalized per capita costs averted resulting from CWF was $4.82 for  total population and $5.21 for children    CWF was a cost-effective intervention for all populations but for smaller communities the effectiveness could be more marginal and depends on the profile of the community. | CWF was cost effective public health intervention in NZ but less effective in smaller communities. | **S:**  Sensitivity analysis was done.    **W:**  Limited response rate for questionnaires    Lack of information on lifetime exposure of fluoride    Confounding factors such as oral hygiene habits and other sources of fluoride were not controlled for. |
| Goodwin et al. (2024)  The CATFISH study: An evaluation of a water fluoridation program in Cumbria, UK | (England)  (English) | Longitudinal prospective cohort study  -*Birth cohort* of children conceived after water fluoridation introduction- observed for 5-6 years  (1444 participants)  -*Older cohort* of children age 5 years old after water fluoridation introduced  (1192 participants) | Water fluoridation:  -systemic and topical effects for birth cohort  - topical effects in older cohort | Proportion of children with evidence of caries in primary or permanent teeth (dmft/DMFT)  Mean dmft/DMFT count | *Birth cohort*:  **Intervention group:** 17.4% with caries experience  **Control group**: 21.4% with caries experience  After adjusting for deprivation, sex and age, lower odds of caries in intervention group (OR: 0.74, 95%CI 0.55-0.98)  *Older cohort:*  **Intervention group:** 19.1% with caries experience  **Control group**: 21.9% with caries experience  After adjusting for deprivation, sex and age, insufficient evidence for effect of intervention (OR: 0.80, 95%CI 0.58-1.09)  Beneficial effect of water fluoridation on dmft/DMFT count in both Birth (IRR 0.61 95%CI 0.44-0.86) and Older (IRR 0.69 95%CI 0.52-0.93) cohort:  No evidence to support differential effect of water fluoridation across deprivation quintiles | Impact of water fluoridation on caries prevalence is less than previously estimated | **S:**  Whole population longitudinal design, assessment of potential examiner bias, quantification of effect modifiers.    **W:**  Low response rates from questionnaire data    Lower prevalence of caries than predicted and interproximal lesions likely missed due to absence of radiographs.  Fluoridation dose delivered inconsistent/suboptimal at times. |
| Griffin, Jones & Tomar, (2001)  An Economic Evaluation of Community Water Fluoridation | USA    (English) | Economic evaluation of community water fluoridation    Oral health data from:  Studies from 1978-1988 and cited in Garcia’s 1989 review of the literature.  National Survey of Oral Health in US School children (1986/87); National Survey of Oral Health in Employed Adults and Seniors (1985/86); NHANES (1971-74; 1989/94)    Communities categorized as:  < 5000  5000 – 9999  10 000-20000  >20000.  Age=0-65+  Cost of water fluoridation: Obtained from a published study (1992) reporting one-time fixed / annual operational costs for 44 Florida communities that implemented water fluoridation in the 1980’s. | Water fluoridation | Local cost saving as a result of community water fluoridation.    Economic evaluation examined the per person net cost resulting from one year of exposure to water fluoridation.      If net cost is negative, then water fluoridation is cost saving | With “base case assumptions” the annual per person cost savings ranged from $15.95 in very small communities to $18.62 in large communities.    Fluoridation was “cost saving for communities of any size” considering “increment, effectiveness, or the discount rate to take on their worst-case values individually”.    For small communities (population less than 5000), fluoridation was still considered cost saving, although cost saving smaller in comparison to larger communities: $0.42 to save 0.04 tooth surfaces or $10.50 per tooth surface per year.    Overall water fluoridation “offers significant cost savings”. | Water fluoridation (WF) offered significant cost savings. The reduction in costs of restorative care due to averted disease exceeded the cost of WF in communities in any size. | **S:**   Analysis based on the most current data available.    Findings considered robust.    **W:**  “Obtaining good estimates of caries increments in non-fluoridated communities was difficult” - leading to estimation bias.    NHANES data used- possible under estimation of caries increment in communities with fluoridation.    Cost estimates difficult to identify and measure empirically, leading to bias in cost figures. |
| James et al., (2021)  Impact of Reducing Water Fluoride  on Dental Caries and Fluorosis | Ireland  (English) | Before and after study/ Comparative study.    Comparison of caries and fluorosis at 2 time points:    2002  Random sample of children aged 8 years old selected from the North South Survey of Children’s Oral Health  Dublin (n=679)  Cork-Kerry (n=565)    2014-2017  Random sample of children aged 5 years old – followed up to aged 8.  Selected from the Fluoride and Caring for Children Teeth (FACCT study 2014)  Dublin (n=707)  Cork-Kerry (n=1148) | Community water fluoridation (CWF)- defined as:    Full lifetime exposure to CWF  -No exposure to CWF    In 2002 CWF concentration 0.8 to 1.0 ppm F + no tooth brushing guidance    In 2014 CWF concentration 0.6 -0.8 ppm F + toothbrushing guidance    Toothbrushing guidance introduced in (2002) to reduce ingestion of fluoride toothpaste: delay toothbrushing till after 24 months, pea sized amount of toothpaste and supervised brushing. | Caries: Dental caries defined using WHO (2013) criteria and modified to include visible non cavitated dentinal caries.    Caries experience defined as proportion of children with decayed, missing or filled primary canines; first or second primary molars (dmft). Mean dmft also considered.    Fluorosis:  Dean’s index score of “very mild” or higher – in the permanent dentition. | No change in commencement of fluoride toothpaste <24 months following introduction of toothbrushing guidance.    Fluorosis predominantly “mild” with no significant differences between 2002 and 2017    Amongst children with CWF no significant difference in caries prevalence/ severity between 2002 and 2017    Caries severity was less in 2017 compared to 2002 (mean dmft 4.2 vs 4.9) amongst children with no CWF (p=0.039).    The difference in caries severity between children with full CWF and no CWF was less in 2017 vs 2002 (p=0.013) - suggesting reduced benefit of CWF in 2017. | With the introduction of policy measures there was no increase in caries but the expected reduction in dental fluorosis was not observed. | **S:**   Individual classification of exposure to CWF    Assessment of caries/ fluorosis using identical protocol at both time points and with training led by the same “experienced benchmark examiner”.    Control groups at both time points    Multivariate regression analysis to control for sociodemographic covariates.  **W:**   Examiners were not blinded to participants water fluoridation status.    Recall bias.    Shortcoming in Dean’s Index – whole mouth score not recorded. |
| Jones, (2000)  The effect of water fluoridation and social deprivation on tooth decay | England  (English) | Ecological study  National Health Service 5-year-old-children dental epidemiological surveys in 1991/2 and 1993/4, coordinated by the British Association for the Study of Community Dentistry,  N = 10,004 | Comparing fluoridated and non-fluoridated areas and the effects on dental health (tooth decay)    Newcastle (naturally fluoridated), Hartlepool Vs Non fluoridated areas (Salford + Trafford)      Controlled for social deprivation using Jarman score | DMFT of the 5-year-olds.  The Jarman  underprivileged area score was used as a proxy for social deprivation and any relationship to dental decay  was explored. | Mean decayed, missing and filled teeth scores ranged from:    0.24 to 4.32 in Salford & Trafford;  0.35 to 2.54 in Newcastle & North Tyneside;  0.23 to 0.9 in Hartlepool.    The UPA scores ranged from:    -22.9 to 48.65 in Salford & Trafford;  -17.18 to 56.59 in the 34 wards in Newcastle;  -30.7 to 40.65 in the 17 wards in Hartlepool.    Tooth decay was associated with social deprivation and water fluoridation is more effective in deprived areas.    A predicted national reduction in decay of 44% was found meaning 5-year-old children would have 44% less tooth decay in England if water fluoridation was introduced in the1970s. In the very deprived areas this prediction would increase to 54% | Water fluoridation programme, especially in areas of social deprivation areas, had been effective in halving tooth decay in 5-year-old children. | **S:**  Had 3 comparable groups of water fluoridation within the same country.    **W:**  Did not consider the fluoride history of the children who may have moved between areas of fluoridation/non-fluoridation.    No measure of applied fluoride/ dietary intake by children. |
| Kim et al., (2017)  Associations of Community Water Fluoridation with  Caries Prevalence and Oral Health Inequality  in Children | South Korea    (English) | Cross sectional study  Primary schools’ children aged 6, 8, and 11 years old in the CWF and non-CWF neighbouring areas, n = 1,411 | Community water fluoridation (CWF) | DMFT, DMFS, pit-and-fissure DMFS, and smooth surface DMFS | 8- and 11-year-old children in CWF area showed significantly lower mean DMFT scores compared to those in non-CWF area.    8- and 11-year-olds living in the CWF area had significantly lower DMFT scores (0.15) compared to those living in the non-CWF area (0.56) (p <0.001).    11-year-olds in the CWF area also showed  lower mean DMFT scores (0.86) compared to those in non-CWF area (1.43) (p<0.001).   8-year-old children in the CWF area (0.13) demonstrated significantly lower pit-and-fissure DMFS scores than those in the non-CWF area (0.52) (p < 0.001). A similar finding was also observed amongst 11-year-old children in both areas (CWF and non-CWF areas: 0.75 and 1.27, respectively; p < 0.001).    8-year-old children in the CWF area (0.09) had significantly lower smooth surface DMFS scores than those in the non-CWF area (0.0.28) (p = 0.004). | CWF programmes were effective in preventing caries on permanent teeth and can reduce oral health inequalities in children. | **S:**   FAS (Family affluence scale) was developed to assess SES.    Major confounding variables were adjusted using ANCOVA.    **W:**  Despite using ANCOVA, other confounders could affect the results such as the duration of exposure to fluoride    The reliability of the associations between  FAS and SES in the analysis of oral health survey was not established. |
| Koh et al., (2015)    Effects of Water Fluoridation on Caries Experience in the Primary Dentition in a High Caries Risk Community in Queensland, Australia | Australia    (English) | Interventional study    Pre-fluoridation:  Randomly selected children aged 4-9.9  years attending primary school and routine dental treatment from 1998 – 2008, n = 201    Post fluoridation:  Randomly selected children aged 4-9.9 years attending primary school + routine dental treatment from 2011-2012, n = 256 | Water fluoridation (WF)    Data collection 36.55 ± 6.53 months post exposure to water fluoridation. | Data collected from clinical examination records and bitewing radiographs by dental clinical staff. Intra-examiner and inter-examiner kappa value: 0.99 and 0.89, respectively.    Outcome variable: caries experience - dmfs/ dmft   Decay on bite wing radiographs assessed using the maxillary and mandibular primary molar teeth. Decay defined as caries extending into dentine. | Pre fluoride caries experience 87% (dmft>0) vs post fluoride caries experience 75% (p=<0.001)    Children exposed to water fluoridation were significantly more likely to have a lower dmft (OR=0.44; 95CI: 0.27-0.72)    Mean total post F dmft scores for children aged 4.00-6.99 and 7.00-9.99 represented reductions of 22% and 17% compared to respective pre-F mean scores.    Overall, the mean dmfs (proximal surfaces of primary molars) showed a statistically significant reduction of caries experience 2.88 (fluoridated) vs 3.4 (non-fluoridated); p=0.046    Overall, the mean dmfs (occlusal surfaces of primary molars) showed a statistically significant reduction of caries experience 1.1 (fluoridated) vs 1.43 (non-fluoridated); p=0.018). However, this was mainly due to caries reduction in the second mandibular molars | After 36 months of WF there was a significant drop in caries prevalence and a reduction in caries experience in a community with one of the highest caries rates in Australia. | **S:**  The study employed a “historical design” so that pre and post data could be analyzed.    Clinical radiographic data used to assess disease prevalence using a “well tested, sensitive and reproducible scoring system”.    No change in quality of dental records, caries diagnosis and caries treatment between pre and post fluoridation period    Residential mobility noted however most participants remained within the district, with only 12% moving to a different state and 3% moving to a different part of Australia.    Sugar consumption/ fluoride use is likely to have remained similar during the two time periods.  **W:**  The factors that can influence caries rates such as socioeconomic status, diagnostic and treatment criteria and diets might have changed between the time periods. |
| Lalloo et al., (2015)  Does fluoride in the water close the dental caries gap between Indigenous and non-Indigenous children? | Australia    (English) | Cross sectional study.    Data for Queensland (Qld), South Australia (SA), Western Australia (WA), Tasmania (Tas), the Northern Territory (NT) and the Australian Capital Territory (ACT) were sourced from the Child Dental Health Survey (CDHS) conducted in the 2010.  Children aged 5 to 15 years.    ‘Aboriginal’, ‘Torres Strait Islander’, ‘Aboriginal and Torres Strait Islander’ or ‘South Sea Islander’ responses are grouped together and referred to as Indigenous.  All other children are referred to as non-Indigenous.    N = 97 809 | Water fluoridation (WF)    Water fluoride concentration categorized as two groups:    Fluoride level <0.3 mg/L and fluoride level equal to or greater than 0.5mg/L | Three oral health outcomes: caries in deciduous dentition (5-10 years); caries in permanent dentition (6-15 years); and presence of fissure sealants    Dmft/ DMFT    Oral health data collected by dental therapist / dentist based on “clinical judgment” | For all measures of dental caries Indigenous children (vs non-indigenous children) were significantly worse off: mean dmft 3.84 vs 2.22 / mean DMFT 2.00 vs 1.08.    Effect of fluoridation was greater amongst non-Indigenous children:    Mean dmft score in fluoridated water 1.70 vs non fluoridated water 2.86 - % difference of 50.9%    Amongst Indigenous children:  Mean dmft score in fluoridated water 3.29 vs non fluoridated water 4.16 - % difference of 23.4%    Amongst non-Indigenous children:   Mean DMFT score in fluoridated water 0.68 vs non fluoridated water 1.58 - % difference of 79.7%    Amongst Indigenous children:  Mean DMFT score in fluoridated water 1.59 vs non fluoridated water 2.23 - % difference of 33.5%    Water fluoridation was found to be effective in reducing dental caries but did not reduce the inequality gap between Indigenous and non-Indigenous. | WF was an effective programme at reducing dental caries but did not close the gap between Indigenous and non-Indigenous children’ deciduous dentition caries. | **S:**  The study addressed important topics of often neglected indigenous children’ oral health  **W:**   Approximately 5% of records were missing from analysis on fluoride exposure/ Indigenous status    Data on lifetime exposure to fluoride not available    Two large Australian states (New South Wales and Victoria) not included in analysis – therefore findings might not be generalizable to the population. |
| Lee, Faundez & Losasso, (2020)  A Cross-Sectional Analysis of Community Water Fluoridation and  Prevalence of Pediatric Dental Surgery Among Medicaid Enrollees | USA    (English) | Cross-sectional study    Data sourced from Medicaid claims data for children aged 9 years and younger enrolled in either a fee-for-service or managed care plan through their state’s Medicaid program (2011-2012)  n = 436 counties within 5 states per year (872 county-year observations), were  included in the analysis | Water fluoridation    Defined as a fluoride level of 0.6mg/L (parts per million) or greater.    Continuous variable formed estimating the proportion of a county's population with access to water fluoridation (0%-100%) | Primary outcome:  Receipt of caries-related surgical treatment with general anesthesia (DGA)    Secondary outcome:  Prevalence of caries related visits | Adjusted analysis showed that a 10% increase in the proportion of a county's access to CWF was associated with lower caries-related visit prevalence (p<0.001)    Increasing CWF access in 10% increments was associated with decreased DGA prevalence in unadjusted analysis (p=0.006), but not in adjusted analysis (p=0.07). | Increased access to CWF was associated with decreased caries related visits and dental surgery. | **S:**   Adjusted analysis.    **W:**  Results may not be generalizable to the population.    Data limited to 5 states.    Geographical bias-data related to coastal areas of US.    County data therefore unable to ascertain individual access to fluoridated water.    Observational study therefore causality cannot be inferred.    Limited budget, therefore additional years were not analyzed.    Study design did not address an exposure-dose response. |
| Lee & Han, (2015)  Exploring the determinants of  secular decreases in dental caries  among Korean children | South Korea    (English) | Cohort study    Data from 3 waves of Korean National Oral Health Survey (KNOHS) between 2003 and 2010.    N=23059 children aged 8,10,12 years | Community Water fluoridation    Sealants | Caries experience  Mean DT  Mean FT  Mean DMFT | The prevalence of caries experience decreased from:    43.2% to 27.2% in 8-year-olds  61.5% to 46.2% in 10-year-olds  75.9% to 60.5% in 12-year-olds    These trends were also same with mean number of decayed teeth (DT) among all age group, filled teeth (FT) in 8-year-olds, and decayed, missing, filled teeth (DMFT) amongst all age group. | Population declined in dental caries in Korean children aged 8-12 years from 2003-2010 among all age and gender groups. | **S:**   Nationally representative data    **W:**  Due to different  variables in each survey from 2003 to 2010, the number of overlapping common variables was limited.    Potential confounding variables that could possibly explain the downward trends of  dental caries was not surveyed.    Sampling error - the sampling design of  KNOHS was to take one school from each stratum, in contrast to the usual sampling design which takes at least two clusters from each stratum so that strata variance calculations can be performed and combined into overall variable variance calculations for population estimates. |
| Lee & Dennison, (2004)  Water fluoridation and dental caries in 5- and 12-year-old children from Canterbury and Wellington | New Zealand    (English) | Cross sectional study  Data of children aged 5- and 12-years oral health status from the School Dental Services in two study areas: non-fluoridated areas (Canterbury) vs fluoridated areas (Wellington)    N=8030  Age=5-year-old    N= 6916  Age=12-year-old | Water Fluoridation (WF) | Caries prevalence and severity | 5 years old: in fluoridated areas had 2.63 dmfs (sd, 5.88) compared to those in the non-fluoridated area 3.80 dmfs (sd, 6.79).  12-year-olds figures were 1.39 DMFS (sd, 2.30) and 2.37 DMFS (sd, 3.46) (fluoridated vs non-fluoridated areas)    Non-fluoridated group, the mean dmfs  score of Maori five-year-olds was double that for the “Other (European)  group, and that for Pacific children, three times greater than “Others” group    Markedly lower dental caries severity scores at both ages for Māori and Pacific children living in fluoridated areas    5-year-olds  Water fluoridation associated with 31% lower dental caries severity scores in dmfs amongst 5-year-olds.    Fluoridated water supplies were consistently associated with lower dmfs scores for males and females separately; amongst all ethnic groups; and socio-economic groups    12-year-olds  Water fluoridation was associated with 41 percent lower dental caries severity scores in permanent teeth (DMFS) for the group as a whole. | Children living in fluoridated areas had significantly better oral health compared to those not in a fluoridated area. | **S:**   Data was based on dental records rather than health surveys.    Large and representative population    Unintentionally examined ethnicity and oral health inequalities    **W:**  Only children who had dental records included in study.    Children misclassified in SES – those from low SES groups attended more advantaged schools. |
| Levy et al (2023)  Association of Nationwide Water Fluoridation, changes in dental care  legislation, and caries-related treatment needs: A 9-year record-based  cross-sectional study | Israel (English) | Cross-sectional study.  Dental records of 34,450 soldiers recruited into military service between  2012 and 2021. | National water fluoridation and the initiation of free dental coverage for children.  The study divided the population into three groups according to  year of birth as below:   - Group A recruits were born after 2001 and enjoyed both fluoridation and free dental care. - Group B recruits were born between 1996 and 2000 and enjoyed water fluoridation at a young age but not free dental care. - Group C recruits were born in and before 1994 and enjoyed limited benefits of national fluoridation (started in 2002) and none of free dental care reforms. | 1. number  of teeth requiring restorations  2. number of teeth requiring root  canal treatment (RCT)  3. number of extractions required due to caries. | A multivariate generalized linear model (GLM) revealed that male sex, older age, low  intellectual capability score (ICS), and low socioeconomic cluster (SEC) were significant predictors for greater caries-related treatment needs (P < 0.001).  The study findings indicated that subjects exposed to fluoridated water during their childhood had significantly lower rates of caries-related treatment, regardless of access to free dental care. | Mandatory water fluoridation was associated with significantly lower caries-related treatment needs  while national dental health legislation providing free dental care to children and adolescents was not. | **S:**  the dental services provided by the IDF military are not financially based on a fee-for-service model. Instead, all dental plans are solely based on medical necessity, promoting a consistent clinical approach for the oral health data.  W:  The  study population was comprised essentially of healthy young adults which may not reflect the general population. |
| Mahoney et al., (2008)  Lifetime fluoridation exposure and dental caries experience in a military population | Australia    (English) | Cross sectional study    Annual dental examination and residential locations 1964-2003    N = 876 deployable Australian Defense Force (ADF) personnel aged 17 – 56 years. | Residential water fluoridation exposure | DMFT | Mean DMFT (±95% confidence interval) was:  6.3 ± 0.8 for <10% fluoridation exposure, 7.8 ± 0.8 for 10% to <50% exposure,  7.5 ± 0.7 for 50% to <90% exposure  4.6 ± 0.6 for 90% exposure (P < 0.01)   However, age was inversely associated with mean DMFT and in the <10% exposure group, 91% of people were aged <35 years.    Service rank was also significantly associated with both fluoridation exposure and DMFT. | Degree of lifetime exposure to fluoridated drinking water was inversely associated with DMFT in a dose–response manner among adult military population. | **S:**   Large age group over a long time period.    Included use of fluoride supplements    **W:**  Required address for deployed personnel – moving location.    Small female population    Population not representative of general population – healthier    Participants received free dental care. |
| Moore et al., (2017)  The costs and benefits of water fluoridation in NZ | New Zealand    (English) | Cost benefit and cost effectiveness analysis    Data extracted from Australian National Survey of Adult Oral Health    Time horizon 20 years | Community Water Fluoridation | Quality adjusted life years    Cost effectiveness | Cost effectiveness:    For neighborhood and small plants, the cost of fluoridation was greater than the estimated cost offsets from  averted dental costs.    For minor through to large plants, there was a net cost saving. For a large plant  supplying 50,000 people, the cost offsets are over 20 times the cost of fluoridation.   While fluoridating reticulated water supplies for large communities was cost-effective, it is unlikely to be with populations smaller than 500 people.    Following averted treatment if CWF  were to be implemented at all plants supplying populations over 500:    459,000 teeth with untreated caries;  4,068,000 extractions;  3,361,000 restorations.    Estimated national net saving from universal fluoridation of supplies for populations over 500 over 20 years to be $1401 million.    The saving to the health budget would be $149 million. Most of the savings would come from a $1428 million reduction in private dental care expenditure.    QALY estimations that the provision of fluoridation over 20 years to all reticulated water supplies supplying populations of 500 or more would result in 8800 to 13,700 quality-adjusted life years. | Community water fluoridation remained highly cost-effective for all but very small communities. The health benefits while (on average) small per person added up to a substantial reduction in the national disease burden across all ethnic and socioeconomic groups. | **S:**  Looked at large range of community sizes.    Adjusted analysis for population growth in the next 20 years.    **W:**  Usage of Australian National Survey of Adult Oral Health and applied to NZ adults are most previous literature based on children.    Not an evaluation but a cost effectiveness analysis. |
| Peres, Antunes & Peres, (2006)  Is water fluoridation effective in reducing inequalities in dental caries distribution in developing countries? Recent findings from Brazil | Brazil  (English) | Analysis of secondary data of epidemiological survey.  Countrywide Brazilian epidemiological survey of oral health 2002-2003 of twelve-year-old school children, N = 34,550 | Community water fluoridation | SES differences.   Assess SES differences between areas with and without water fluoridation.    Dental caries among SES strata | Better-off towns tended to present a higher coverage by the water supply network and were more inclined to add fluoride.    Fluoridated tap water was associated with an overall improved profile of caries, concurrent with an expressively larger inequality in the distribution of dental disease.    The proportions of caries-free individuals (38.96 %), the DMFT index (2.21) and the proportion of high-caries children (29.29 %) presented prominently improved figures in towns with the most elevated coverage of  pipe water, when compared to those estimated for the towns with the most limited water supply network (respectively  20.09 %, 3.53 and 40.27 %) | Towns which added fluoride to their water supplies presented an expressively better profile of dental caries indices than  those that did not | **S:**  Urban and rural areas included.    Public and private schools included.    **W:**  Sample population 12-year-old school children – not representative sample of school age children. |
| Schluter et al., (2020)  Association Between Community Water Fluoridation and  Severe Dental Caries Experience in 4-Year-Old New Zealand Children | New Zealand    (English) | Cross-sectional geospatial study  The participants were New Zealand children aged 4 years who had Before School Check (B$SC) assessment between fiscal years (2010 to 2011 through 2015 to 2016, inclusive, N = 275,843 | Community water fluoridation (CWF) | Severe caries experience | Severe caries was identified for 24 226 children (15.8%) in fluoridated and 17 135 children (14.0%) in unfluoridated areas, yielding an unadjusted odds ratio of 0.93 (95% CI, 0.90-0.95).    adjusted analyses, children residing in areas without fluoridation had higher odds of severe caries compared with those within fluoridated areas (odds ratio, 1.21; 95% CI, 1.17-1.24) when ethnicity, age, residential location, sex added to the model | Community water fluoridation was associated with reduced prevalence of severe caries in the primary dentition of New Zealand’s 4-year-old children. | **S:**  Near whole population level study    CWF exposure evenly spilt.    **W:**  Children’s residential address based on B4SC assessment, but 45% children move more than once    Caries experience was based on ‘lift the lip’ which is not clinically validated tool.    Diet and oral healthcare data were unavailable for participants. |
| Slade et al., (1996)  Influence of exposure to fluoridated water on socioeconomic inequalities  in children's caries experience | Australia    (English) | Cross sectional study    Participants were randomly selected from the State school dental service and received dental examinations between June 1991-May 1992  Queensland children  aged 5-12 years (Brisbane/ Townsville), N = 6704      South  Australian children  aged 5-15 years (Adelaide/ Non-Adelaide South Australia), N= 6814 | Lifetime exposure to water fluoridation    Children categorized into 3 groups:    100% exposure for children who consumed 1 ppm F all their life.    0% exposure for children who consumed 0 ppm F all their life.    1-99 % exposure for children who consumed 1 ppm F, for only part of their life or lived in cities/ towns with suboptimal fluoride concentration (for example 0.5 ppm F) | Clinical data collected by non-calibrated dentists/ dental therapists at periodic dental examinations.    Outcome variable: caries experience (dmfs/ DMFS) using WHO criteria (WHO, Oral health surveys. Basic methods (1987) | In both states dmfs/ DMFT was significantly higher in children from low SES groups compared to high SES groups (p<0.01) (SES categorized by parental education/ household income)    Independent effects of income/ education remained significant (p<0.01) after controlling for exposure to fluoride in drinking water.    In Queensland, there was significant multiplicative interaction whereby SES inequalities were lower amongst children exposed to fluoride: dmfs ratios between low and high-income groups ranged from 1.54 to 1.36 for children with no exposure to fluoride and from 0.84-2.07 for children with lifetime exposure to fluoride.    In South Australia absolute differences in caries experience between low and high SES children were greater amongst non-exposed fluoride groups due to the higher level of caries experience of children with no exposure to water fluoridation | Children from lower SES groups had experience greater levels of dental caries. SES related inequalities were greatest, number of tooth surfaces affected, among children with no exposure to fluoride in drinking water. | **S:**  Higher level of non-response associated with household income impacting “completeness” of SES data.    **W:**  No information on dietary intake |
| Spencer et al., (2017)  Preventive benefit of access to fluoridated water for young  adults | Australia    (English) | Cross-sectional study    Data extracted from the Australian National Child Oral Health Survey (NCOHS) - population based study of child oral health of children aged 5-14 years (2012-2014)    N = 24 664 | Water fluoridation    Fluoride concentration of 0.7 ppm or more considered in analysis.    Lifetime exposure to fluoridated water (LEFW) categorized into 4 groups:  0%; >0%-50%; >50%-<100% and 100% | Oral health examinations conducted by calibrated dental examiners at fixed and mobile examination dental clinics under standardised conditions.    Primary outcomes: dmfs/ DMFT  Caries prevalence    Caries defined as: untreated decay captured at cavitation and / or dentinal involvement. Visual criteria applied. | Fluoride exposure groups were significantly associated with SES.    Higher proportion of low-income groups/ low parental education/ Indigenous status had 0% LEFW compared to 100% LEFW.    Adjusted estimates showed “significant declining trends of both the prevalence and experience of caries in either dentition (primary/permanent) with increasing per cent LEFW”   Adjusted estimate 5-8 years: mean dmfs 4.10; 95% CI 3.71-4.53 (0% LEFW) vs 1.95;95%CI 1.73-2.20 (100% LEFW)   Adjusted estimate 9-14 years: mean DMFT 1.28;95%CI 1.15-1.42 (0% LEFW) vs 0.7; 95%CI 0.63-0.78 (100% LEFW) | Consistent association between lifetime exposure to fluoridated water and measures of caries prevalence  and experience in both the primary dentition of 5-8-year-old and permanent dentition of 9-14-year-old Australian children. | **S:**  Large sample size    Weighted national data set.    Results generalizable to the population.    Calibrated examiners    **W:**    Recall bias.    Risk of misclassification due to lack of sensitivity testing |
| Wright et al., (2001)  The cost-effectiveness of fluoridating  water supplies in New Zealand | New Zealand and USA    (English) | Economic methodology: Cost effectiveness analysis of water fluoridation    New Zealand children aged 4-12 years:  Children receiving fluoridated water, n= 29,097.  Children receiving non fluoridated water, n = 46,825  For adult data (aged 14-34) US data used as no New Zealand data on adult dental benefits associated with fluoridation. The study population was Washington State employees and their spouses, aged 20 to 34, n = 10,628 | Water fluoridation from 2000-2030 | Estimate the minimum population for which the oral health benefits from water fluoridation would be greater than water treatment costs | For both the deciduous and permanent dentition, children living in fluoridated areas vs non-fluoridated areas had fewer fillings at every age from 4 to 13 years.  However, Māori children compared to non-Māori children were approximately two times more likely to have filled teeth.    Fluoridation remained cost saving (dental cost savings exceed water fluoridation costs) for communities above 1000 people. However, a “true break-even community size may be lower”.    For smaller communities, fluoridation may be considered cost effective depending on the “non-monetized value assigned to an averted decay surface”.    Effectiveness of water fluoridation is higher for low socioeconomic groups.    Fluoridation remained “very cost effective” particularly for communities with a: “high proportion of children, Māori or people of low socio-economic status”. | Fluoridation remained very cost effective for communities of 1000 people and above, particularly so for communities with  high proportions of children, Māori, or  people of low socio-economic status | **S:**  Data used reflected decay rate reductions in both fluoridated and non-fluoridated areas – authors were not aware of any other previous cost effectiveness analysis that had done this.    Dental treatment savings were included as a negative cost. “Other costs effective analysis had been based solely on the cost of fluoridating water supplies”.    Substantial time horizon – 30 years.    Estimated benefits up to adults of 45 years.    Other cost-effective analysis: “shorter time horizons” and “based on benefits to children only”.    **W:**  Underestimation of results, as the benefit of fluoridation extends over the life course.    US data used – employees/ spouses more likely to have dental insurance/ better tooth conditions compared to NZ data.    Implicit assumption that the cost of water fluoridation and cost of dental restorative treatment experience the same rates of inflation from 2000-2030 – no future data on this.    Uncertainties in the cost of fluoridation.    Assumption made that all averted dental treatment are simple restorations – which would underestimate averted costs in teenage/ adult years due to the possibility of more complex restorations |

**Tobacco control polices N = 3**

| **Authors (year)**  **Title** | **Country of origin (published language)** | **Methods:**  **Type of study, study population, and sample size** | **Nature of upstream intervention** | **Outcome measures** | **Main findings** | **Conclusion** | **Strengths (S) and Weaknesses (W)** |
| --- | --- | --- | --- | --- | --- | --- | --- |
| Bassi, Yadav & Arora, (2013)  Strengthening Implementation of Tobacco-free Policies to Restrict Youth Access to Tobacco Products: Moving Towards Tobacco-free Generation in Bihar, India  Note: *Grey Literature* | India    (English)    *Note: Conference abstract* | Before and after study        Study population was minors of the state of Bihar.  Unknown sample size | Government directives to make Tobacco free educational institutes in the state of Bihar    Sustained advocacy | Knowledge of the provision of the law banning sale of tobacco to and by minors    Sale of tobacco products to minors | The knowledge of the provision of the law banning sale of tobacco products to and by minors increased by 23% (p<0.001).    Sales of tobacco products to and by minors reduced from 54.6% to 3.6% and 59.3% to 4%, respectively.    Sales of tobacco products within 100 yards of educational institutions reduced from 42.9% to 3.4%;    Absence of warning boards at points of sale and outside educational institutions reduced from 51.7% to 24.9% and 77.4% to 7.8%, respectively. | Supportive state government committed district administration and effective civil society engagement were pivotal in creating institutional mechanism for enforcement of the Indian tobacco control law in the state. | **S:**  Evaluation of the directives pre/ post intervention    **W:**  No details of the numbers interviewed pre/post intervention.    No details of the details of the legislation; when it was implemented; and when the study was conducted. |
| Gredner et al., (2020)  Impact of Tobacco Control Policies on Smoking-Related  Cancer Incidence in Germany 2020 to 2050—A  Simulation Study | Germany    (English) | Simulation study    German population aged 15-75+ years  Men/ women    2020, n = 81.4 million  2050, n = 71.9 million | Evidenced based tobacco control policies considered using the WHO Framework Convention on Tobacco Control    Different policy intervention scenarios considered:   - Cigarette price increase (single and repeated price increase) - Comprehensive marketing ban - Plain packaging | Cancer burden    “To estimate the number and proportions of potentially avoidable cancer cases under different policy intervention scenarios” | Baseline prevalence of smoking in men (22.3%) and women (15.3%) in 2017    After combining all tobacco control policies smoking prevalence was projected to decline to 9.7% in men and 6.7% in women by 2050    17.7 % of oral cancer cases were estimated to be preventable.    Estimated lip, pharynx, and oral cavity cancer cases (2020-2050): 307 978 (men) vs 133 706 (women)    Over a 30-year period (2020-2050) the following number of preventable smoking-related cancer cases (lip, pharynx, oral cavity) estimated:    Plain packaging: 17 922 cases  Comprehensive marketing ban: 9622 cases  Single price increase + 10%: 4840 cases  Repeated price increase+10% x 10: 34 304 cases  Combined tobacco control measures: 56 837 cases    Women:  Plain packaging: 6690 cases  Comprehensive marketing ban: 3595 cases  Single price increase + 10%: 1809 cases  Repeated price increase+10% x 10: 12 767 cases  Combined tobacco control measures: 21 189 cases    The most effective single intervention was estimated to be annual 10% price increase in cigarettes over 10 years, which may prevent about 8.5% (men) and 7.3% (women) smoking related cancer cases. | Over a 30 year 13.3%of smoking related cancer cases could be prevented in tobacco control policies were to be implemented. | **S:**  First German modelling study of this kind using nationally representative data.    Straightforward modelling framework to allow for future data comparisons.    **W:**  Not all tobacco control policies considered such as smoke free legislation.    For repeated price increase, price elasticity assumed to be constant over time.    Possible under/ over estimation of results when analyzing a combination of tobacco control policies, due to possible synergistic/ attenuating effects.    The study considered lag and latency time periods to consider the delay in effect of tobacco control policies on cancer incidence.  20-year time shift set; however, this figure is not considered definitive.    Occasional smokers/ passive smoking not considered in the study.    Youth not considered in the study. |
| Hagen et al. (2025)  MPOWER Tobacco control policies’ effects on lip and oral cavity cancer trends in MERCOSUR countries | MERCOSUR countries (South American trade bloc)  Argentina  Brazil  Paraguay  Uruguay  Venezuela  English | Ecological study, time series analysis from 2005-2021 (5 country cross comparison)  No information about the sample size | WHO MPOWER tobacco control policies measure scores  (Increased score=increased quality or intensity of measure) | Trends in Lip and Oral Cavity (LOC) cancer incidence, mortality, and DALYS- all measured through annual percent change | Median MPOWER scores in Paraguay (15) and Venezuela (21.5) lower in comparison to Argentina (26.0), Brazil (27.5) and Uruguay (27.0) .  LOC trends in Argentina, Brazil and Uruguay decreased or stationary for incidence, mortality and DALYS among men.  LOC trend for incidence increased for women in Uruguay only  LOC incidence trends increased for men and women in Paraguay and Venezuela; stationary mortality and DALYS trends for men and women (no decreasing trends noted in Paraguay or Venezuela) | Countries with ‘more rigorous’ MPOWER policies also have decreasing LOC burden | **S:** Cross country comparison over time  **W:** Descriptive analysis of secondary data using estimates (GBD study)  Does not account for other factors which contribute to LOC (SEP, diet and alcohol consumption)  Insufficient length of time for analysis to observe impact of tobacco control policies on LOC rates |

**Tobacco and Alcohol control policies N =1**

| **Authors (year)**  **Title** | **Country of origin (published language)** | **Methods:**  **Type of study, study population, and sample size** | **Nature of upstream intervention** | **Outcome measures** | **Main findings** | **Conclusion** | **Strengths (S) and Weaknesses (W)** |
| --- | --- | --- | --- | --- | --- | --- | --- |
| Herrera-Serna et al., (2019)  Efecto de Las Politicas de Control de Factores de Riesgo Sobre La Mortalidad por Cancer Oral En America latina  (Effect of risk factor control policies on oral cancer mortality in Latin America) | Latin America and Caribbean:  Argentina  Bolivia  Brazil  Chile  Colombia  Costa Rica  Cuba  Dominican Republic  Ecuador  El Salvador  Guatemala  Haiti  Honduras  Mexico  Nicaragua  Panama  Paraguay  Peru  Uruguay  Venezuela    (Spanish) | Ecological study  (Country-level – 20 Latin American countries)  No information about the sample size | Country status on the WHO Framework Convention on Tobacco Control    -Tobacco and alcohol control policies | Tobacco implementation of at least three WHO MPOWER measures:   - Monitoring tobacco use - Protecting People from tobacco smoke - Quitting tobacco - Warning about the dangers of tobacco - Enforcing tobacco advertising, promotion, sponsorship bans - Raising taxes on tobacco     Alcohol:  Implementation of policies:   - Restricting time/days of sales; - Indirect taxes; - Regulation of advertising | Tobacco: Stronger correlations were found between oral cancer mortality and prevalence of smoking when implementation of MPOWER strategies did not occur (r=0.738) compared with scenarios in which strategies were implemented (r=0.465); (p<0.050).    Alcohol: Stronger correlations between oral cancer mortality and prevalence of alcohol intake were found when regulations on time/days of sale and on advertising were absent (p=>0,73; p<0,05) compared to when regulations were implemented (p=>0,55; p<0,005).    No statistically significant correlation between oral cancer mortality and prevalence of alcohol intake was found when implementation on indirect taxes was absent (p=>0,579; p<0,076). However, a statistically significant correlation was found when implementation had taken place (p=>0,652; p<0,000). | The effect of the implementation of control policies was evidenced by a greater relationship with oral cancer mortality in the countries with the least progress in their execution. | **S:**  20 countries included.  Trend analysis (2000-2017)  Looked at different policies on both tobacco and alcohol control.    **W:**  Ecological study – unable to assess causation.  Limited and varied long-term data on tobacco consumption between countries. |

**School based programs N = 9**

| **Authors (year)**  **Title** | **Country of origin (published language)** | **Methods:**  **Type of study, study population, and sample size** | **Nature of upstream intervention** | **Outcome measures** | **Main findings** | **Conclusion** | **Strengths (S) and Weaknesses (W)** |
| --- | --- | --- | --- | --- | --- | --- | --- |
| Anderson, (2012)    Exploring Oral Health Disparities for Children in the City of Milwaukee  Note: *Grey Literature* | USA    (English) | Cross-sectional study.      Dataset of children aged 6-14 years constructed from Medicaid enrollment information and dental claims data provided to Marquette University’s School of Dentistry, N=359 979      Pre-intervention period: 2001-2003    Post-intervention period:2008-2009    Nine public schools in inner city Milwaukee targeted. | School based oral health program.    In 2006 Healthy Teeth=Healthy Kids partnership formed.    Key partners:  -Children’s Health Alliance of Wisconsin  -Children’s Hospital of Wisconsin Dental Center  -Marquette University School of Dentistry  -Medical College of Wisconsin  -Milwaukee Public Schools | Dental sealant application | Dental sealant application 3.9% vs 21.4% (2001 vs 2009)     Marginal effect analysis: Indicated that sealant application did increase because of treatment intervention. However, sealant use increased significantly amongst treatment and control groups, indicating that other factors not identified within the study may have also influenced sealant utilization. | Results of the logistic regression underlined that sealant application was greater amongst low-income children who attended schools with school based oral health programs compared to children who attended schools without school-based oral health programs. | **S:**  Large population sample    First study to comprehensively evaluate the impact of a school-based oral health programme in Milwaukee.    **W:**  Individual data unknown e.g., “inability to know precisely which schools Medicaid recipients attended”.    Some assumptions made on child age – exact dates not available due to privacy issues.    Unclear from the data how many sealants the children received e.g., one or multiple sealants? Information unknown    “Accurate and consistent cost and expenditure data relating to the sealant application process among school-based programmes” not available.    Child caries risk/ and other aspects of SES unknown. |
| Anttila et al., (2015)    Effect of national recommendation on sweet selling as an intervention for a healthier school environment | Finland    (English) | Longitudinal survey between 2007-2010    Upper level of Finnish comprehensive schools, children aged 13 -15 years.    N=480 in 2007  N=478 in 2010 | Health promoting schools.    National recommendation by the Finnish National Board of Education and National Institute for Health and Welfare (2007):    Ban of sweet products at school, availability of drinking water and provision/selling  of nutritionally appropriate snacks | Questionnaire data:  9 items in 2007  10 items in 2010    Exposure variable (0-10 points): Actions associated with increased oral health risk.    Enabling variable (0-10 points): Actions associated with oral health protection.    Policy variable (0-12 points): Policy decisions associated with exposure and enabling actions.    Exposure and policy variable trichotomized:    Exposing school (0-5); moderately exposing school (6-8); and non-exposing school (9-10)    Weak policy (0-4); moderate policy (5-6); and strong policy (7-12)    Mean values for exposure, enabling and policy calculated. | 2010 vs 2007:    Mean change of exposure, enabling and policy variables statistically significant:    Decreased exposure of pupils to sugar products (p<0.001)  Increased provision of oral health protection items (p=0.047)  Improved oral health promoting policies (p<0.001)    Significant decreased in the selling of candies (p<0.001); soft drinks (p<0.001); some sweet products (p<0.001) but not all sweet products (p=0.665).   Increased provision of healthy snacks (p=0.301) and drinking water (p=0.393) but results not statistically significant.    Significant changed in school policy: sugar selling guidance improved (p<0.001); pupils less likely to leave school premises (to buy unhealthy snacks) (p<0.001) and increased participatory decision making in relation to the selling of sugary snacks (p=0.05). | Improvement of school environments by means of national recommendation by having a reasonable effect on the selling of candy and soft drinks. | **S:**  Longitudinal study conducted over a 3-year period (2007-2010)    Representative sample    **W:**  Response bias    No agreed cut off points for variable categorisation. |
| Anttila et al., (2019)    School-level changes in factors related to oral health inequalities after national recommendation on sweet selling. | Finland    (English) | Ecological and longitudinal study    Combination of two independent studies from Upper Finnish  Comprehensive schools (children aged 13-15 years):    A) School Health Promotion Study (SHPS)- completed by pupils    B) School Sweet Selling Survey (SSSS) -completed by schools    Baseline data collected for SHPS (from 2006-2007) and SSSS (from 2007)    Post intervention data collected for SHPS / SSSS (2008-2009)    n = 360 | Health promoting schools:    National recommendation by the Finnish National Board of Education and National Institute for Health and Welfare (2007):    Ban of sweet products at school, availability of drinking water and provision/selling  of nutritionally appropriate snacks | Intermediate determinants by SEP    Intermediate determinants defined as:    Exposure, enabling and policy variables (see Antilla et al, 2015)    F1: Attitudes and access to intoxicants  F2: School health services  F3: School environment  F4: Home environment  Eating school meals  Unhealthy snacking at school  Eating habits in school    3 school level SEP groups formed: low; middle and high. | After intervention: Oral health promoting policies improved in low SEP schools only (p=0.02)    Statistically significant results also seen in oral health promoting actions (exposure) across all SEP levels (high/ middle p <0.001; low p=0.001)    Attitudes and access to intoxicants also significantly improved post intervention across all SEP groups (high/ middle SEP group p <0.001; low SEP p=0.006)    Decrease in exposure determinant across all school-level SEP groups: 38%, 35% and 39% in high, middle and low SEP groups, respectively.  Inverse social gradient in exposing pupils to sweet products: low SEP schools sold sweet products less often than middle and high school SEP schools.    Across all SEP levels, unhealthy snacking at school increased post intervention– however results were statistically insignificant(p>0.05) | National recommendations to make the school environment healthier was an effective tool. The decreased in sweet product selling was equal in every school-level SEP group suggesting that the national recommendation neither reduced nor increased the social gradient between schools’ sweet product selling. | **S:**  Longitudinal study    Study considered valid due to data collection via two independent data sets.    Study population considered representative and results generalisable.    **W:**  School Health Promotion Study (SHPS) is “traditional and respected” amongst upper Finnish schools leading to excellent response rate- however total response for this study small due to low number of participants who completed the School Sweet Selling Survey.  Self-reporting bias.    Study used data from secondary data analysis of SHPS study, therefore no individual response data from pupils. |
| Freeman et al., (2001)  Addressing Children’s Oral Health  Inequalities in Northern Ireland:  A Research-Practice-Community  Partnership Initiative | Northern Ireland    (English) | Cohort study    16 schools selected for study.    Year 1: Children aged 9 years,  n =118 (Boost Better  Breaks school-based policy (BBB school),  n =120 (control group)    Year 2: Children aged 10 years, n =99 (BBB schools) n =102 (control group)    For study analysis: n= 201 children (e.g., children who remained in Year 1/ Year 2) | Health promoting school:    Boost Better Breaks (BBB) break time policy.   Developed in partnership with dieticians; school meals advisors; teachers; health promotion advisors; and local dairy suppliers of school milk.    Ethos of the policy: Facilitate heathier eating within the school environment and make “healthy choice the easy choice” - specifically addressing the issue of unhealthy break time snacks and consumption of unhealthy drinks.  Participating schools must: Have a written policy to stipulate the consumption of milk and/or fruit only at break time. Furthermore, schools will agree not to sell high sugar/ fat snacks in the school environment and teachers must not reward pupils with candy snacks. | DMFT    Assessed using the British Association for the Study of Community Dentistry (BASCD) guidelines.    Single, independent, BASCD-calibrated community dentist examined all children in study. | N=201: In Year 1 of the study 33% of children were free of caries. In Year 2, 27% of children were caries free.    Mean DMFT index: 0.78 (Year 1) vs 1.05 (Year 2)    Significant increase in DMFT between Year 1 and Year 2 children in BBB schools: Mean DMFT index: 0.82 (Year 1) vs 1.12 (Year 2).    Significant increase in the number of filled teeth over time – particularly in children who attended low SES schools.    Children attending lower SES schools: Moderate increase in the mean number of sound permanent in children attending BBB schools:(Year 1: 12.44; 95%CI 11.46-13.40; vs Year 2: 14.71; 95%CI 13.49-15.99) compared to those attending control schools (Year 1: 13.75; 95%CI 12.48-14.65; vs Year 2: 14.65; 95%CI 13.52-15.80)    No significant effects of BBB participation on total caries experience (DMFT), mean number of missing/ filled teeth. | In the first two years of the program the program had a positive effect in increasing the mean number of sound teeth in children attending schools in low SES area. | **S:**   Study single blinded    **W:**  Non-random allocation of children into control/ BBB group    Possible misclassification using free school meals as a measure of SES.    Under powered study    Drop out of children from year 1 to year 2. |
| Kankaanpää et al., (2012)  Effect of national recommendations on the sale of sweet products in the upper level of Finnish comprehensive schools | Finland    (English) | Post-intervention study    Online questionnaires to Finnish upper comprehensive schools   2007 (before recommendation)  N = 480 schools    2008 (after recommendation)  N = 507 schools | Finnish National Board of Education (FNBE) and the National Public Health Institute (KTL) recommended to schools that quit regular selling of candies and soft drinks | Sales of sweet products | -Out of all responding schools, 56% sold sweets in 2007 and this decreased to 46% in 2006.  -For schools responding in both years, sold sweets decreased from 56% in 2007 to 50% in 2008.  -Larger schools more often sold sweet products and healthy products than did schools with fewer pupils (p<0.001) | The national recommendation was followed by some decrease in sale of sweet products. The national recommendation and pupils’ health were the main reasons stated for why schools had quit selling sweet products. | **S:**  Representative sample – generalizable to Finnish upper-level comprehensive schools. |
| Nery et al (2020)  Can the school environment influence oral health-related behaviours? A multilevel analysis of the Brazilian National Adolescent School-Based Health Survey 2015 | Brazil  (English) | Cross sectional study  Individual and school environment data obtained from 2015 Brazilian National Adolescent School based Health Survey  N=51 192 students aged 11-19 years  1339 public and private schools | Oral Health Promotion School Environment index (OHPSE)  OHPSE reflects the potential support of schools for oral health promotion based on characteristics of the school environment, such as sale of food with added sugars, general health promotion, prohibition of smoking and alcohol.  OHPSE index classified as high/intermediate or low | Oral health related behaviours:  -Dental visits (annually)  -Toothbrushing frequency (daily)  -soft drink consumption (weekly)  -sweets consumption (weekly)  -smoking experimentation  -alcohol experimentation | “Schools with ‘high OHPSE’ had **lower** prevalence of :  -low frequency of annual dental visits (PR = 0.94 [95% CI 0.90; 0.99])  -high weekly frequency of soft drink consumption (PR = 0.94 [95% CI 0.89; 0.99])  -high weekly frequency of sweet consumption’(PR = 0.96 [95% CI 0.93; 1.00]) than those with low OHPSE  Schools with intermediate OHPSE had **higher** prevalence of:  -low daily toothbrushing frequency’(PR = 1.12 [95% CI 1.03; 1.23])  -cigarette experimentation (PR = 1.08 [95% CI1.01; 1.16])  than those with ‘low OHPSE’.  Alcoholic beverage experimentation’ was not associated with OHPSE | “The potential support of schools for oral health promotion was associated with most of the oral health–related behaviours among adolescent students. Those attending schools with higher OHPSE scores reported a higher annual frequency of dental visits and a lower weekly frequency of soft drink and sweet consumption, while those in schools with intermediate OHPSE had a lower daily toothbrushing frequency and a higher rate of cigarette experimentation.” | **S:**  Large sample  **W:**  -Cross sectional design- cannot establish causation  -Survey did not contain questions on OH activities in schools  -Cigarette and alcohol experimentation may occur outside of schools  -Possible recall bias  -Limitations in software used for statistical analyses for complex data |
| Pallan et al. (2024)  School food policy in secondary schools in England and its impact on adolescents’ diets and dental health: The FUEL multiple- methods study | England  (English) | Observational mixed methods study  -36 State funded secondary schools  (13 with mandated school food standards compared with 23 with non-mandated school food standards)  -2453 pupils age 11-15 years  -151 school staff/governors  Quantitative 24 hr dietary recall, online survey to assess dental outcomes  Qualitative focus groups (pupils), qualitative interviews (staff/governors) in a subsample of 4 schools | School food standards/ School Food Plan implementation and costs | -Pupil free sugar intake:  1.At lunch  2.Across the school day  3.During 24hrs  -Additional nutritional outcomes  -Dental caries | Schools compliant with 64% of school food standards and 41% of School Food action plans implemented  No difference between schools with mandated or non-mandated school food standards in compliance but wide variation in annual costs  (mean cost £195 per pupil)  *Lunch intake*:  Lower lunch intake of free sugar among pupils from schools with mandated school food standards (mean difference:-2.78g 95% CI -4.66g to -0.90g)  *Total energy intake:*  No significant difference between schools  *Additional nutritional outcomes:*  Schools with mandated school food standards had lower fruit and vegetable consumption, higher consumption of sugar sweetened beverages over 24 hrs, and higher confectionary consumption during the school day  *Dental caries*:  No differences in caries outcomes | No evidence that school food standards legislation positively influences nutritional intake | **S:**  Multiple method approach, multi-level modelling  Diverse characteristics of included schools- representative of national population with respect to deprivation and ethnic proportions.  **W:**  Large amounts of missing data for cost analysis and school food plans; Disruption in data collection due to COVID-19 pandemic  Unable to recruit even numbers of schools with mandated food standards and school with non-mandated food standards |
| Rodrigues, Watt & Sheiham, (1999)  Effect of Dietary Guidelines on Sugar Intake and Dental Caries in 3-years-olds attending Nurseries in Brazil | Brazil    (English) | Cohort study   Children attending 29 non-fee-paying nurseries in Recife’s metropolitan area, aged 3 years (36 – 47 months).  Total sample, N = 510 children, with these details below:  12 nurseries with dietary guidelines, n = 245  17 nurseries without dietary guidelines, n = 265 | Nutritional guidance on reduced sugar intake    Guidance for this study based on expert nutritional information from the Ministry of Social Affairs in Brazil    International guidance: Non milk extrinsic sugars should not exceed 10% of total energy intake – 32.6g for a 3-year-old child | A new carious lesion    Base line data collected in September 1993    Follow up caries data collected from September to November 1994:     - WHO (WHO, 1987) caries diagnostic criteria used. - Daily total weight of sugar intake (g) - Daily frequency of sugar intake - Dietary information collected at nursery (using 6-day weighted food intake) and at home via the children's mother (using 24-hour food/drink recall method) | Significant differences in frequency and weight of sugar intake between children attending nurseries with guidance and no guidance (p<0.001)      Children attending nurseries with adopted guidance consumed less sugar in a day compared to children attending nurseries with no guidance (22.9 g vs 53.5g; p<0.001)    Children attending nurseries with no guidance were 4.87 times more likely to develop caries in 1 year compared to children attending nurseries with guidance (95%CI 1.99-11.92; p <0.001)    Children who consumed more than 32.6g of sugar per day were 2.75 times more likely to have high caries increment compared to children who consumed less than 32.6 g of sugar per day (95%CI: 1.29-5.85; p <0.001) | Children at nurseries with guidelines on sugar consumed less sugars and did that less frequently at the nursery than 3-year-old children attending nurseries without guidelines. | **S:**  As a cohort study, this study gave strong supportive evidence and emphasized non-milk extrinsic sugars as a major dietary cause of dental caries.  **W:**  Comparison of children attending nurseries not ideal as significant difference in household sizes and number of siblings – children attending adopting nurseries tended to come from larger households and have more siblings.  However, these factors were controlled during multivariate analysis. |
| Tubert-Jeannin, Leger & Manevy, (2012)  Addressing children's oral health inequalities: caries experience before and after the implementation of an oral health promotion  program | France    (English) | Intervention study     5 years old children attending public schools in Clermont-Ferrand with these details:  -15 deprived and semi-deprived schools.  6 non-deprived schools.  N = 620 | A school based OHP program for 3-5 years old in deprived and semi-deprived areas.   Key areas: improving tooth brushing; encouraging dental care; and delivering a range of educational activities for children and their carer’s | Mean DMFT in 2003 and 2009    Relationship between child dental health and household SES    Oral hygiene status | Mean DMFT was 1.18.  No major difference was observed between 2003 and 2009 except for an increase in the number of filled teeth.    In terms of area deprivation, mean dmft of children in deprived schools was 3-5 times higher than in the non-deprived schools.    In deprived schools, mean dmft was higher in those schools without the OHP program.    There was a cluster effect related to the school examined. Changes in dmft values  between 2003 and 2009 varied depending on the school examined. Average mean dmft value in the schools that benefited from the program tended to decrease.  Oral hygiene practices varied between schools.    OHP did little to improve oral health inequalities. | OHP program had done little to reduce disparities in oral health, even if dental status improved in four schools. | **S:**  Multiple factorial analysis was used.    Results consistent with other studies.  **W:**  Participation rate of the children in  each school varied greatly. This could infer bias as the dental status of non-participants could differ from participants.    Program was evaluated using cross-sectional studies and no longitudinal data was collected. |

**Health Warning Labels N = 3**

| **Authors (year)**  **Title** | **Country of origin (published language)** | **Methods:**  **Type of study, study population, and sample size** | **Nature of upstream intervention** | **Outcome measures** | **Main findings** | **Conclusion** | **Strengths (S) and Weaknesses (W)** |
| --- | --- | --- | --- | --- | --- | --- | --- |
| Aktan, (2018)  Strategic Packaging and Labelling Policies Affecting Purchase Intention  Towards Harmful Products | Turkey    (English) | Cross-sectional survey  Survey link administered online through a Turkish website that concentrates on higher educational issues (www.akademikpersonel.org). Participation was on a voluntary basis from individuals with age 17 years old and above.  N = 432 valid survey responses (n = 210 non-smokers, n = 212 smokers) | Strategic packaging and labelling of cigarette packs    Health Warning  Labels (HWL) on cigarette packs | Perceived health and social risk of smoking    Effectiveness of HWL in reducing the desire to smoke.    Believability of HWL | Perceived health risk of smoking did not affect the intention to abstain from smoking.    Perceived social risks of smoking significantly impacted smoking behaviors with the potential to deter smoking.    Health warning labels with plausible and believable messages elicited negative behavior towards smoking.    In addition, smokers were more influenced than non-smokers that HWLs are believable. | Believability of HWL content was an essential element to boost perceived health and social risks of smoking. The HWLs on cigarette packs were intended to increase the intention to abstain from smoking regardless of people smoking status. | **S:**  In contrast to mainstream research carried out in developed countries, this study was carried out in a less developed country with a high prevalence of smoking amongst the population. |
| Barrientos-Gutierrez et al., (2021)  Assessing cigarette packaging and labelling policy effects on early adolescents: results from a discrete choice experiment | Mexico    (English) | Discrete choice experiment (DCE)    Early adolescents aged 12–14 years, lived in 3 largest Mexican cities (Mexico City, Guadalajara and Monterrey) between October and November 2016. N = 4251. | Health warning labels (HWL’s) | Students asked to comment on 3 packs of cigarettes:    1. Which pack is most attractive?    2. If you were to smoke, which of these brands would you most like to smoke?    3. If you were to smoke, which of these brands will harm your health the most? | Students perceived packs as less attractive, less interesting, and more harmful if they had larger HWL’s compared to current smaller HWL’s (b=0.74, -0.377, 0.260, respectively)   Increasing the size of HWL’s on plain packs was more effective than small HWL’s in reducing attractiveness; interest in trying; and misconception of lower harm - result statistically significant.    Packs with plain packaging associated with lower attractiveness (b=- 1.695)    Relative importance of attributes:  -Pack attractiveness: Plain packaging had the biggest influence 43%; followed by HWL size 19%; HWL content 11%; and brand name 10%.  -Interest in trying cigarettes: brand name had biggest effect 34%; followed by plain packaging 29%; and HWL size 13%.  -Evaluating perception of harm of different cigarettes: Brand name (30%) and HWL size (29%) had largest effects; HWL content (17%) and flavor capsules (15%). | Larger sized HWLs or plain packaging could potentially reduce the appeal of cigarettes to early adolescents. | **S:**  The result of this study was consistent with previous experimental studies to determine the most effective HWL content across countries and in Mexico.  **W:**  Sample might not be representative of the general population.    Discrete choice experiment - hypothetical scenario    DCE stimuli were hypothetical.    DCE subject to reporting bias    Limited generalizability |
| Jevdjevic et al., (2021)    Front-of-Package Food Labeling to  Reduce Caries: Economic Evaluation | Germany – as reference case    (English) | Economic evaluation    Population aged: 14-79 years    Baseline data from German National Nutritional Survey II | Front of Package Food Labelling (FoPFL) | Caries lesions prevented.    Caries-related treatment costs avoided.    Disability-adjusted life years (DALYs)    Productivity losses averted | FoPFL in Germany over the period from 2017 to 2027 was estimated to prevent 2,370,715 (95% confidence interval [CI],  2,062,730–2,678,700) caries lesions.    Hypothetical treatment cost saving of €175.67 million (95% CI, 152.85–198.49)    FoPFL was estimated to avert 677.62 (95% CI, 589.59–765.65) DALYs.    A reduction of €27.33 million (95% CI, 23.78–30.88) in productivity losses  over 10 year. | Results suggested FoPFL could be a potential strategy to substantially reduce caries increment,  caries-related morbidity, and economic burden. | **S:**  The decision analytical microsimulation model was based on age/sex specific data.    The uncertainties in model parameters were quantified through deterministic and probabilistic sensitivity analyses.    **W:**  It was assumed that there would be only 1 surface restoration as a treatment option.    Disabilities resulting from the long-term consequences of caries such as tooth loss were not considered – underestimation of DALYs.    Cost of FoLP did not consider the cost involved with relabeling and marketing.    Limited generalizability |

**Sugar Sweetened Beverage consumption interventions N = 10**

| **Authors (year)**  **Title** | **Country of origin (published language)** | **Methods:**  **Type of study, study population, and sample size** | **Nature of upstream intervention** | **Outcome measures** | **Main findings** | **Conclusion** | **Strengths (S) and Weaknesses (W)** |
| --- | --- | --- | --- | --- | --- | --- | --- |
| Cleghorn et al., (2019)  Estimating the health benefits and cost-savings of a cap on the size of single serve sugar-sweetened beverages | New Zealand    (English) | Modelling intervention study  Adult participants’ dietary data from New Zealand Adult National Nutrition Survey (NZANS) conducted in 2008/2009)     N =4.4million in 2011 | Reducing sugar-sweetened beverages (SSB) portion to 250 ml  Base case model defined SSB as: carbonated soft drinks; fruit drinks; carbonated energy drinks; and sports drinks. Exclusion of fruit juices/ sweetened milks.    SSB information collected from a single 24-hour recall. | Change in energy intake and therefore BMI.    Potential cost savings and health gains in quality-adjusted life years (QALYs) | Base case scenario - modelling a 250ml SSB cap resulted in:     - Average decrease of 23.2ml of SSB and 44.2 kJ (10.5 kcal) per person/day modelled over 2 years. - Decrease of 0.22kg/ 0.08 BMI units per person modelled over 2 years. - Total health gain: 81 300 QALY’s (95%UI:64 500-101 000) - Total cost saving: NZ dollars; 1.62 billion (95% UI: 1.16 - 2.21 billion)     Health gain / cost impact of SSB 475 ml cap would result in substantially less benefit:     - 7030 QALY’s, 8.3% of the base case model - Cost saving of 136 million NZ dollars.     Health gain associated with a 20% serving size reduction:  74 100 QALY’S (87.3% of base case scenario)    Expanding definition of SSB to include fruit juice/ sweetened milk would result in:     - Total health gain: 116 000 QALY’s (33% higher than the base case) - Cost savings increase to 2.33 billion NZ dollars. | Improvement in health and cost savings could be gained through restricting the size of single serve of SSB. | **S:**  The study explored the likely effects of energy compensation through scenario analyses, and found that the intervention was still beneficial to health and was cost-saving even with 100% compensation.  **W:**   Lack of data on package size in survey – resulting in possible over estimation of health gains / cost savings    Data from 2008/2009 - SSB consumption may have increased from that point.    The NZANS survey had been shown to underestimate overall energy intake.    No modelling of tooth decay - under estimating the health impact of the intervention    Model based on adult consumption. |
| Cobiac et al. (2024)  Impact of the UK soft drinks industry levy on health and health inequalities in children and adolescents in England: and interrupted times series analysis and population health modelling study | England  (English) | Controlled interrupted time series analysis and modelling  Children and adolescents (0-17 years) over 10 yrs following both announcement (2016) and implementation (2018) of soft drinks industry levy (SDIL) | UK soft drinks industry levy (SDIL):  £0.18 per litre levy on drinks containing 5-8g sugars per 100ml  £0.24 per litre levy on drinks containing ≥ 8g sugars per 100ml | Modelled sugar reduction effects on:  1.BMI  2.Dental caries  3. QALYS (quality adjusted life years)  Lifetable model: long term impact of SDIL on:  -Life expectancy  -Slope index of inequality in life expectancy | SDIL reduced sugar from drinks by 15g per household per week (95%CI -10.3 to -19.7g)  Predicted outcomes from reduced sugar consumption in the first 10 years of implementation:  1. 64100 fewer overweight or obese children and adolescents (95%CI 54400 to 73400)  2. 3600 fewer caries (95%CI 946 to 6330)  3. Larger change in sugar purchase/impact on QALYS for children and adolescents living in deprived areas compared to those living in less deprived areas (IMD Q1: 11000 QALYS [95%CI 8370 to 14100], IMD Q5:1860 QALYS [95% CI 929 to 2890])  Small but significant reduction in slope index in life expectancy over the life course:  Men: 0.94% (95% CI -1.1 to -0.76) -6.6 days  Women: 0.76%(95% CI -0.9 to -0.62); -4.2 days | SDIL will result in:  -medium term reductions in caries  -medium term reductions in overweight/obesity  -long-term improvements in life expectancy  Greater benefits predicted for those living in more deprived areas- potential to narrow health inequalities. | **S:**  Medium- and long-term modelling of health outcomes reported based on real world intervention  **W:**  Modelling based on assumption that previous trends in sugar purchasing would have continued if SDIL not introduced- sugar consumption was starting to fall prior to SDIL.  Sugar consumption based on Kantar data which does not account for consumption by different individuals per household  Study assumes that reduction in purchase of sugar-based drinks equates to reduced sugar consumption  Study does not account for sugar-based drinks purchased outside of the home  Modelling does not account for long term impact of SDIL on sugar content in drinks |
| Gupta et al., (2021)  Modelling the impact of increase in sugar prices on dental caries in India | India    (English) | Modelling analysis with Hypothetical cohort.     N=1000 individuals. These individuals were followed up starting from 12 years to 75 years of age. | Sugar/ SBB price increase by 20%    Two scenarios evaluated:    Scenario 1= sugar/ SBB consumption at current level.    Scenario 2 = sugar/SBB consumption at 20% increase prices | 1.Caries reduction and prevention of tooth loss    2. Economic impact: Treatment cost saving in Indian rupees (INR) and US dollars | A 20% rise in sugar prices would lead to:     - Reduction in caries incidence by 1.32 (95%CI 1.16-1.28) and prevent the loss of at least 1 tooth due to caries during the lifetime within a group of 33 individuals. - At a population level: the prevention of 938.59 million carious lesions (95% CI: 752.23 million to 1130.24 million) and the loss of 27.96 million teeth in the current population of 12 years old, over the lifetime. - Consequently – potential cost saving of INR 3116.3 billion (US $: 42.69 billion, 95% CI: 35.68-50.19 billion) of caries attributed treatment cost.     A 20% rise in the cost of SSB would result in the:     - The prevention of 138.06 million carious lesions (95% CI:112.26 to 164.54 million) and the loss of 4.81 million (95%CI 3.93-5.69 million) teeth during the lifetime. - Consequently – potential cost saving of INR 776.19 billion (US $:10.63 billion, 95% CI: 8.89-12.36 billion) of caries attributed treatment cost in India. | Increasing the cost of sugar or SSB will reduce the daily intake of sugar which will reduce caries incidence and progression. | **S:**  Cohort study can potentially establish the relationship between the SSB policy and oral health condition changes.  **W:**   Estimates for treatment costs/ mean dental caries are likely to be under-estimated.    The model did not account for the possibility of “cross-price elasticities and opportunity costs of paying the tax”.    The study did not consider the impact of dental factors e.g., dental fee changes, supplier inducement. |
| Hernández-F, Cantoral & Colchero, (2021)  Taxes to Unhealthy Food and Beverages and Oral Health in Mexico: An Observational Study | Mexico    (English) | Observational study    Data of individuals aged 1 to 99 years was collected from 2 sources of information:    A) Administrative records from oral health outpatient visits.  Information collected from the Ministry of Health. Validated data from 2007-2018. Patient first and subsequent visit analyzed.    B) SIVEPAB system – for dental caries data. Reporting system that monitors the state of oral health in Mexico. Active since 2009.    N= 2,648,893 | SSB tax implemented in Mexico in January 2014    8% tax on non-essential energy dense food and one peso per litre tax to SSB | A) Change in outpatient visits related to dental caries    B) Change in dental caries experience (DMFT/ dmft) after tax implementation | For outpatient visits related to caries:    Negative pre-tax trend showing a reduction of 88.1 visits/ month (statistically not significant); immediate significant positive change after tax implementation (41 197 visits); followed by a negative post-tax trend showing a reduction on the number of outpatient visits to 2920.5 per month (statistically not significant)    Reduction in number of teeth with caries experience/ probability of having experienced dental caries across all age groups (post intervention) except in those aged 0-5 years.    For dmft, immediate reduction of 2162.9 individuals and an average post-tax reduction of 107.5 individuals having experienced dental caries per quarter.    For DMFT, immediate reduction in the number of individuals experiencing dental caries of 6147.5 and a post-tax trend reduction of 393.6 individuals per quarter.    For dmft, results showed an immediate reduction of 0.146 in the mean number of teeth with caries after tax implementation and an average reduction of 0.004 per quarter in the mean number of teeth with caries (significant at 10%).    For DMFT, results also showed a negative post tax trend with an average quarterly reduction of 0.026 in the mean number of teeth with caries. | Positive impact of the implementation of taxes on unhealthy food and beverages on the oral health of Mexicans. The study results showed monthly reductions in the post-tax period for outpatient visits related to caries. In addition, there was a reduction in the probability of having experienced dental caries for all age groups, except for children under 5 years old.  The reduction was also seen in the number of individuals having experienced dental caries and mean number of teeth with caries experiences. | **S:**  First study in Mexico that estimated changes in oral health associated with taxation.    The study applied different methodological approaches to analyze information at the individual and population level.    **W:**  Data sample large, but not considered representative.     Data on outpatient visits did not provide information on oral health status.  No data on eating habits    Study only applicable to those who had access to health services. |
| Jevdjevic et al., (2019)  The caries-related cost and effects of a tax on sugar-sweetened beverages | Netherlands – reference case    (English) | Cost effective analysis.    Dutch population aged 6 to 79 years. The data was obtained from Statistics Netherlands.    Population from Statistics Netherlands | Sugar sweetened beverages taxes | Caries free tooth years pers person    Population level prevention of caries lesion    Tax revenues    Avoided treatment costs.    Administrative costs of SSB | A 20% SSB taxation would result in an average of 2.13 caries-free tooth years per person and, on population level, prevention of 1,030,163 caries lesions.    The intervention saved a total of €159.01 million of dental care expenditures. The estimated lifetime tax revenues €3.49billion) were larger than administrative costs for taxation, €37.3 million.  For boys aged 6 -12 years, the intervention would be most beneficial: 162,213 caries lesions prevented and 6.17 million caries-free tooth years gained.  For girls and women, the benefits in terms of caries-free tooth years per person are lower compared with boys and men, 1.64 and 2.61 caries-  free tooth years, respectively. | SSB taxation may potentially improve oral health and reduce the caries related economic burden. Benefits would be best for younger age groups. | **S:**  This study was the first study to assess the potential benefits of SSB on dental care.    **W:**  Not a global study – did not look at country specific estimates for SSB.    Did not include the substitutions of sugar containing food.    Prediction study not an evaluation of implemented SSB. |
| Lamloum et al (2025)  Effects of a sugar-sweetened beverages tax on caries in Italy: a modelling study | Italy  (English) | Modelling analysis on the effectiveness of sugar sweetened beverage taxation (SSB) on caries experience of 12-year-old children and health care costs | Two scenarios based on implementation of 2025 20% SSB taxation, amounting to €10 per hectolitre for finished products and €0.25 per kg for products prepared to be used after dilution.  Scen 1: hypothetical implementation of sugar tax in 2008 compared to 2017 epidemiological survey data  Scen 2: Projection of the effects of the 2025 tax over 10 years to 2034, based on caries data derived from the 2017 epidemiological survey | Caries experience (DMFT)  QALYS(quality adjusted life years)  Direct dental health care costs  Indirect costs- lost earnings due to care | Scen 1:  -0.05 reduction of DMFT (DMFT fall from 1.82-1.77)  -€18.5 million savings from combined direct and indirect costs  -Intervention costs €24,520 per QALY gained (below €40,000 per QALY limit which indicates cost effectiveness)  Scen 2:  -0.07 reduction of DMFT (DMFT fall from 2.35 -2.28)  -€38.7 million savings from combined direct and indirect costs  - Additional costs €31,933 per QALY gained  Greater impact from both scenarios seen in southern Italy, where caries experience is higher and incomes lower | SSB taxation can reduce caries and associated health care costs, particularly in disadvantaged areas. | **S:**  Robust medium term modelling scenarios explored  **W:**  Projection coefficient does not account for local variation in taxation effects  Some trajectories applied originated from literature- no specific to Italy  Costs are based on pre COVID-19 data  Demographic estimates based on birth data extending back to 2002  Modelled dental treatment pathways did not account for interventions to save teeth from extraction (e.g. endodontics)  Data adapted from 2007 epidemiological survey rather than 2017 due to missing data |
| Petimar et al. 2023  Changes in dental outcomes after implementation of the Philadelphia beverage tax | USA  (English) | Retrospective difference in differences analysis of electronic dental record data  Patients residing in Philadelphia between 2014-2016 and after-tax implementation in 2019  Analyses included adults/older children over the age of 15 and children under the age of 15  N=83,260 | Philadelphia beverage tax implementation 1^st^ January 2017  1.5 cent per oz taxation on sugar sweetened beverages and artificially sweetened beverages | Caries experience (DMFT/DMFS) | The number of DMFT did not change across the general population following tax implementation for adults/older children or younger children  Decreases in DMFT for:  -Adults/older children (-0.18, 95% CI: -0.34 to -0.03)  -Younger children (-0.22, 95% CI: -0.46 to 0.01)  in families receiving Medicaid | “The Philadelphia beverage tax was not associated with reduced tooth decay in the general population, but it was associated with reduced tooth decay in adults and children on Medicaid, suggesting potential health benefits for low-income populations.” | **S:**  Real life data analyses  **W:**  -Measurement error with DMFT- codes may not reflect decay, inaccuracies in recording  -Possible purchasing of beverages outside of Philadelphia may have attenuated associations  -Modest sample size limited by exclusion of outcomes in 2020 due to COVID19 pandemic  -Stratified analyses not possible due to small sample sizes |
| Rogers et al., 2023  Estimated impact of the UK soft drinks  industry levy on childhood hospital  admissions for carious tooth extractions:  interrupted time series analysis | UK  (English) | Observational  Routinely Collected Health Data Study.  Children aged 0–18 years in England attending a National Health Service (NHS) hospital.  The data was obtained from Hospital Episode Statistics (HES) | In March 2016, the UK government announced a soft  Drinks industry levy (SDIL) with the aim of reducing sugar intake.  The two-tier  Tax was implemented in April 2018. This SDIL was designed to encourage manufacturers to  reformulate their drinks rather than pass the tax on to  the consumer. Manufacturers of soft drinks containing  ≥8 g of sugar/100 mL are subject to a levy of £0.24 /L  and those with ≥5 to <8 g of sugar/100 mL are taxed at  £0.18 /L. Soft drinks containing <5 g/100 mL sugar are  not liable for the levy and 100% fruit juices, powder to  make drinks, milk and milk-based  drinks and drinks with  1.2% alcohol by volume or more are exempt irrespective of sugar content. | Changes in the incidence rates of hospital admission for  carious tooth extraction in children in the 22 months  following the implementation of the UK SDIL. | Compared with the counterfactual scenario, there was a relative reduction of 12.1% (95% CI 17.0% to 7.2%) in hospital admissions for carious tooth extractions in all  children (0–18 years). Children aged 0–4 years and 5–9 years had relative reductions of 28.6% (95% CI 35.6% to 21.5%) and 5.5% (95% CI 10.5% to 0.5%), respectively; no change was observed for older children. Reductions  were observed in children living in most IMD areas regardless of deprivation. | The UK SDIL was associated with reductions in incidence rates of childhood hospital admissions for  carious tooth extractions, across most areas regardless of deprivation status and especially in younger children. | **S:**  Previously, there were no studies that have examined associations between SSB taxes and changes in  childhood hospital admissions for carious tooth extractions,  an indicator of severe dental decay. This study addressed the knowledge gap and limited evidence of an associations between SSB taxes and childhood hospital admissions.  W:  A comparable control group was not available. |
| Urwannachotima et al., 2020)  Impact of sugar-sweetened beverage tax on dental caries: a simulation analysis | Thailand    (English) | Qualitative system dynamic modelling project.  Explore the impact of new SSB tax policy on dental caries among Thai adults from 2010 to 2040 in Thai population aged 15 years and older.  n = 7 informants were interviewed for 30 to 60 minutes in-person individually using a semi-structured, open-ended questionnaire. | A.SSB tax policy scenario (2018-2040)   Aggressive policy scenario consisting of:   - SSB tax policy scenario - Non-SSB sugar consumption scenario - Dental care service utilization scenario | Caries prevalence – DMFT    Caries defined using WHO criteria.    Caries categorized as 4 groups: Very low DMFT; Low DMFT; Moderate DMFT; High DMFT | Base case scenario: prevalence of dental caries amongst the Thai population projected to increase from 61.3% in 2010 to 74.9% by 2040.    Implementation of SSB tax policy is expected to decrease the prevalence of caries by only 1% by 2040.    Implementation of aggressive policy is projected to decrease caries prevalence by 21% by 2040.    Mean DMFT values for those aged 15-34 years by 2040:  Base case scenario: 2.60  SSB scenario: 2.58  Aggressive scenario: 2.22    Mean DMFT values for those aged 35+ years by 2040:  Base case scenario: 2.92  SSB scenario: 2.89  Aggressive scenario: 2.15    Projected distribution of caries by DMFT severity from 2010 to 2040:    Base case scenario:  Low DMFT: Decrease from 9.95 million to 9.76 million.  Moderate DMFT: Increase from 8.37 million to 9.87 million  High DMFT: Increase from 12.56 million to 19.79 million.    Compared to base case estimates, by 2040, implementation of SSB policy is projected to increase the number of individuals with low DMFT by 1%; decrease moderate DMFT by 0.1%; decrease high DMFT by 3%.    Compared to base case estimates, by 2040, implementation of aggressive policy is projected to increase the number of individuals with low DMFT by 18%; decrease moderate DMFT by 9.4%; decrease high DMFT by 46%. | Implementation of the proposed tiered SSB tax in Thailand was expected to reduce SSB consumption among both the low- and high- income groups. The highest impact observed in the longer term due to higher SSB tax rates in later years.  SSB tax alone is unlikely to have meaningful impact on oral health unless it is accompanied with a comprehensive public health policy that aims to reduce total sugar intake from non-SSB sources | **S:**  Active engagement of stakeholders in the development of modelling project  Results can be used to inform policy prior to implementation.    **W:**  Change in the projected population will affect the number of people by DMFT groups.    Due to the lack of longitudinal data, the study was unable to estimate and use “evidence-based transition rates across different DMFT groups by age, gender and SEP” leading to possible under/ over point estimates. |
| Urwannachotima et al., 2020)    System dynamics analysis of  dental caries status among Thai  adults and elderly | Thailand    (English) | System dynamics modeling  Data used to populate the model was obtained from the Thai national oral health survey in 2000, 2006, 2012 and Thailand Official Statistics Registration.  Three policies scenarios were experimented in the model: health promotion policy, dental personnel policy and affordable dental care service policy.  The base sample was Thai population aged 15 years and older in 2010, N = 50.4 m.  The projected Thai population 15 years and older in 2020 (N = 53.36 m), 2030 (N = 54.10 m), 2040 (N = 52.60 m). | SSB tax policy scenario | Relationship between dental caries, sugar consumption and SSB | Implementing SSB taxation alone will not achieve impact anticipated of improving dental caries without the input of health education, and affordability of oral health services.    Four insights into an SSB:  Sugar consumption in Thailand has increased.    Taxing SSBs without increasing tax. On other high sugar content products will likely produce a substitution effect.    SSB needs to be combined with oral health education and oral health capacity.    SSB tax policy has the capability to fund the implementation of oral health promotion programmes. | The main insight from this study suggested that implementing SSB tax alone will not achieve the desired oral health outcomes, without combining it with other non-tariff interventions such as oral health education and improved access to oral health services. | **S:**  Engagement with stakeholders    Results can be used as evidence to support SSB an adapted/holistic implementation.    **W:**  Lack of involvement of consumers in modelling who would be able to give insight into potential taxes. |

**Financial incentives N=1**

| **Authors (year)**  **Title** | **Country of origin (published language)** | **Methods:**  **Type of study, study population, and sample size** | **Nature of upstream intervention** | **Outcome measures** | **Main findings** | **Conclusion** | **Strengths (S) and Weaknesses (W)** |
| --- | --- | --- | --- | --- | --- | --- | --- |
| Olmstead, Rosen, Hole (2025)  Assessment of a health system-integrated children’s savings account and financial coaching program serving low-income Moms and babies in Texas | USA  (English) | Randomised controlled trial  Low-income mothers from 3 months pregnant to 9 months post-partum  140 participants randomised to control (N=59) or intervention (N=81) until child turned 18 months old | Early Bird (EB): a health system integrated program to provide financial incentives to mothers to achieve healthy targets:  **Intervention** EB=  Financial incentives:  -$250 for enrolling in the EB programme and opening a tax advantaged children’s savings account (CSA)  -$25 contribution into CSA for attending 6-week post-partum check  -$75 for paediatric dental visit by 12 months of age  -$75 for 6 x well-check visits by age 15 months  -$30 for 2 financial coaching sessions  **Control:**  At the end of the study, participants given:  -$470 in CSA  -$30 in gift cards to attend 2 x financial coaching  Sessions  -$10 to attend group session about college preparation | *Medical milestones:*  -Maternal 6-week post-partum check  -Paediatric dental visit by age 12 months  -6 x well-check visits by age 15 months  *Non-medical milestone*:  -1 x financial coaching session | No significant differences between both groups in achieving any medical milestone  EB program increased the likelihood of participants attending financial coaching session from 26-33% (p<0.001) | No evidence that EB increased the likelihood of low-income mothers achieving medical milestones  Financial incentives offered in EB may be too low to significantly impact behaviour | **S:**  First study to assess health system integrated CSA programme contributions which are dependent of medical/dental milestones  RCT study  Sensitivity analyses completed to overcome anomalies with randomisation and recruitment  **W:**  Small sample  13 participants dropped out of the study prior to completion  Recruiting and randomisation procedures compromised- some participants ‘randomised’ in a block  Not generalisable to the wider population of mothers |
