# Supplementary material for: Upstream Interventions to Promote Oral Health and Reduce Oral Health Inequalities: A Scoping Review
Source: Community Dent Oral Epidemiol. 2025 Dec 29;54(2):146–62. doi: 10.1111/cdoe.70049 (PMC13000981; doi:10.1111/cdoe.70049)
Supplement: Supplementary file 4 — Table S2: Data extraction table for systematic reviews. [file CDOE-54-146-s004.docx]

**Table S2. Data extraction table for systematic reviews**

**Wider determinants (mixed interventions) N = 5**

| **Author (Year)**  **Title** | **Aim of the systematic review**  **Nature of intervention** | **Number of studies included and**  **countries included** | **Date range for literature searches** | **Author’s conclusions** | **Strengths (S)**  **Weakness (W)** |
| --- | --- | --- | --- | --- | --- |
| Bambra et al., (2010)  Tackling the wider social determinants of health and health inequalities: evidence from systematic reviews | Aim:  The study aimed to gather evidence synthesis on the effects of health and health inequalities of interventions aimed at influencing the wider social determinants of health.  Nature of intervention:   - Housing/living environment interventions: social changed (e.g., rent assistance); environmental interventions (housing restructuring); area-based firearm restrictions. - Work environment interventions: Dusseldorf health circle discussion groups; reorganization of work policies; privatization; legal regulations - Transport interventions: traffic calming measures; new road building; population level interventions; legal policy; speed cameras. - Access to health / social care interventions: improving cultural access; geographic access. - Unemployment/ welfare interventions: supported/ pre-vocational employment training; welfare rights advice; welfare to work interventions aimed at people with health conditions/ disability. - Agriculture/food interventions: monetary interventions - Water sanitation interventions; water fluoridation (0.05- 1.5ppm F) | N=30 systematic reviews  North America  Europe  Australasia  Japan  Note: there was limited detail information regarding the countries included in the study. | January 2002- April 2007 | Generally, the effects of interventions on health inequalities remained unclear.  Weak systematic evidence based on tackling social determinants.  Tentative systematic review evidence to support certain categories of interventions to reduce inequalities in disadvantaged groups particularly in the housing/ work environment sector.  Fluoridated water conclusion: Fluoridation up to 1 ppm had no adverse effects on bone fracture/ bone density/ or bone strength.  No report on health inequalities. | **S:**  Research strategies piloted and revised.  Searches conducted by experienced specialist staff members at the York Centre for Reviews and Dissemination.  Leading public health journals were hand searched and review authors contacted.  **W:**  Too few systematic reviews conducted.  Challenge to locate systematic reviews was difficult and time consuming.  Searches were- lack of sensitivity/  specificity  All reviews might not be located.  USA data was fundamentally different from European data, due to differences in welfare systems, therefore findings from USA data might not be transferrable to European policies. |
| Garzón-Orjuela et al., (2020)  An overview of reviews on strategies to reduce health inequalities | Aim:  This study aimed to identify strategies/interventions that facilitate the reduction of health inequalities.  Nature of Intervention:   - Delivery arrangements of health care - Financial arrangements of health care - Governance arrangements - Implementation strategies | N=97 reviews  87 countries were included in the study:  Afghanistan; Argentina; Armenia; Australia; Austria; Banco Oeste; Bangladesh; Belgium; Benin; Bolivia; Botswana; Brazil; Burkina Faso; Burma; Caledonia; Cameron; Canada; Chile; China; Colombia; Congo; Croatia; Denmark; Ecuador; Ethiopia; Finlandia; France; Gabon; Germany; Ghana; Greece; Guatemala; Guinea; Haiti; Honduras; Hungary; India; Indonesia; Iran; Ireland; Israel; Italy; Jamaica; Kenya; Korea; Kyrgyzstan; Lesotho; Madagascar; Malaysia; Malawi; Mexico; Mozambique; Nepal; Netherlands; New Zealand; Nicaragua; Níger; Nigeria; Norway; Pakistan; Panama; Papua New Guinea; Peru; Philippines; Poland; Portugal; Rwanda; Saint Lucia; Scotland; Senegal; Sierra Leone; Spain; South Africa; Sweden; Switzerland; Taiwan; Tanzania; Tajikistan; Thailand; Trinidad and Tobago; Turkey; Uganda; United Kingdom; United States; Vietnam; Zimbabwe; Zambia | January 2014 – July 2019 | In vulnerable populations multi-component and personally tailored interventions were more effective at the reduction of inequalities in oral health.  Implementation strategies were identified as effective in oral health care in immigrant population. | **S:**  This study included general population, vulnerable populations, and minority populations.  **W:**  High variability of the reviews s and outcomes |
| Hayre et al. (2025)  Health impacts of the Sure Start programme on disadvantaged children in the UK: a systematic review | Aim:  To investigate the child health outcomes from the Sure Start programme  Nature of intervention:  **Sure Start:** a national early years intervention programme established from 1999.  Sure Start Children’s Centres (SSCC) were targeted in deprived communities and controlled by local authorities, with the aim of promoting health equity and wellbeing through multicomponent interventions focused around physical health, social development and neurodevelopmental disorders, based on five services:   1. Outreach and home visiting 2. Support for families and parents 3. Good quality play, learning and childcare 4. Primary and community healthcare, advice about child and family health 5. Support for children and parents with specialised needs | N= 9 reviews  1 country included: UK | January 2024 – October 2024 | Sure Start services were associated with:  -reduced obesity rates in older children  -reduced hospitalisations in older children (20% reduction by age 11)  -Reduced accidental injury  -Improved dental health-positive oral health behaviours and awareness of dental hygiene  -Increased breastfeeding uptake  -Increased use of health support worker intervention  -Reduction in clinical symptoms of ADHD and conduct disorder and improved screening for developmental delay  “This study examine the evidence of the Sure Start programme as an example of an early-years community-based intervention to mitigate health inequity among disadvantaged children…. .We found significant evidence for Sure Start’s effect on the domains of physical health and neurodevelopmental disorders…...” | **S:**  Broad search strategy, including grey literature  **W:**  Study heterogeneity limited comparisons  Limited studies focused on children over the age of 12- only able to generalize the findings to children under this age  Focused on Sure Start only- unable to generalize to other early years community-based interventions outside of the UK |
| Lorenc et al., (2013)  What types of interventions generate inequalities?  Evidence from systematic reviews | Aim:  This study aimed to gather evidence from systematic reviews regarding interventions that may increase the risk of inequalities by disproportionately benefiting less disadvantaged groups - “intervention generated inequalities” (IGIs)  Nature of intervention:   - Education, communication, and information (e.g., printed communication materials, media campaigns, and health warnings on tobacco products) - Multi-component settings-based interventions (e.g., multi-component school-based interventions, school-based interventions, multi-component community-based interventions, Community-based interventions) - Resource provision and fiscal interventions (e.g., free fruit provision in schools, free folic acid supplements, and tobacco price increases - Regulatory and workplace interventions (e.g., restrictions on tobacco sales to minors, restrictions on tobacco advertising, workplace smoking bans - Other interventions (e.g., Housing, transport, unemployment and welfare, agriculture and food, water and sanitation, adult education | N=12  There was no information regarding countries included in the study. | Not provided | The following interventions might increase the risk of IGIs:   - Mass media campaigns - Workplace smoking bans   The following interventions might reduce the risk of inequalities:   - Reducing price barriers - Fiscal interventions such as tobacco pricing - Structural workplace interventions | **S:**  Study contributed to the small evidence base on IGIs.  **W:**  Limited evidence based on IGI’s.  Majority of studies on IGIs focused on health behaviors as opposed to health status outcomes.  Relevant papers might had been excluded due to search criteria. Selective reporting bias due to reliant on “authors’ characterization”.  Only included reviews that reported the effect of an intervention on inequalities between SES groups and did not included reviews that targeted low SES groups. |
| O’Dwyer et al., (2007)  Do area-based interventions to reduce health inequalities work? A systematic review of evidence | Aim:  To review the efficacy of area or locational interventions that aimed to reduce inequities.  Nature of intervention:  Area based interventions aimed at reducing health inequities | N = 24  2 countries were included in the study:  UK and USA | 1 November 2003 to 14 September 2004 | There was some evidence that area-based interventions reduce inequities.  Area based interventions worked best when there was a change in the physical environment | **S:**  Inclusions of grey literature  **W:**  Success of the interventions was difficult to gage due to the use of multiple strategies; inadequate evaluation reports; variation in the size or type of area; insufficient funding for implementation; policy changes over the course of the program; and lack of  long-term evaluations**.**  English only publications  Differences in political context made difficult to compare studies. |

**Caries intervention N = 3**

| **Author (Year)**  **Title** | **Aim of the systematic reviews**  **Nature of intervention** | **Number of studies included and**  **countries included** | **Date range for literature searches** | **Author’s conclusions** | **Strengths (S)**  **Weakness (W)** |
| --- | --- | --- | --- | --- | --- |
| Mariño, Khan & Morgan, (2013)  Systematic Review of Publications  on Economic Evaluations of Caries  Prevention Programs | Aim: This study aimed to perform a literature review of Economic Evaluations (EEs) of dental caries prevention programs.  Nature of intervention:   - preventive strategies, such as dental sealants and water fluoridation - mixed interventions (interventions that included a combination of preventive strategies such as dental sealants, fluoride varnish, gel, preventive dental programs, and oral health education. | N=63  9 countries were included in the study:  Australia; Canada; Chile; Finland; Japan; Spain; Sweden; UK; USA | January 1975 – April 2012 | The most common preventative strategies were dental sealants (13),  Community Water Fluoridation (12), and  mixed interventions (12)  The most common form of EEs was the use of cost-benefit analysis. | **S:**  Large date range and 4 languages included.  **W:**  Poor quality of reporting as methodological weaknesses  Failure of papers to justify conducting EEs.  No grey literature searched.  Mapping of evidence rather than interpretation of current literature |
| Shen, Bernabé & Sabbah, (2021)  Systematic Review of Intervention Studies Aiming at Reducing  Inequality in Dental Caries among Children | Aim:  This study aimed to systematically review the evidence on intervention programs aiming at reducing inequality in dental caries among children.  Nature of intervention:  oral health promotion/education, and prevention (topical fluoride and water fluoridation) to reduce caries among children across different socioeconomics groups. | N=13  11 countries were included in the study:  Australia; Brazil; Canada; England; Finland; France; Germany; Japan; Republic Ireland; South Korea; USA | Start of database - December 2020 | Overall findings suggested that whole population interventions such as water fluoridation are more likely to reduce inequalities in caries than target populations/ individual interventions. | **W:**  Publication bias – grey literature considered but only published articles included in the review.  Overall quality of papers moderate  Meta-analysis not conducted due to high heterogeneity of included papers. |
| Van Meijeren-van Lunteren et al. (2023)  Caries Preventive Interventions and oral health inequalities: a scoping review | Aim:  To identify European public health interventions that report their effect on dental caries across different social groups  Nature of intervention: mixed interventions including-  Fluoridated water, salt, tablets, mouthwash, dental screening, supervised toothbrushing, healthy snacking | N=14  4 countries were included in the study:  France, Germany, Ireland, United Kingdom, | Start of database -March 2021 | “Interventions that contain early approaches, with a high frequency, approaching multiple levels of influence, and including at least the broader organizational or public policy level may have the potential to reduce oral health inequalities among children from birth to young adolescence.” | **S:**  Comprehensive search of multiple databases, including non-randomized and grey literature  **W:**  Limited number of studies assessed and some of low quality.  Findings limited to European populations  Socially disadvantaged groups were defined differently in each study  Sample sizes may be underpowered |

**Obesity N = 1**

| **Author (Year)**  **Title** | **Aim of the systematic reviews**  **Nature of intervention** | **Number of studies included, and countries included** | **Date range for literature searches** | **Author’s conclusions** | **Strengths (S)**  **Weaknesses (W)** |
| --- | --- | --- | --- | --- | --- |
| Cairns et al., (2015)  Weighing up the evidence: a systematic review  of the effectiveness of workplace interventions to tackle  socio-economic inequalities in obesity | Aim:  To systematically review if inequalities in  obesity can  be reduced by workplace interventions.  Nature of intervention:  Behavioral interventions in the workplace to reduce inequalities in obesity. | N=18  6 countries were included in the study:  Australia; Brazil; Chile; Germany; Korea; USA | From start of database to 11 October 2012 | Workplace physical interventions had the potential to reduce inequalities in obesity by targeting low-income groups. However, the evidence base was small, heterogeneous, predominantly from the USA and low quality. Further high-quality studies are needed. | **S:**  Extensive international  literature search  Broad inclusion/exclusion criteria to capture observational/ experimental data.  **W:**  -Inclusion of studies that reported proxies for body fat.  -Small number of experimental studies.  -Large body of data from the USA and less data from other countries.  -Few environmental studies found.  -Lack of studies on organizational interventions and inequalities on obesity.  -Heterogeneity of interventions/study designs.  -Only 10% of studies double screened. |

**Fluoridation N = 7**

| **Author (Year)**  **Title** | **Aim of the systematic reviews**  **Nature of intervention** | **Number of studies included, and countries included** | **Date range for literature searches** | **Author’s conclusions** | **Strengths (S)**  **Weaknesses (W)** |
| --- | --- | --- | --- | --- | --- |
| Cagetti et al., (2013)  A systematic review on fluoridated food in caries  prevention | Aim:  To evaluate the scientific evidence of the effects of fluoride intake via food  on the occurrence of carious lesions.  Nature of intervention:  Fluoridated food (milk; sugar; salt) for caries prevention | N=3  3 countries were included in the study:   1. China (milk) 2. Indonesia(sugar) 3. Sweden (milk) | Start of electronic database to 31 March 2011 | There was some evidence that fluoridated milk is effective in caries prevention, however the scientific evidence was low. | **S:**  First study to analyze the effectiveness of food fluoridation for the prevention of caries.  **W:**  Literature on the effectiveness of fluoridated food was scarce.  No studies on adult/elderly |
| Iheozor-Ejiofor et al., (2024)  Water fluoridation for the prevention of dental caries (Review)  Cochrane Database of Systematic Reviews | Aim:  -To evaluate the effects of initiation or cessation of community water fluoridation (CWF) programmes for the prevention of dental caries.  -To evaluate the association of water fluoridation (artificial or natural) with dental fluorosis.  Nature of intervention:  -Initiation or cessation of CWF for the prevention of dental caries  -Association of CWF with dental fluorosis. | N = 157  47 countries were included in the study:  Antigua; Argentina; Australia; Brazil; Canada; Chile; China; Cuba; Denmark; Estonia; Ethiopia; Finland; Germany; Ghana; Greece; Hungary; Iceland; India; Indonesia; Iran; Ireland; Italy; Japan; Lithuania; Malaysia; Mexico; Namibia; Netherlands; New Zealand; Poland; Portugal; Saudi Arabia; Serbia; Singapore; South Africa; Sri Lanka; Sudan; Sweden; Switzerland; Taiwan; Tanzania; Thailand; Turkey; Uganda; United Kingdom (England, Scotland, Northern Ireland, Wales); USA; Venezuela | - The Cochrane Oral Health Group's Trials Register (to 12 July 2022) - The Cochrane Central Register of Controlled Trials (CENTRAL; The Cochrane Library 2023, Issue 8) - MEDLINE via OVID (1946 to 16 August 2023) - EMBASE via OVID (1980 to 16 August 2023) - Proquest (all databases; to 16 August 2023) - Web of Science Conference Proceedings (1990 to 23 August 2023) - ZETOC Conference Proceedings (1993 to 12 July 2022) - US National Institutes of Health Trials Register (23 August 2023) - The WHO Clinical Trials Registry Platform (to 23 August 2023) | “Contemporary studies indicate that initiation of CWF may lead to a slightly greater reduction in dmft and may lead to a slightly greater increase in the proportion of caries‐free children, but with smaller effect sizes than pre‐1975 studies. There is insufficient evidence to determine the effect of cessation of CWF on caries and whether water fluoridation results in a change in disparities in caries according to socioeconomic status. “ | **S:**  The Grades of Recommendation, Assessment, Development and Evaluation Working  Group (GRADE) approach was used to ensure the quality of the  evidence within the review.  **W:**  The arbitrary cut-off based on a priori clinical judgment was used in this study: water with a fluoride concentration of 0.4 ppm or less was classified as non-fluoridated.  However, this study acknowledged that this cut-off might be high for equivalence of non-fluoridation in  hot climates. |
| McDonagh et al., (2000)  Systematic review of water fluoridation | Aim:  To review the safety and efficacy of  fluoridation of drinking water.  Nature of intervention:  Water fluoridation | N=214  Countries not provided. | Start of database to February 2000 | The quality of evidence was low to moderate.  There was reduction in the incidence of caries, however smaller reduction than previously reported.  The range (median) of mean change in decayed, missing, and filled permanent/ primary teeth was 0.5 to 4.4 (2.25) teeth.  Prevalence of fluorosis was highly associated with concentration of fluoride in drinking water. At a fluoride level of 1 ppm an estimated 12.5% (95% CI: 7-21.5%) of the exposed population would present with fluorosis that can impact the appearance. | **W:**  Lack of appropriate study design/ analysis:  Studies did not always present an analysis.  No adjustment for confounding factors.  Variance in estimates not reported.  Observer bias likely for fluorosis studies.  Overestimation of fluorosis due to differing criteria.  Statistical heterogeneity.  Publication bias. |
| McLaren & Singhal, (2016)  Does cessation of community water fluoridation lead to an increase in tooth decay? A systematic review  of published studies | Aim:  To review published research on the  impact of Cessation of community water  fluoridation (CWF) cessation on dental caries.  Nature of intervention:  Community water fluoridation interventions focused on children, age range 3 years to 15 years or grade 2 (approximate age 17 – 18 years) | N = 15 instances of cessation (interventions)  13 countries were included in the study:  Brazil; Canada; China; Cuba; Czechoslovakia; Finland, Germany; Japan; Netherlands; Scotland; South Korea; USA; Wales (UK); | The publication year ranged from 1962 to 2014. | This systematic review highlighted an increase in dental caries post-CWF cessation than otherwise.  There were absent of evidence on the equity implications of discontinuing CWF as none of the studies examined equity of impact. | **S:**  Study offered comprehensive approach to identify knowledge gaps.  There were very limited previous publications focused on the decision-making circumstances around CWF cessation.  Inclusion of non-English publications  **W:**  This study focused on children only; there may be opportunities to explore/ study the impact of CWF cessation on other demographic groups (e.g., adults and elderly)  Did not include grey literature. |
| Ran & Chattopadhyay, (2016)  Economic Evaluation of Community Water Fluoridation: A Community Guide Systematic Review | Aim:  To review economic evaluation of community water fluoridation (CWF) which include community fluoridation benefit analysis and community fluoridation cost analysis.  Nature of intervention:  community water fluoridation (CWF) to reduce dental caries across populations | N=10  4 countries were included in the study:  Australia; Canada; New Zealand; USA | January 1995-November 2013 | The economic benefit of CWF exceeded the intervention cost.  Benefit analysis: Different measures of dental cost always lower in communities with water fluoridation | **S:**  Supported other works regarding cost benefits of CWF.  Used of peer review and grey literature  **W:**  Conducted in high income countries (western) and none from low-income countries.  Economic benefits were estimated not actual costings. |
| Rosário et al., (2021)  External control of fluoridation  in the public water supplies of Brazilian cities as a strategy against caries: a systematic review  and meta‑analysis | Aim:  To perform a systematic review to assess the adequacy water fluoride concentration in Brazilian cities.  Nature of intervention:  Water fluoridation | N=12 for qualitative analysis  N=10 for quantitative analysis  Brazil | Start of database to 10 August 2020 | Mean concentration of fluoride ranged from 0.17 to 0.89 ppm.  Meta-analysis (pooled prevalence) found that over half of water samples analyzed had a fluoride level outside the acceptable range (56.6%; 95% CI 45.5-67.3), with high heterogeneity.  “Fluoride levels in the public water supply in several Brazilian cities are inadequate to guarantee the anticaries benefits and safety from fluorosis”. | **S:**  First systematic review with meta-analysis on this topic  Extensive search strategy; no restriction on language and publication date; grey literature included.  **W:**  High heterogeneity of results due to different methods of water collection/ sampling criteria: sample size; frequency; storage.  Review covered only 3 out of 5 Brazilian regions resulting in the exclusion of 2 other regions. |
| Yeung, (2008)  A systematic review of the efficacy and safety  of fluoridation | Aim:  To evaluate  caries reducing benefit and potential health risks of providing fluoride systematically (via addition to water, milk, salt) and the use of topical fluoride agents.  Nature of intervention:  Fluoride addition and fluoridation, such as:   - Fluoride addition to water, milk, and salt. - Topical fluoride agents (toothpaste, gel, varnish and mouth rinse). | N = 77  Countries not provided. | Database from 1996 to December 2006, and limited to English language publications | Fluoridation of drinking water remained the most effective and socially equitable means of achieving community-wide exposure to the caries prevention effects of fluoride.  Mild dental fluorosis associated with water fluoridation and topical fluoride but aesthetically concerning.  No association of any intervention with an increase of: cancer risk; fracture or osteoporosis risk | **S:**  A 4^th^ study in reviewing the effects of fluoride.  Comprehensive selection of fluoride products  Not a comprehensive search strategy as no controlled vocabulary used in searches |

**School based programs N = 2**

| **Author (Year)**  **Title** | **Aim of the systematic reviews**  **Nature of intervention** | **Number of studies included, and countries included** | **Date range for literature searches** | **Author’s conclusions** | **Strengths (S)**  **Weaknesses (W)** |
| --- | --- | --- | --- | --- | --- |
| Hofmann, Flaschberger & Felder-Puig, (2014)  Effects of school health promotion on social  inequalities. A systematic review | Aim:  To review the effectiveness of public health interventions to health promotion in schools.  Nature of intervention:  school-based interventions to improve health outcomes, health-related behaviours, or health-related knowledge | N = 25 including 5 systematic reviews    7 countries were included in the study:  Australia; Austria; Canada; Finland; Germany; Norway; USA; Cross-country | 1.January 2008–31October.2012 | Most interventions targeting nutrition and physical activity resulted in increased gender inequality, but rarely increased ethnic and socioeconomic inequalities. Regarding dental health (3 studies), two interventions reduced, and one had no effect on social inequalities. Mixed findings for mental health and substance use. | **S:**  Examined inequalities by SEP, ethnicity, and sex.  **W:**  Only few RCTs exist, high heterogeneity regarding outcomes and interventions; only EU/OECD countries included. |
| Malhotra et al. (2025)  Effectiveness of school-based approaches for reduction of sugar and sugar sweetened beverages in children: a systematic review and meta-analysis | Aim:  To identify the effectiveness of school-based interventions (SBI) in the reduction of sugar sweetened beverages (SSB) consumption in school setting  Nature of intervention:  -Physical activity with additional components  -nutritional education/handbook promotion/awareness  -menu assessment, school food changes, school environment changes  -cognitive behavioural technique utilisation  -nurse delivered counselling  -Parenting skills/role modelling  -decreasing screen media use | N= 44 (14 included in meta-analysis of the primary study outcome)  12 Countries were included in the study: USA, Australia, Finland, Canada, Denmark, Germany,  China, Brazil, New Zealand, UK, Spain, Netherlands | 1996 – September 2021 | -Interventions that target various levels of influence e.g., peers +school environment were more effective than those aimed at individuals, but all of the interventions were effective in decreasing SSB consumption.  Meta analysis:  When assessing mean SSB outcomes, significant reduction in SSB intake immediately after intervention (SMD -0.72, 95% CI -1.01 to -0.43)  “School-based interventions have demonstrated encouraging outcomes in decreasing sugary soft drink intake  among teenagers.. The included trials demonstrated a moderate  quality of evidence, suggesting that educational and behavioural interventions yielded only a modest effect in reducing sugar sweetened  beverage (SSB) consumption.” | **S:**  Data synthesis and first study on SBI to include meta-analyses  Included studies with single and multi component interventions  **W:**  Clinical heterogeneity due to differences in study settings participants and strategies used in different studies  Possible publication bias  Did not include studies published in a language other than English |

**Sugar Sweetened Beverage consumption interventions N = 2**

| **Author (Year)**  **Title** | **Aim of the systematic reviews**  **Nature of intervention** | **Number of studies included, and countries included** | **Date range for literature searches** | **Author’s conclusions** | **Strengths (S)**  **Weaknesses (W)** |
| --- | --- | --- | --- | --- | --- |
| Hashem, He & Macgregor, (2019)  Effects of product reformulation on sugar intake and health—a  systematic review and meta-analysis | Aim:  To determine the effect of product reformulation measures on sugar intake and health outcomes  Nature of intervention:  Product reformulation (cap and trade, choices program, and industry-led reformulation) | N = 16  8 countries were included in the study:  France; Greece; Israel; Netherlands; South Africa; Spain; United Kingdom; USA | 1990-early 2016 | Product reformulation to reduce sugar content could reduce sugar intake in individuals and consequently improve population health.    Evidence from RCT suggested that sugar reformulation can reduce sugar intake and body weight. | **S:**  Grey literature included.  Meta-analysis included.  Assessed the quality of evidence through Grades of Recommendation, Assessment, Development and Evaluation Working  Group (GRADE).  **W**  Quality of evidence was low as a lot of evidence drawn from modelling studies.  Only included English language publications.  The way products were administered to participants was different across trials. |
| von Philipsborn et al., (2019)  Environmental interventions to reduce the consumption of sugar sweetened beverages and their effects on health (Review) | Aim:  To assess the effects of environmental interventions on the consumption of sugar-sweetened beverages (SSB), sugar sweetened milk and health outcomes and report any adverse outcomes.  Nature of intervention:   - Labelling interventions - Nutrition standards in public institutions - Economic tools - Whole food supply interventions - Retail and food service interventions - Action across sectors - Home-based interventions | N = 58  14 countries were included in the study:  Australia; Brazil; Canada; Chile; Germany; Italy; Mexico; Netherlands; New Zealand; Norway; Peru; United Kingdom; USA; Vietnam | Beginning of databases – 24 Jan 2018 | Overall, there were effective and scalable interventions addressing SSB consumption at a population level.  Measures which change the environment could help people drink less SSB, such as:   - Traffic light labels - Price increases on SSBs in restaurants, stores, and leisure centers - Promotion of healthier beverages in supermarkets - Government food benefits scheme that could not be used to buy SSB. - Community campaigns focused on SSB. | **S:**  Included large populations and range of ages.  Median length of follow-up was 10 months.  **W:**  47/58 studies included were in English-speaking countries.  Did not include studies on taxation of SSB.  Limited discussion on the interventions effect on social disadvantage |

**Oral health promotion N = 3**

| **Author (Published Year)**  **Title** | **Aim of the systematic review**  **Nature of intervention** | **Number of studies included, and countries included** | **Date range for literature searches** | **Author’s conclusions** | **Strengths (S)**  **Weaknesses (W)** |
| --- | --- | --- | --- | --- | --- |
| Satur et al., (2010)  Review of the evidence for oral  health promotion effectiveness | Aim:  To review the effectiveness of oral health promotion  Nature of intervention:  Fluorides and other preventive interventions | N = 21  Countries not provided. | Post 1998 until 2000. | The evidence from this study supported the continued fluoridation of water supplies, and fluoride toothpaste programmes where there was no CWF.  This study also supported the interventions aimed at early childhood and aged care settings, smoking cessation and capacity building with non-oral health care providers. | **S:**  Inclusion of grey literature and quality ranking of papers  **W:**  English language only papers  Few upstream interventions reviewed |
| Gwynn et al., (2020)  Community based programs to improve the oral health of Australian Indigenous  adolescents: a systematic review and  recommendations to guide future strategies | Aim:  To  systematically examine the quality of community engagement and  oral health interventions for indigenous adolescents globally  Nature of intervention:  Community based oral health programmes targeting indigenous adolescents aged 10-19 years | N=9  6 countries were included in the study:  Australia; Brazil; Canada; Nigeria; Taiwan; USA | From 1990 to 2019 | Eight out of nine studies reported statically significant improvements in at least one component of oral health.  However, very few good qualities peer reviewed international studies targeting the indigenous adolescent population.  Absence of Indigenous community engagement evident, questioning the effectiveness/ long term sustainability of existing community programs. | **W:**  Number of studies were small.  Studies included a sample population with an age range above the 10-19 inclusion age criteria.  Limited number of culturally competent/ effective community health programs targeting Indigenous  Australian population. |
| Ricomini Filho et al., (2021)  Community interventions and strategies for caries control in Latin American and  Caribbean countries | Aim:  To summarize community interventions and strategies to control caries in Latin America and Caribbean Countries (LACC)  Nature of intervention:   - Restricting sugar consumption - Using fluoride - Placement of dental sealants | N=37  20 countries were included in the study:  Argentina; Belize; Bolivia; Brazil; Cuba; Chile; Colombia; Costa Rica; Dominican Republic; Ecuador; El Salvador; Guatemala; Haiti; Honduras; Mexico; Nicaragua; Panama; Peru; Uruguay; Venezuela | Start of database to 17.06.2020 (restricting sugar consumption /fluoride)  Start of database to 28.07.2020 (pit/fissure sealants) | Community interventions for sugar restrictions were based on education(Brazil); food supplementation(Chile); and sugar replacement(Belize)  Main fluoride-based strategies: water fluoridation; salt fluoridation; milk fluoridation; toothpaste and to a lesser extent: mouthwash; fluoride (APF) gels; and varnishes. Studies related to fluoride use mainly located in: Argentina; Belize; Bolivia; Brazil; Chile; Colombia; Costa Rica; Cuba; Dominican Republic; Ecuador El Salvador; Guatemala; Haiti; Honduras; Mexico; Nicaragua; Panama; Paraguay; Peru; Uruguay; and Venezuela.  Studies related to occlusal sealants mainly located in Brazil; Chile; Colombia; Costa Rica; Peru, Mexico and Venezuela. | **W:**  Intervention strategies on restricted sugar intake were scarce in the LACC. |

**Tobacco control policies N=1**

| **Author (Published Year)**  **Title** | **Aim of the systematic review**  **Nature of intervention** | **Number of studies included, and countries included** | **Date range for literature searches** | **Author’s conclusions** | **Strengths (S)**  **Weaknesses (W)** |
| --- | --- | --- | --- | --- | --- |
| Saad et al. (2024)  Effectiveness of tobacco advertising, promotion and sponsorship bans on smoking prevalence, initiation and cessation: a systematic review and meta-analysis | Aim:  To synthesise evidence of the effectiveness of tobacco advertising, promotion and sponsorship (TAPS) bans on smoking prevalence, initiation and cessation.  Nature of the intervention:  TAPS bans, including point of sale, and partial bans | N=16  70 countries were included in the study, including:  USA, Finland, Ireland, England, Scotland, Chile, Austria, the Netherlands, France, Italy, Portugal, Spain, New Zealand, Australia, Belgium, Bulgaria, Croatia, Cyprus, Czech Republic, Denmark, Estonia, Greece, Iceland, Latvia, Lithuania, Malta, Norway, Poland. Portugal, Romania, Slovakia, Slovenia, Sweden, Ukraine, Congo, Ghana, Madagascar, Mauritania, Senegal, Togo, Uganda, Antigua, Bolivia, Jamaica, Panama, Paraguay, Peru, St Lucia, St Vincent, Trinidad and Tobago, Venezuela, Iraq, Qatar, Tunisia, Albania, Georgia, Kyrgyzstan, Moldova, Montenegro, San Marino, Serbia. Tajikistan., Bhutan, Maldives, Brunei, Kiribati, Mongolia, Palau, Samoa, Vanuatu. | Inception -10 March 2023  Updated search 11 April 2024 | **Smoking prevalence:**  TAPS bans associated with 20% lower odds of smoking (pooled OR: 0.80, 95%CI 0.68 to 0.95)  **Smoking initiation:**  TAPS bans associated with 37% reduced risk (pooled HR:0.63, 95% CI 0.48 to 0.82)  **Smoking cessation**:  No significant association of TAPS and cessation  “The available evidence suggests that TAPS bans influence smoking behaviour, which strengthens calls for the implementation and enforcement of these policies.” | S:  Comprehensive search including grey literature and meta-analysis  Strengthens evidence of TAPS bans, including the impact of point-of-sale display bans  Some studies included data collected over 8 years  W:  High number of cross-sectional studies- unable to make causal inferences  Some longitudinal studies subject to attrition bias  Moderate risk of bias with some studies- confounding, self-reporting of outcomes  Heterogeneity in meta-analysis |
